# Bousmekines A-E, New Alkaloids from Two *Bousigonia* Species: *B.angustifolia* and *B. mekongensis*

**DOI:** 10.1007/s13659-020-00278-6

**Published:** 2020-11-03

**Authors:** Zong-Qing Huo, Qian Zhao, Wen-Tao Zhu, Xiao-Jiang Hao, Yu Zhang

**Affiliations:** State Key Laboratory of Phytochemistry and Plant Resources in West China, Kunming Institute of Botany, Chinese Academy of Sciences, Kunming, 650201 PR China

**Keywords:** *Bousigonia*, *B. angustifolia*, *B. mekongensis*, Monoterpenoid indole alkaloids, Bousmekines A-E

## Abstract

**Abstract:**

Four new monoterpenoid indole alkaloids, bousmekines A-D (**1**–**4**), and one new pyranopyridine alkaloid, bousmekine E (**5**), were isolated from the twigs and leaves of *Bousigonia angustifolia* and *Bousigonia mekongensis*. Their structures including absolute configurations were elucidated by a combination of MS, NMR, ECD calculation, and single-crystal X-ray diffraction analysis. Compound **2** was an eburnea-type MIAs characterized by a rare chlorine atom while **5** possessed a novel pyranopyridine moiety. Their cytotoxicities against several human cancer cell lines were evaluated and compound **1** exhibited significant cytotoxicity with IC_50_ values of 0.8–7.4 μM.

**Graphic Abstract:**

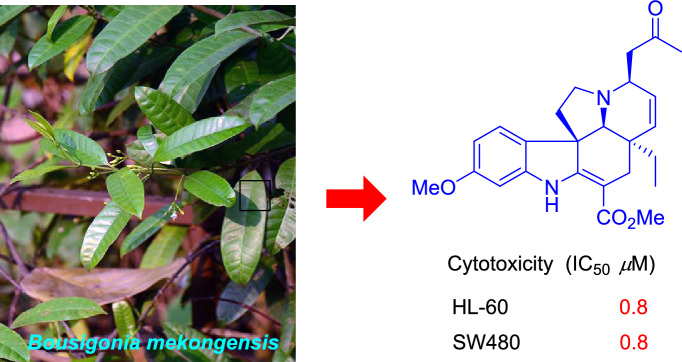

**Electronic supplementary material:**

The online version of this article (doi:10.1007/s13659-020-00278-6) contains supplementary material, which is available to authorized users.

## Introduction

**Fig. 1:**
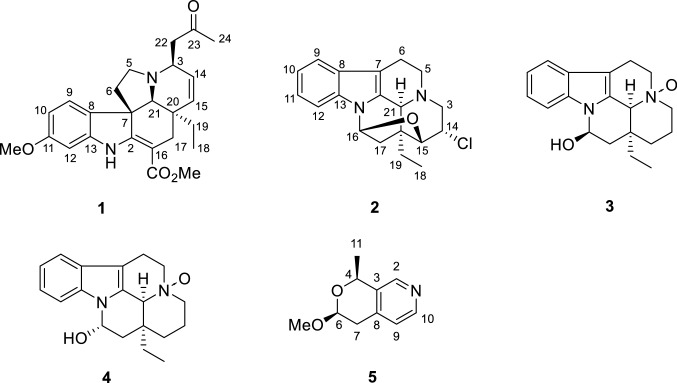
Structures of compounds **1**–**5**

Monoterpenoid indole alkaloids (MIAs), constituting a large family of secondary metabolites, are mainly distributed in plants of Apocynaceae family [1–3]. MIAs exhibited intriguing biological effects, such as anticancer [4], antibacterial [5], and lysosome generating activities [6]. Due to their structural complexity and biological diversity, MIAs have long been attractive objects by chemists and pharmacologists [1]. The genus *Bousigonia* (Apocynaceae family) comprises only two species, *B. angustifolia and B. mekongensis*, both of them are distributed in southwestern China [7]. Previous chemical studies on this genus have resulted in the identification of more than 100 alkaloids, primarily consisted of aspidosperma, eburnea, and aspidosperma-eburnea type MIAs [8–12]. In order to further investigation on novel and bioactive MIAs and proffer new vision into the constitutions of the two *Bousigonia* species, their alkaloidal extracts were investigated and four new MIAs (**1**–**4**) and one new pyranopyridine alkaloid (**5**) was isolated. This paper herein describes the isolation, structural elucidation and the cytotoxicities of the isolates (Fig. 1).

## Results and Discussion

### Structure Elucidation of the Compounds

Bousmekine A (**1**) was obtained as an optically active colorless solid, [*α*]_D_^20^ − 22 (*c* 0.1, MeOH). Its molecular formula, C_25_H_30_N_2_O_4_, was established by HRESIMS ion at *m/z* 423.2281 [M + H]^+^ (calcd 423.2278), corresponding to nine degrees of unsaturation. IR absorptions implied the presence of carbonyl (1676 cm^−1^) function.

**Fig. 2:**
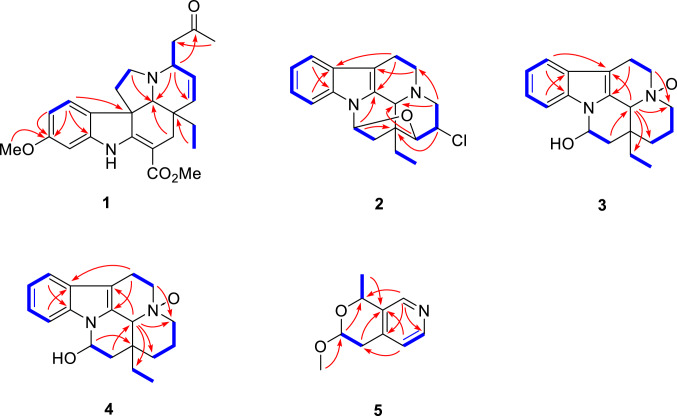
Key HMBC (arrow) and ^1^H-^1^H COSY (bold) correlations of compounds **1**–**5**

**Fig. 3:**
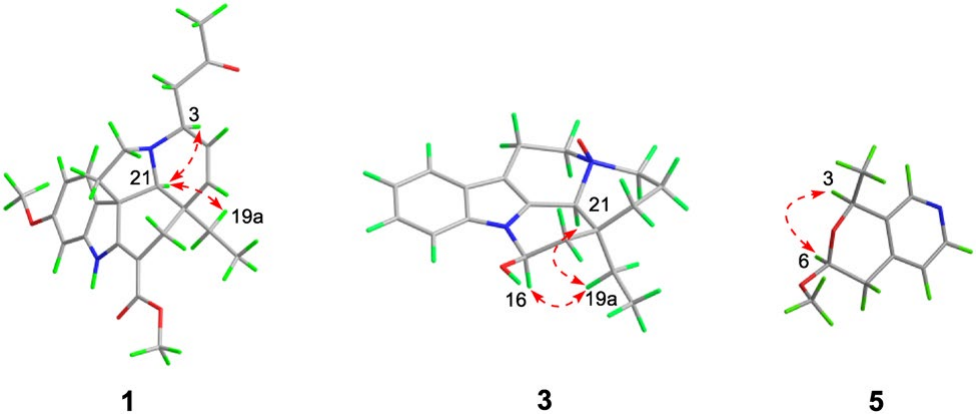
Key ROESY correlations of compounds **1**, **3**, and **5**

**Fig. 4:**
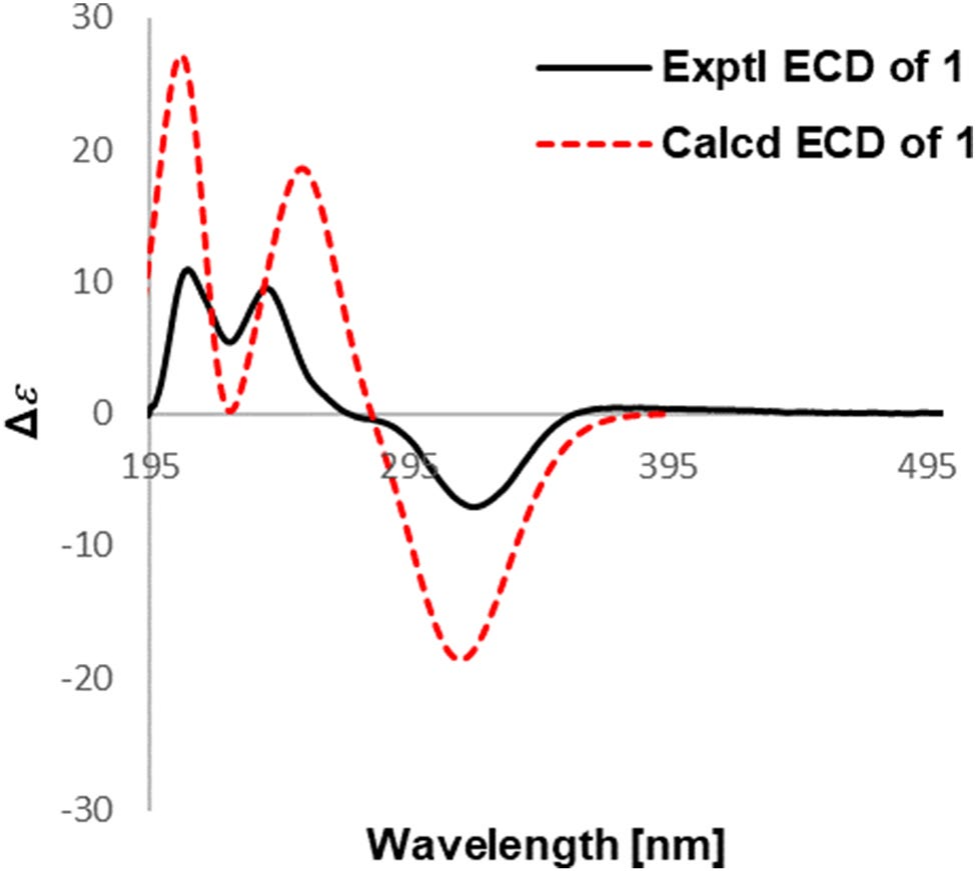
Experimental and calculated ECD of **1**

Detailed analysis of its NMR data (Tables 1 and 2) indicated that compound **1** had a high similarity with that of 3*α*-acetonyltabersonine [13], except for the presence of an additional methoxy (*δ*_H_ 3.81; *δ*_C_ 55.5) and a nonprotonated quaternary carbon (*δ*_C_ 160.0). The key HMBC correlations of OMe (*δ*_H_ 3.81), H-9 (*δ*_H_ 7.17, d, *J* = 8.4 Hz) and H-10 (*δ*_H_ 6.44, dd, *J* = 8.4, 2.4 Hz) to C-11 (*δ*_C_ 160.0) indicated that the methoxy group was located at C-11 (Fig. 2), and thus established the planar structure of **1** as 11-methoxy derivative of 3*α*-acetonyltabersonine. 2D NMR spectra (HSQC, HMBC, and ^1^H-^1^H COSY) confirmed the other parts of **1** were the same as that of 3*α*-acetonyltabersonine (Fig. 3). The ROESY correlation of H-3/H-21, and of H-21/H-19a indicated that these protons were co-facial and arbitrarily assigned as *α*-oriented. Therefore, the relative configurations of **1** was assigned as (3S*, 7R*, 20R*, 21S*)-**1**. The ECD calculation results for (3*S*, 7*R*, 20*R*, 21*S*)-**1** matched well with its experimental ECD spectrum finally established the absolute configuration of **1** (Fig. 4).

**Table 1:** ^1^H NMR spectroscopic data for compounds **1–5** (*δ* in ppm, *J* in Hz)

No	**1**^*a*^	**2**^*a*^	**3**^*b*^	**4**^*b*^	**5**^*c*^
2					8.40 (1H, s)
3a	3.96 (1H, m)	2.43 (1H, m)	2.94 (1H, d, 11.4)	3.03 (1H, m)	
3b		2.53 (1H, dd, 13.8, 1.8)	3.25 (1H, td, 11.4, 3.6)	3.44 (1H, td, 12.6, 3.0)	
4					4.96 (1H, q, 6.6)
5a	2.98 (1H, dd, 14.4, 6.6)	3.25 (1H, dd, 14.4, 7.8)	3.68 (1H, m)	3.72 (1H, m)	
5b	3.08 (1H, dd, 14.4, 6.6)	3.41 (1H, m)	3.88 (1H, m)	3.86 (1H, m)	
6a	2.04 (1H, m)	2.68 (1H, ddd, 14.4, 7.8, 1.8)	3.08 (1H, m)	3.03 (1H, d, 12.0)	4.78 (1H, dd, 8.4, 3.0)
6b	1.98 (1H, m)	2.88 (1H, ddd, 14.4, 7.8, 1.8)	3.08 (1H, m)	3.13 (1H, m)	
7a					2.78 (1H, dd, 16.8, 8.4)
7b					2.91 (1H, dd, 16.8, 3.0)
9	7.17 (1H, d, 8.4)	7.48 (1H, d, 7.8)	7.47 (1H, d, 8.4)	7.47 (1H, d, 8.4)	7.10 (1H, d, 4.8)
10	6.44 (1H, d, 8.4, 2.4)	7.14 (1H, t, 7.8)	7.19 (1H, t, 8.4)	7.20 (1H, t, 8.4)	8.33 (1H, d, 4.8)
11		7.20 (1H, t, 7.8)	7.13 (1H, t, 8.4)	7.11 (1H, t, 8.4)	1.59 (3H, d, 6.6)
12	6.42 (1H, d, 2.4)	7.38 (1H d, 7.8)	7.74 (1H, d, 8.4)	7.50 (1H, d, 8.4)	
14a	5.82 (1H, dd. 10.2, 4.8)	3.92 (1H, m)	1.44 (1H, d, 13.8)	1.52 (1H, m)	
14b			2.47 (1H, m)	2.48 (1H, m)	
15a	5.74 (1H, d, 4.8)	3.91 (1H, m)	1.08 (1H, td, 13.8, 3.6)	2.00 (1H, td, 14.4, 3.6)	
15b			1.52 (1H, d, 13.8)	1.52 (1H, d, 14.4)	
16		5.98 (1H, d, 5.4)	5.58 (1H, dd, 9.6, 4.2)	6.00 (1H, br d, 4.8)	
17a	2.12 (1H, d, 15.6)	2.41 (1H, m)	1.93 (1H, dd, 9.6, 4.2)	2.08 (1H, m)	
17b	2.58 (1H, m)	2.41 (1H, m)	2.25 (1H, m)	2.20 (1H, dd, 13.8, 4.8)	
18	0.65 (3H, t, 7.2)	1.09 (3H, t, 7.2)	1.04 (3H, t, 7.2)	1.02 (3H, t, 7.8)	
19a	0.92 (1H, m)	1.89 (1H, m)	2.25 (1H, m)	2.08 (1H, m)	
19b	1.03 (1H, m)	2.34 (1H, m)	2.35 (1H, m)	2.55 (1H, m)	
21a	3.04 (1H, s)	4.24 (1H, s)	4.43 (1H, s)	4.37 (1H, s)	
22a	2.57 (1H, m)				
22b	2.78 (1H, dd, 15.6, 8.4)				
24	2.25 (3H, s)				
OCH_3_	3.81 (3H, s)				3.48 (3H, s)
COOCH_3_	3.77 (3H, s)				

^*a*^600 MHz in CDCl_3_

^*b*^600 MHz in CD_3_OD

^*c*^600 MHz in (CD_3_)_2_CO

**Table 2:** ^13^C NMR spectroscopic data for compounds **1–5** (*δ* in ppm)

No	**1**^*a*^	**2**^*a*^	**3**^*b*^	**4**^*b*^	**5**^*c*^
2	166.0	134.4	130.0	128.6	146.8
3	52.4	46.8	58.7	59.0	135.9
4					70.7
5	51.7	50.6	70.5	71.1	
6	42.5	18.8	20.6	20.7	100.1
7	55.3	110.0	106.3	105.8	34.7
8	131.4	130.0	128.4	128.4	142.3
9	122.5	118.6	119.3	119.3	124.4
10	105.5	120.7	123.6	123.4	148.4
11	160.0	122.2	121.6	121.5	21.5
12	96.3	110.4	113.6	112.4	
13	144.3	137.2	139.4	138.0	
14	126.5	54.9	17.4	17.7	
15	132.6	80.6	26.1	26.9	
16	90.7	81.9	76.5	74.4	
17	31.3	40.7	43.1	41.2	
18	7.9	9.5	8.4	8.5	
19	30.0	26.7	31.8	32.0	
20	37.2	44.3	39.9	37.8	
21	64.9	56.4	72.8	73.6	
22	46.6				
23	207.7				
24	30.6				
OCH_3_	55.5				55.9
*C*OOCH_3_	168.9				
COO*CH*_*3*_	50.9				

^*a*^150 MHz in CDCl_3_

^*b*^150 MHz in CD_3_OD

^*c*^150 MHz in (CD_3_)_2_CO

**Fig. 5:**
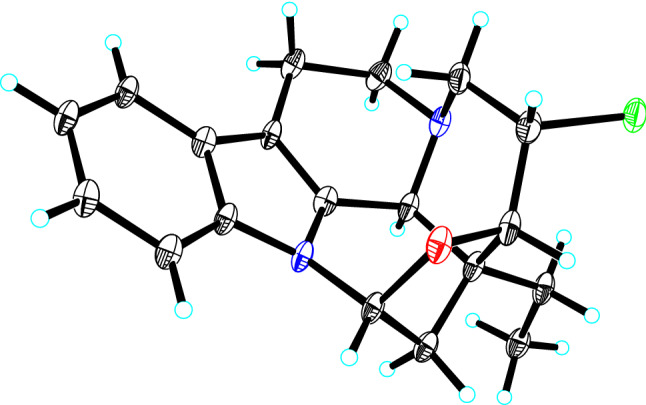
X-ray crystal structure of **2**

Bousmekine B (**2**) was obtained as an optically active colorless crystal, [*α*]_D_^20^ =  − 98 (*c* 0.1, MeOH). Its molecular formula was determined to be C_19_H_21_N_2_OCl by HRESIMS ion at *m/z* 329.1414 [M + H]^+^ (calcd 329.1415), suggesting 10 unsaturation degrees. The ^13^C and DEPT spectra suggested that **2** possessed 19 carbons including one methyl, five methylenes, eight methines, and five nonprotonated carbons. The NMR data demonstrated that **2** was an eburnean-type alkaloid similar as meloyunine [14], with exception of an additional methine (*δ*_H_ 3.92; *δ*_C_ 54.9). The abundance ration of 329.1414/331.1388 (3:1) in HRESIMS spectrum indicated the presence of a chlorine atom in **2** (Figure S16, Supporting Information). HMBC correlation of H-14 (*δ*_H_ 3.92) to C-20 (*δ*_*C*_ 44.3) established that the chlorine atom was located at C-14. Finally, the planar structure and relative configuration of **2** was established by 2D NMR spectra (Fig. 2), and its absolute configuration was determined by X-ray single crystal diffraction analysis with Flack parameter = 0.104 (12) (Fig. 5).

Bousmekine C (**3**) had a molecular formula of C_19_H_24_N_2_O_2_, as established by HRESIMS ion at *m/z* 313.1912 [M + H]^+^ (calcd 313.1911), corresponding to nine degrees of unsaturation. Detailed analysis of its NMR spectra demonstrated that **3** was structurally related to eburnamine [15]. The deshielded of C-3 (*δ*_C_ 58.7, Δ*δ* + 13.7), C-5 (*δ*_C_ 70.5, Δ*δ* + 18.9), and C-21 (*δ*_C_ 58.7, Δ*δ* + 13.2) in **3** inferred that **3** was the *N*-4-oxide derivative of eburnamine [16]. 2D NMR spectra (HSQC, HMBC, ^1^H-^1^H COSY, and ROESY) further confirmed the structure of **3** (Fig. 2). The ROESY correlations of H-16/H-19a, H-19a/H-21 indicated these protons took the same orientation and thus established the relative configuration of (16R*, 20S*, 21S*)-**3**. Finally, the absolute configuration of (16*R*, 20*S*, 21*S*)-**3** was assigned by the negative Cotton effects at 220 nm [15].

Bousmekine D (**4**) had the same molecular formula with **3**, as established by HRESIMS analysis at *m/z* 313.1908 [M + H]^+^ (calcd 313.1911). Compared its NMR data with **3** indicated that both compounds had the same skeleton. The coupling constant of H-16 (br d, *J* = 4.8 Hz) demonstrated that H-16 took *β* orientation and thus compound **4** was assigned as the C-16 epimer of **3**. Further 2D NMR spectra (HSQC, HMBC,^1^H-^1^H COSY, and ROESY) and ECD data analysis confirmed the structure of **4**, as shown in Fig. 1.

Bousmekine E (**5**) was obtained as an optically active colorless solid, [*α*]_D_^20^ − 66 (*c* 0.1, MeOH). Its molecular formula, C_10_H_13_NO_2_, was established by HRESIMS analysis at *m/z* 180.1018 [M + H]^+^ (calcd 180.1019), corresponding to five degrees of unsaturation. The ^13^C and DEPT spectra showed that **5** comprises 10 carbons, including two methyls, one methylene, five methines and two nonprotonated carbons.

The ^1^H-^1^H COSY spectrum gave three structural fragments: **a** (C-4 to C-11), **b** (C-6 to C-7), and **c** (C-9 to C-10). HMBC correlations of H-6 (*δ*_H_ 4.78, dd, *J* = 8.4, 3.0 Hz) to C-4 (*δ*_C_ 70.7), and of H-11 (*δ*_H_ 1.59, d, *J* = 6.6) and H-7a (*δ*_H_ 2.78, dd, *J* = 16.7, 8.4 Hz) to C-3 (*δ*_C_ 135.9) established the linkage of **a** and **b**, which formed a pyran moiety. Meanwhile, HMBC correlations of H-2 (*δ*_H_ 8.40, s) to C-4, C-8 (*δ*_C_ 142.3), and C-10 (*δ*_C_ 148.4), and of H-9 (*δ*_H_ 7.10, d, *J* = 4.8 Hz) to C-3 and C-7 (*δ*_C_ 34.7) finally established the connectivities of **b**, **c**, and the nitrogen atom. Thus, the planar structure of **5** was finally established as a novel pyranopyridine alkaloid (Fig. 2).

The ROESY correlation of H-4 and H-6 indicated that both protons took the same orientation and were arbitrarily designated as *α*-orientation, thus its relative configuration was determined as (4S*,6S*)-**5** (Fig. 3). To allocate the absolute configuration of **5**, the optical rotation (OR) value of (4*S*,6*S*)-**5** was calculated by using density functional theory (DFT) method. The calculated [*α*] value for (4*S*,6*S*)-**5** was -67, which is close to its experimental value (–66), and thus the absolute configuration of **5** was assigned as (4*S*,6*S*).

### Cytotoxic Activity

Five new compounds were evaluated for their cytotoxicities against five human cancer cell lines: HL-60, SW-480, A549, MCF-7, and SMMC-7721 using the MTT method with cisplatin (DDP) and paclitaxel (PXL) as positive controls [17]. Among them, only compound **1** exhibited significant cytotoxicity with IC_50_ values of 0.8–7.4 μM (Table 3).

**Table 3:** Cytotoxicity of compound **1** (IC_50_^*a*^, *μ*M)

Compounds	HL-60	SW480	SMMC-7721	A549	MCF-7
**1**	0.8 ± 0.1	0.8 ± 0.1	1.1 ± 0.1	5.1 ± 0.2	7.4 ± 0.4
DDP^*b*^	4.8 ± 0.2	15.4 ± 0.7	14.1 ± 0.9	17.9 ± 0.4	25.9 ± 0.2
PXL^*b*^	< 0.008	< 0.008	0.1 ± 0.0	< 0.008	< 0.008

^*a*^IC_50_: 50% inhibitory concentration

^*b*^Positive control (*μ*M)

## Experimental

### General Experimental Procedures

NMR spectra were measured via Bruker AV-500 and Avance III 600 MHz, TMS was used as an internal standard. IR spectra were surveyed on a Bio-Rad FTS-135 with KBr pellets. A JASCO P-1020 digital polarimeter was used to get optical rotations, while the ECD spectral data were measured by an Applied Photophysics Chirascan Spectrometer. HRESIMS and ESI were surveyed on Aglient 1290 UPLC/6540 Q-TOF spectrometer. Silica gel (80–100 and 100–200 mesh, Qingdao Marine Chemical Inc., China), silica gel H (10–40 μm, Qingdao Puke Chemical Inc., China), and Sephadex LH-20 (40–70 μm, Amersham Pharmacia Biotech AB), were used for column chromatography. Semi-preparative HPLC was carried out using a Shimadzu LC-20AT liquid chromatograph equipped with a YMC Triart C18 ExRS (5 μm; 10 × 250 mm) reversed-phase column.

### Plant Material

The twigs and leaves of *B. angustifolia* and *B. mekongensis* were collected in Xishuangbanna, Yunnan Province, People’s Republic of China, in July 2018. The samples were identified by Mr. Yu Chen, Kunming Botanical Garden. A specimen (no. ZY20180623 and no. ZY20180624) was deposited at State Key Laboratory of Phytochemistry and Plant Resource in West China, Kunming Institute of Botany, Chinese Academy of Science (CAS).

### Extraction and Isolation

The dried twigs and leaves of *B. mekongensis* (30.5 kg) were powdered and extracted three times with methanol. The extract was adjusted to 2–3 with hydrochloric acid (5%) and then extracted three times with petroleum ether. The water fraction was basified to pH 9–10 with sodium hydroxide (10%), then extracted with chloroform to get the crude alkaloids. The crude alkaloids (530 g) were separated on a silica gel column (100–200 mesh), and eluted with a gradient of CHCl_3_-MeOH (40:1 → 1:1) to yield five fractions (A-E). Fraction D (19.1 g) was purified by a RP-18 column (MeOH/H_2_O, 50:50 → 100:0, v/v) to give four subfractions (DI-DIV). Subfraction DII (5.2 g) was purified by a RP-18 column (MeOH/H_2_O, 50:50 → 100:0, v/v) and followed by semipreparative HPLC with MeOH/H_2_O (75:25, 0.1% Et_2_NH) to give **1** (8.0 mg, t_*R*_ 33.0 min). Subfraction DIV (4.9 g) was purified by a RP-18 column (MeOH/H_2_O, 50:50 → 100:0, v/v) and followed by semipreparative HPLC with MeOH/H_2_O (80:20, 0.1% Et_2_NH) to afford **2** (12.0 mg, t_*R*_ 23.5 min).

The dried twigs and leaves of *B. angustifolia* (57.5 kg) were powdered and extracted three times with methanol. The crude alkaloids (730 g) were obtained according to the above method and were separated on a silica gel column (100–200 mesh) by CHCl_3_-MeOH (40:1 → 1:1) to yield 6 fractions (A-F). Fraction D (30.0 g) was purified by a RP-18 column (MeOH/H_2_O, 40:60 → 100:0, v/v) to give four subfractions (DI-DV). Subfraction DII (8.2 g) was purified by a RP-18 column (MeOH/H_2_O, 50:50 → 100:0, v/v) and followed by semipreparative HPLC with MeOH/H_2_O (50:50, 0.1% Et_2_NH) to give **3** (14.4 mg, t_*R*_ 24.0 min) and **4** (2.5 mg, t_*R*_ 32.0 min). Subfraction DIV (6.9 g) was purified by a RP-18 column (MeOH/H_2_O, 50:50 → 100:0, v/v) and followed by semipreparative HPLC with MeOH/H_2_O (62:38, 0.1% Et_2_NH) to afford **5** (2.4 mg, t_*R*_ 28.5 min).

### Bousmekine A (**1**)

Bousmekine A (**1**): colorless solid; [α]_D_^20^ − 22 (*c* 0.1, MeOH); UV (MeOH) *λ*_max_ (log *ε*): 327 (3.91) nm; ECD (0.0006 M, MeOH) λ_max_ (∆*ε*) 210 (+ 5.33), 226 (+ 2.64) 241 (+ 4.63), 320 (− 3.43); IR (KBr) *v*_max_ 3441, 2963, 1676, 1617, 1500, 1438, 1263, 1113 cm^−1^; ^1^H and ^13^C NMR data (CDCl_3_, 600 and 150 MHz) see Tables 1 and 2; HRESIMS *m/z* 423.2281 [M + H]^+^ (calcd for C_25_H_31_N_2_O_4_, 423.2278).

### Bousmekine B (**2**)

Bousmekine B (**2**): colorless crystal; [α]_D_^20^ − 98 (*c* 0.1, MeOH); UV (MeOH) *λ*_max_ (log *ε*): 230 (4.27), 272 (3.65) nm; ECD (0.0005 M, MeOH) λ_max_ (∆*ε*) 207 (+ 2.60), 231 (− 17.46); IR (KBr) *v*_max_ 3450, 2924, 1658, 1487, 1454, 1334, 1295, 1046 cm^−1^; ^1^H and ^13^C NMR data (CDCl_3_, 600 and 150 MHz) see Tables 1 and 2; HRESIMS *m/z* 329.1414 [M + H]^+^ (calcd for C_19_H_21_N_2_OCl, 329.1415).

### Bousmekine C (**3**)

Bousmekine C (**3**): colorless solid; [α]_D_^20^ − 45 (*c* 0.1, MeOH); UV (MeOH) *λ*_max_ (log *ε*): 223 (3.90) nm; ECD (0.0008 M, MeOH) λ_max_ (∆*ε*) 200 (+ 1.15), 221 (− 1.47), 234 (+ 1.24); IR (KBr) *v*_max_ 3450, 2923, 1658, 1632, 1380, 1326, 1296 1011 cm^−1^; ^1^H and ^13^C NMR data (CD_3_OD, 600 and 150 MHz) see Tables 1 and 2; HRESIMS *m/z* 313.1912 [M + H]^+^ (calcd for C_19_H_25_N_2_O_2_, 313.1911).

### Bousmekine D (**4**)

Bousmekine D (**4**): colorless solid; [α]_D_^20^ + 18 (*c* 0.1, MeOH); UV (MeOH) λ_max_ (log *ε*): 224 (4.33) nm; ECD (0.0004 M, MeOH) λ_max_ (∆*ε*) 223 (+ 9.72); IR (KBr) *v*_max_ 3418, 2922, 2852, 1724, 1659, 1459, 1384, 1207, 1056 cm^−1^; ^1^H and ^13^C NMR data (CD_3_OD, 600 and 150 MHz) see Tables 1 and 2; HRESIMS *m/z* 313.1908 [M + H]^+^ (calcd for C_19_H_25_N_2_O_2_, 313.1911).

### Bousmekine E (**5**)

Bousmekine E (**5**): colorless solid; [α]_D_^20^ − 66 (*c* 0.1, MeOH); UV (MeOH) λ_max_ (log *ε*): 259 (3.08) nm; IR (KBr) *v*_max_ 3442, 2918, 1736, 1647, 1542, 1467, 1384, 1261, 1048 cm^−1^; ^1^H and ^13^C NMR data (acetone–*d*_6_, 600 and 150 MHz) see Tables 1 and 2; HRESIMS *m/z* 180.1018 [M + H]^+^ (calcd for C_10_H_14_NO_2_, 180.1019).

### Crystal data for Bousmekine B (**2**)

Bousmekine B (**2**): 4(C_19_H_21_ClN_2_O)·C_2_H_6_O, *M* = 1361.37, *a* = 11.9505(3) Å, *b* = 12.3727(3) Å, *c* = 22.7041(6) Å, *α* = 90°, *β* = 95.5750(10)°, *γ* = 90°, *V* = 3341.15(15) Å^3^, *T* = 100.(2) K, space group *P*1211, *Z* = 2, *μ*(Cu Kα) = 2.091 mm^−1^, 73809 reflections measured, 13008 independent reflections (*R*_*int*_ = 0.1435). The final *R*_*1*_ values were 0.0675 (*I* > 2*σ*(*I*)). The final *wR*(*F*^2^) values were 0.1701 (*I* > 2*σ*(*I*)). The final *R*_*1*_ values were 0.0798 (all data). The final *wR*(*F*^2^) values were 0.1860 (all data). The goodness of fit on *F*^2^ was 1.030. Flack parameter = 0.104(12). Crystallographic data (excluding structure factor tables) for compound **2** have been deposited with the Cambridge Crystallographic Data Center as supplementary publication (deposit number CCDC 2026043). Copies of the data can be obtained free of charge by application to CCDC, 12 Union Road, Cambridge CB 1EZ, UK [fax: Int. + 44 (0) (1223) 336 033; e-mail: deposit@ccdc.cam.ac.uk.

### Cytotoxicity Assays

Cytotoxicity evaluations were performed according to the previously described protocol [10, 17].

## Concluding Remarks

In this investigation, four new MIAs (**1**–**4**) and one pyranopyridine alkaloid (**5**) were isolated from the twigs and leaves of two *Bousigonia* species: *B. angustifolia* and *B. mekongensis*. Among them, compound **2** was an eburnea-type MIAs and featured by a rare chlorine atom at C-14, while **5** was characterized by a novel pyranopyridine moiety. Moreover, compound **1** exhibited significant cytotoxicities against several human cancer cell lines with IC_50_ values of 0.8–7.4 μM. The findings enriched the diversity of secondary metabolites of the *Bousigonia* genus.

## Electronic supplementary material

Below is the link to the electronic supplementary material.

Electronic supplementary material 1 (PDF 4468 kb)

Electronic supplementary material 2 (CIF 2240 kb)
