# Supplementary material for: Bousmekines A-E, New Alkaloids from Two Bousigonia Species: B.angustifolia and B. mekongensis
Source: Nat Prod Bioprospect. 2020 Nov 3;11(2):207–13. doi: 10.1007/s13659-020-00278-6 (PMC7981358; doi:10.1007/s13659-020-00278-6)
Supplement: Supplementary file 1 — Electronic supplementary material 1 (PDF 4468 kb) [file 13659_2020_278_MOESM1_ESM.pdf]

Bousmekines A-E, new alkaloids from two *Bousigonia* species*: B. angustifolia and B. mekongensis*

Zong-Qing Huo, Qian Zhao, Wen-Tao Zhu, Xiao-Jiang Hao, and Yu Zhang*

State Key Laboratory of Phytochemistry and Plant Resources in West China, Kunming Institute of Botany, Chinese Academy of Sciences, Kunming 650201, PR China

*Corresponding author. Tel./fax: +86-871-65223263. *E-mail address*: zhangyu@mail.kib.ac.cn (Y. Zhang).

Supporting Information

Contents

1. Spectra of physico-chemical properties of **1-5**

Figure S1 ^{1}H NMR spectrum of bousmekine A (**1**) in $CDCl_3$

Figure S2 ^{13}C NMR spectrum of bousmekine A (**1**) in $CDCl_3$

Figure S3 HSQC spectrum of bousmekine A (**1**) in $CDCl_3$

Figure S4 ^{1}H-^{1}H COSY spectrum of bousmekine A (**1**) in $CDCl_3$

Figure S5 HMBC spectrum of bousmekine A (**1**) in $CDCl_3$

Figure S6 ROESY spectrum of bousmekine A (**1**) in $CDCl_3$

Figure S7 HRESIMS spectrums of bousmekine A (**1**)

Figure S8 IR spectrum of bousmekine A (**1**)

Figure S9 ECD spectrum of bousmekine A (**1**)

Figure S10 ^{1}H NMR spectrum of bousmekine B (**2**) in $CDCl_3$

Figure S11 ^{13}C NMR spectrum of bousmekine B (**2**) in $CDCl_3$

Figure S12 HSQC spectrum of bousmekine B (**2**) in $CDCl_3$

Figure S13 ^{1}H-^{1}H COSY spectrum of bousmekine B (**2**) in $CDCl_3$

Figure S14 HMBC spectrum of bousmekine B (**2**) in $CDCl_3$

Figure S15 ROESY spectrum of bousmekine B (**2**) in $CDCl_3$

Figure S16 HRESIMS spectrums of bousmekine B (**2**)

Figure S17 IR spectrum of bousmekine B (**2**)

Figure S18 ECD spectrum of bousmekine B (**2**)

Figure S19 X-ray crystal structure of bousmekine (**2)**

Figure S20 ^{1}H NMR spectrum of bousmekine C (**3**) in CD_3OD

Figure S21 ^{13}C NMR spectrum of bousmekine C (**3**) in CD_3OD

Figure S22 HSQC spectrum of bousmekine C (**3**) in CD_3OD

Figure S23 ^{1}H-^{1}H COSY spectrum of bousmekine C (**3**) in CD_3OD

Figure S24 HMBC spectrum of bousmekine C (**3**) in CD_3OD

Figure S25 ROESY spectrum of bousmekine C (**3**) in CD_3OD

Figure S26 HRESIMS spectrums of bousmekine C (**3**)

Figure S27 IR spectrum of bousmekine C (**3**)

Figure S28 ECD spectrum of bousmekine C (**3**)

Figure S29^{1}H NMR spectrum of bousmekine D (**4**) in CD_3OD

Figure S30 ^{13}C NMR spectrum of bousmekine D (**4**) in CD_3OD

Figure S31 HSQC spectrum of bousmekine D (**4**) in CD_3OD

Figure S32 ^{1}H-^{1}H COSY spectrum of bousmekine D (**4**) in CD_3OD

Figure S33 HMBC spectrum of bousmekine D (**4**) in CD_3OD

Figure S34 ROESY spectrum of bousmekine D (**4**) in CD_3OD

Figure S35 HRESIMS spectrums of bousmekine D (**4**)

Figure S36 IR spectrum of bousmekine D (**4**)

Figure S37 ECD spectrum of bousmekine D (**4**)

Figure S38 ^{1}H NMR spectrum of bousmekine E (**5**) in CD_3COCD_3
Figure S39 ^{13}C NMR spectrum of bousmekine E (**5**) in CD_3COCD_3
Figure S40 HSQC spectrum of bousmekine E (**5**) in CD_3COCD_3
Figure S41 ^{1}H-^{1}H COSY spectrum of bousmekine E (**5**) in CD_3COCD_3
Figure S42 HMBC spectrum of bousmekine E (**5**) in CD_3COCD_3
Figure S43 ROESY spectrum of bousmekine E (**5**) in CD_3COCD_3
Figure S44 HRESIMS spectrums of bousmekine E (**5**)
Figure S45 IR spectrum of bousmekine E (**5**)
2. Computational methods for ECD calculation of **1**
3. Computational method for [α] calculation of **5**

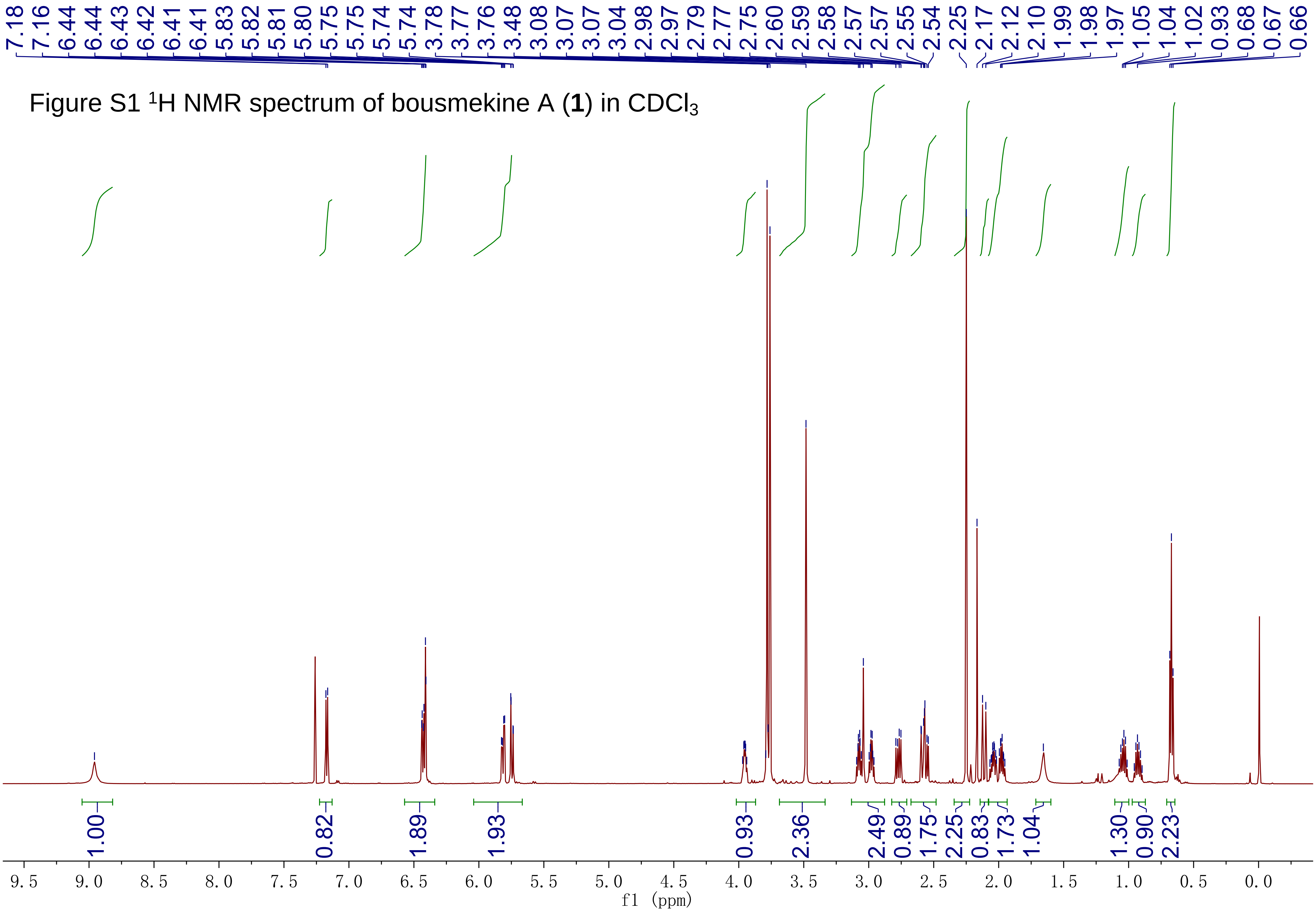

Figure S1 ^{1}H NMR spectrum of bousmekine A (**1**) in $CDCl_3$

Figure S2 ^{13}C NMR spectrum of bousmekine A (**1**) in $CDCl_3$

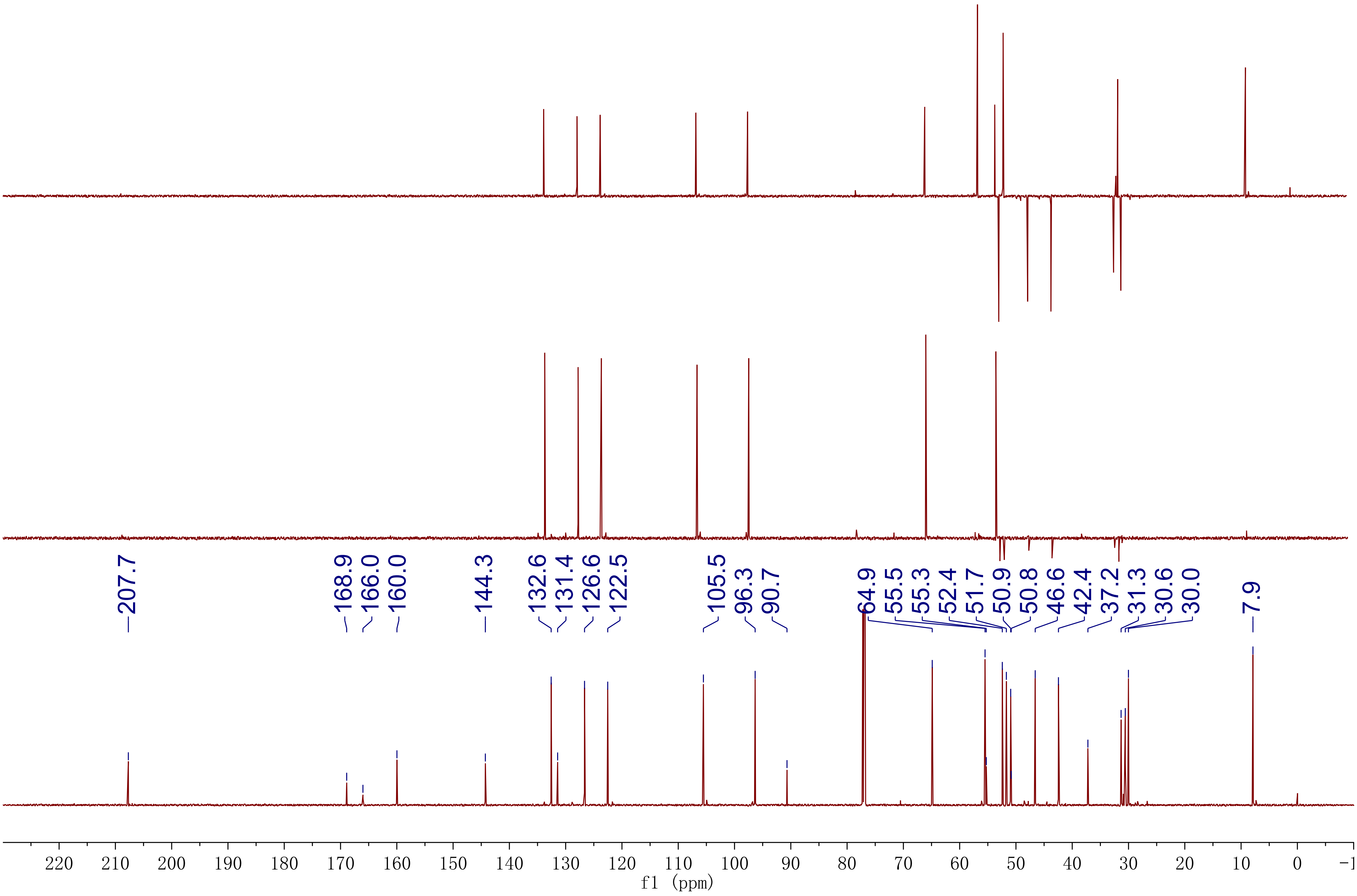

Figure S3 HSQC spectrum of bousmekine A (**1**) in $CDCl_3$

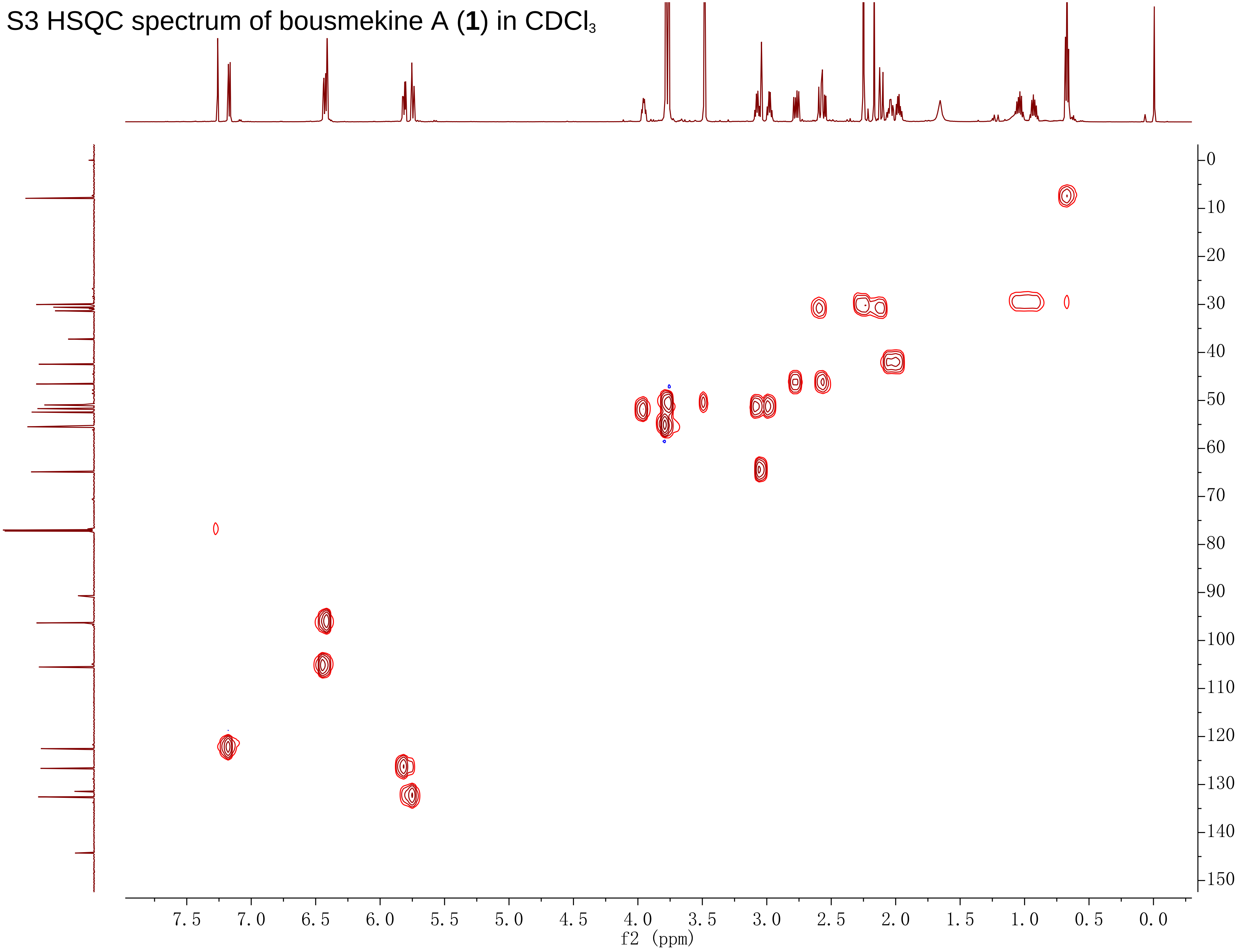

Figure S4 ^{1}H-^{1}H COSY spectrum of bousmekine A (**1**) in $CDCl_3$

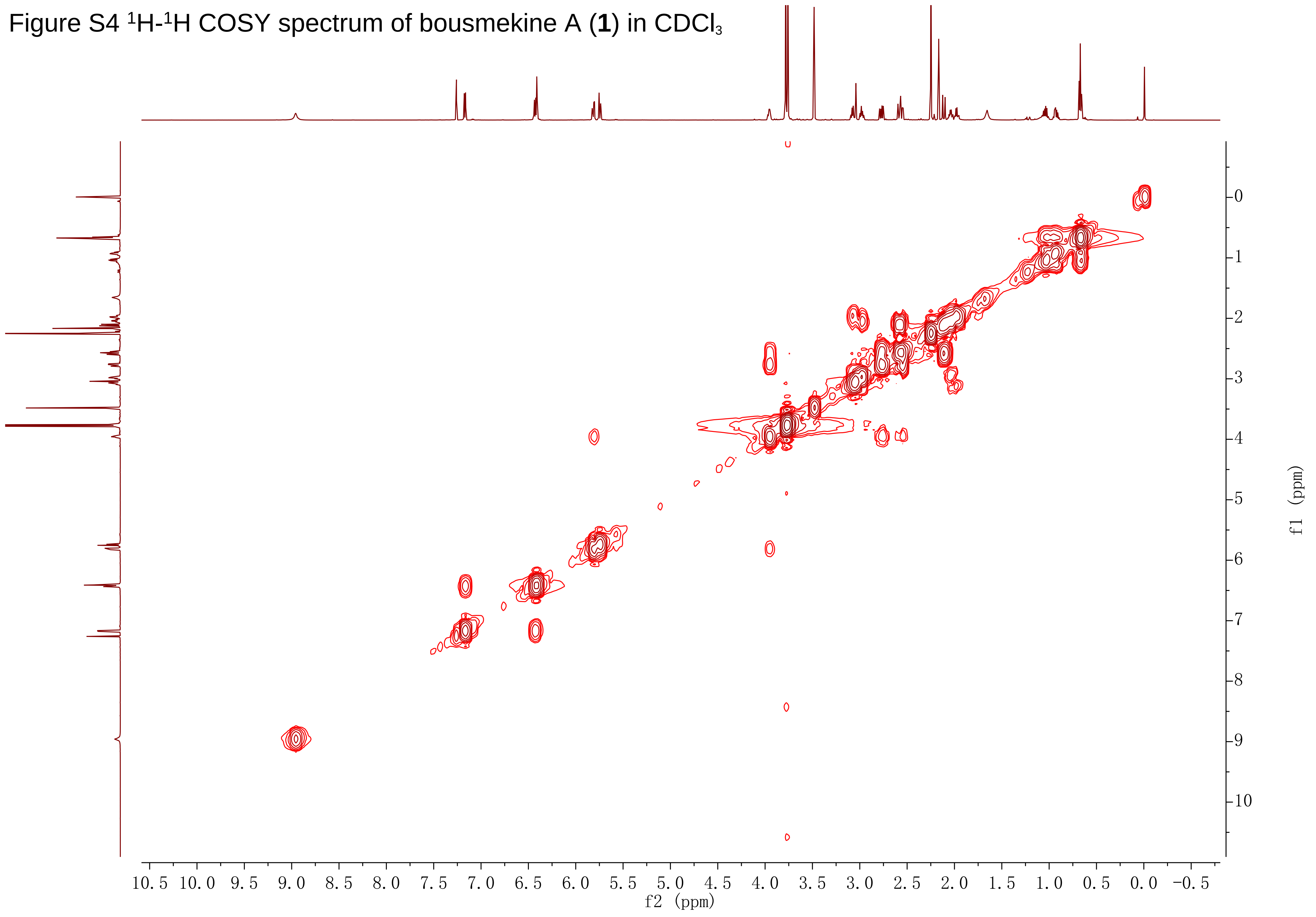

Figure S5 HMBC spectrum of bousmekine A (**1**) in $CDCl_3$

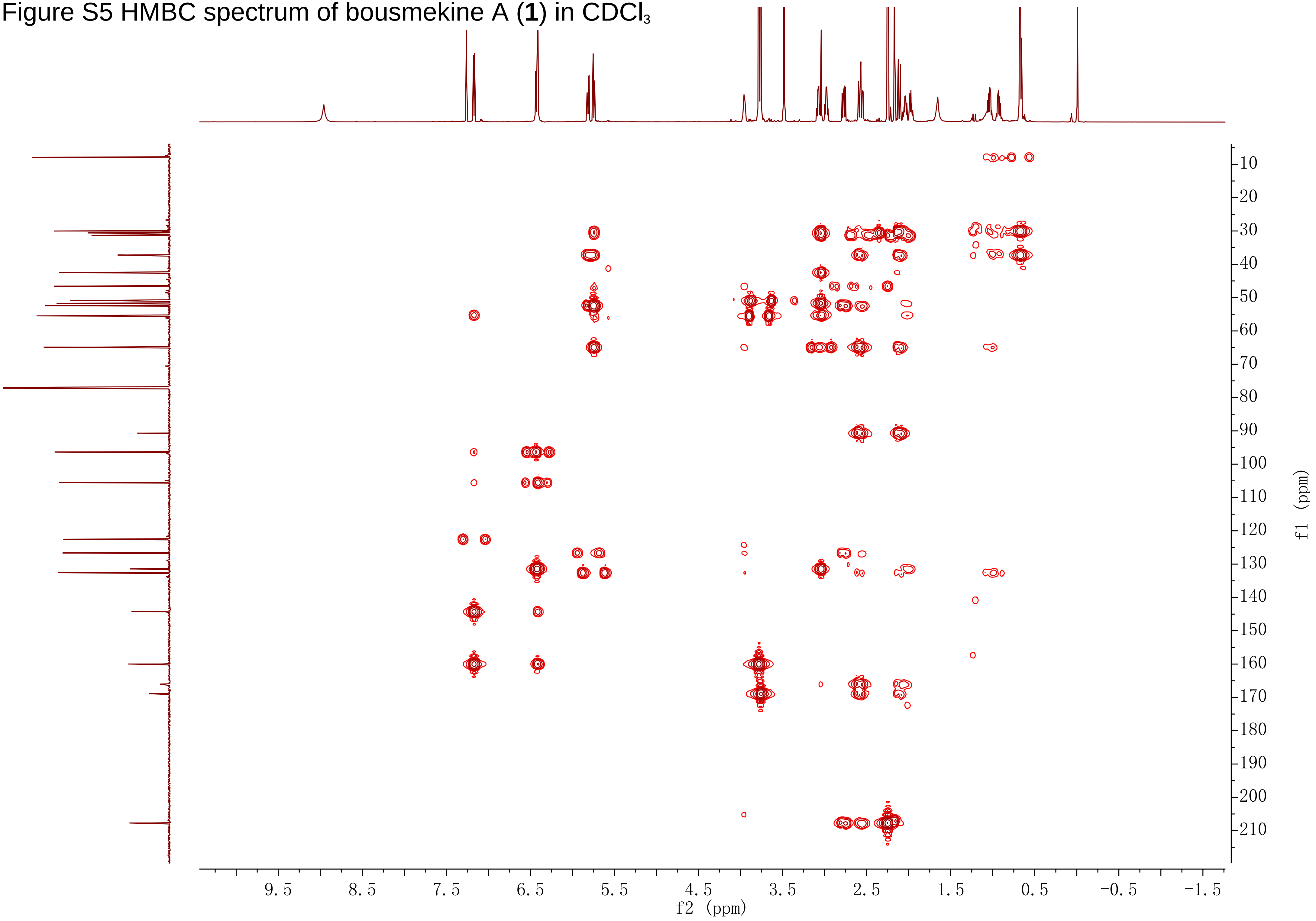

Figure S6 ROESY spectrum of bousmekine A (**1**) in $CDCl_3$

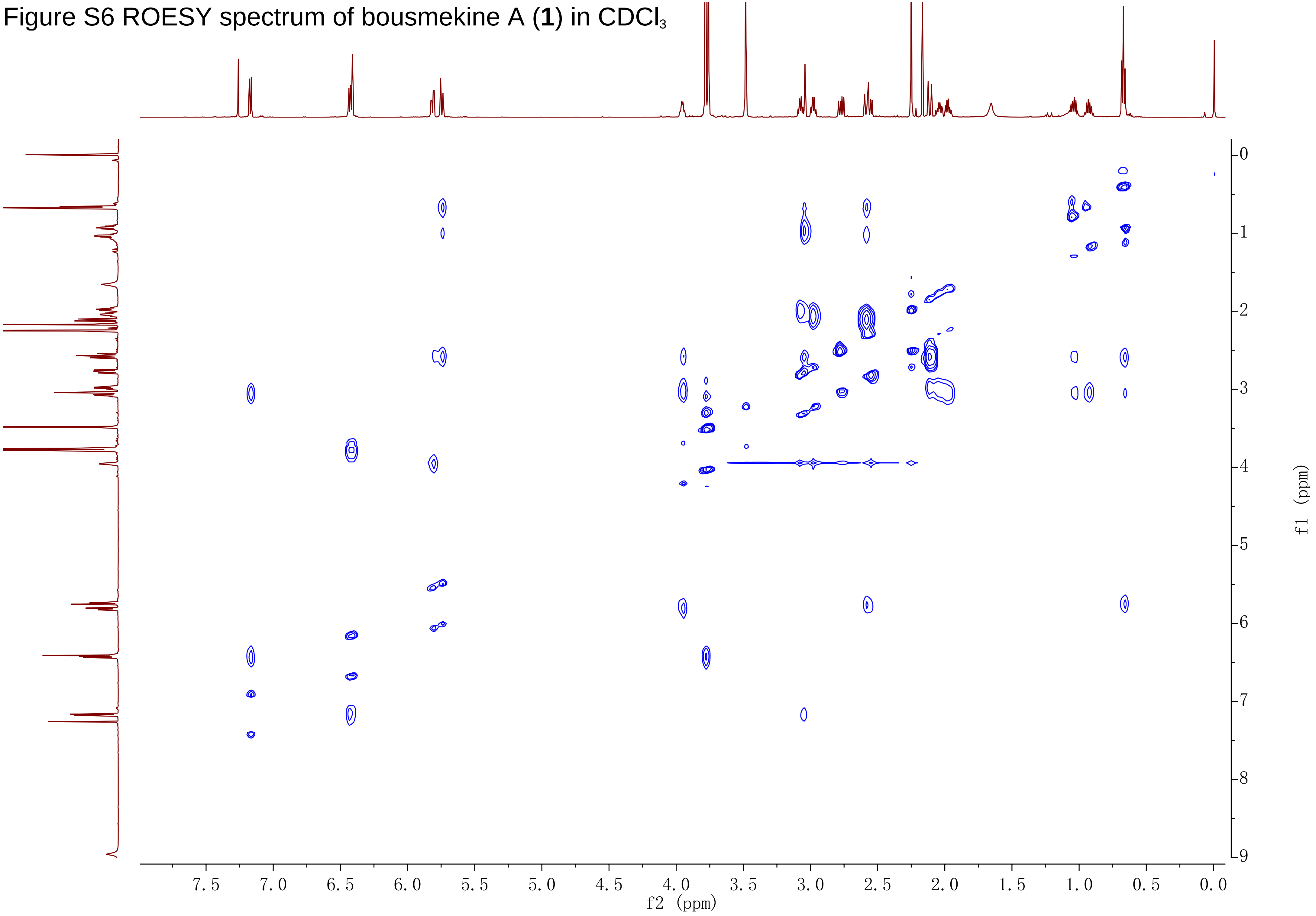

Figure S7 HRESIMS spectrums of bousmekine A (**1**)

Qualitative Analysis Report

**Data Filename**	HBM-18b.d	**Sample Name**	HBM-18b
**Sample Type**	Sample	**Position**	P1-A6
**Instrument Name**	Instrument 1	**User Name**	
**Acq Method**	s.m	**Acquired Time**	7/8/2020 2:48:31 PM
**IRM Calibration Status**	Success	**DA Method**	Default.m
**Comment**			

**Sample Group**		**Info.**
**Acquisition SW**	6200 series TOF/6500 series	
**Version**	Q-TOF B.05.01 (B5125.2)	

User Spectra

**Fragmentor Voltage**	**Collision Energy**	**Ionization Mode**
135	0	ESI

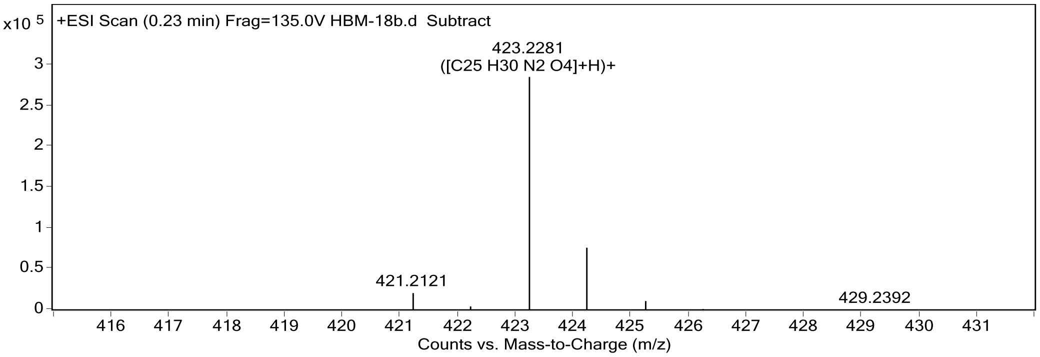

Peak List

m/z	z	Abund	Formula	Ion
367.202	1	12527.81		
421.2121	1	21170.9		
423.2281	1	285043.13	C25 H30 N2 O4	(M+H)+
424.2311	1	75900.75	C25 H30 N2 O4	(M+H)+
425.2346	1	11052.58	C25 H30 N2 O4	(M+H)+
437.2068	1	27426.22		
439.2232	1	27582.9		
745.3601	1	25192.94		
746.362	1	11636.48		
759.3755	1	15304.51		

Formula Calculator Element Limits

Element	Min	Max
C	3	60
H	0	120
O	0	30
N	0	10

Formula Calculator Results

Formula	CalculatedMass	CalculatedMz	Mz	Diff. (mDa)	Diff. (ppm)	DBE
C25 H30 N2 O4	422.2206	423.2278	423.2281	-0.30	-0.71	12.0000

--- End Of Report ---

Figure S8 IR spectrum of bousmekine A (**1**)

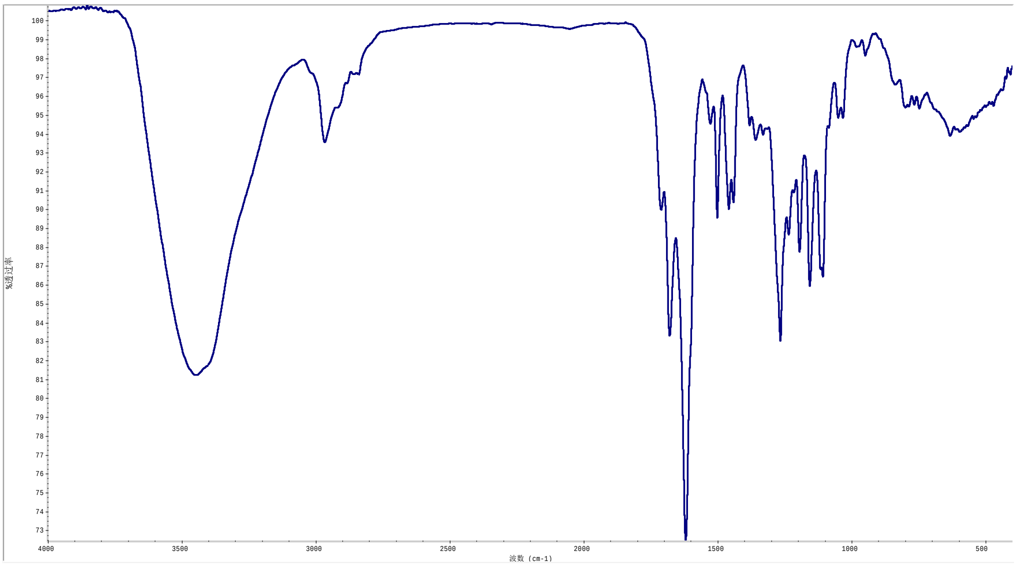

Sample Name: HBM-18b
KBr压片
采集时间: 星期五 7月 17 10:58:41 2020 (GMT+08:00)
仪器型号：NICOLET iS10
Software version: OMNIC 9.8.372

样品扫描次数: 16
背景扫描次数: 16
分辨率: 4.000
采样增益: 1.0
动镜速度: 0.4747
光阑: 80.00

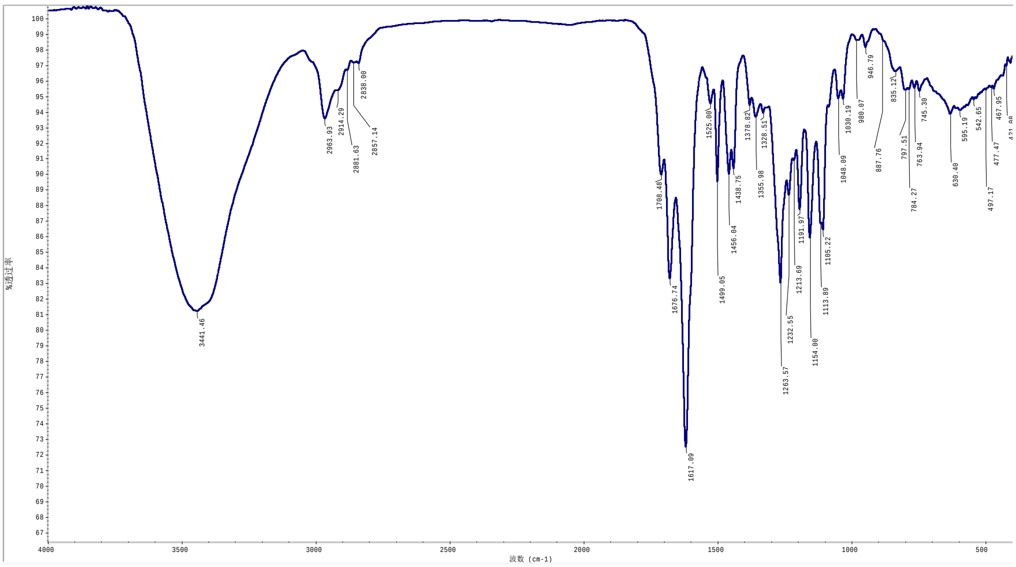

Sample Name: HBM-18b
KBr压片
采集时间: 星期五 7月 17 10:58:41 2020 (GMT+08:00)
仪器型号：NICOLET iS10
Software version: OMNIC 9.8.372

样品扫描次数: 16
背景扫描次数: 16
分辨率: 4.000
采样增益: 1.0
动镜速度: 0.4747
光阑: 80.00

Figure S9 ECD spectrum of bousmekine A (**1**)

HBM-18B

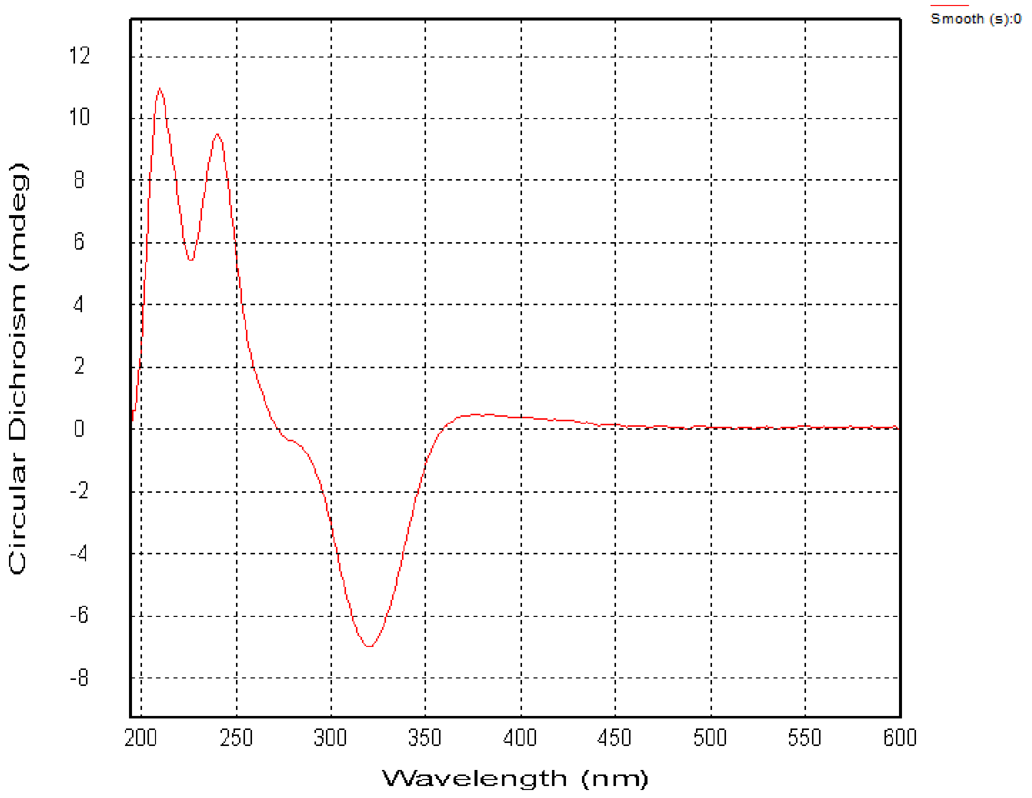

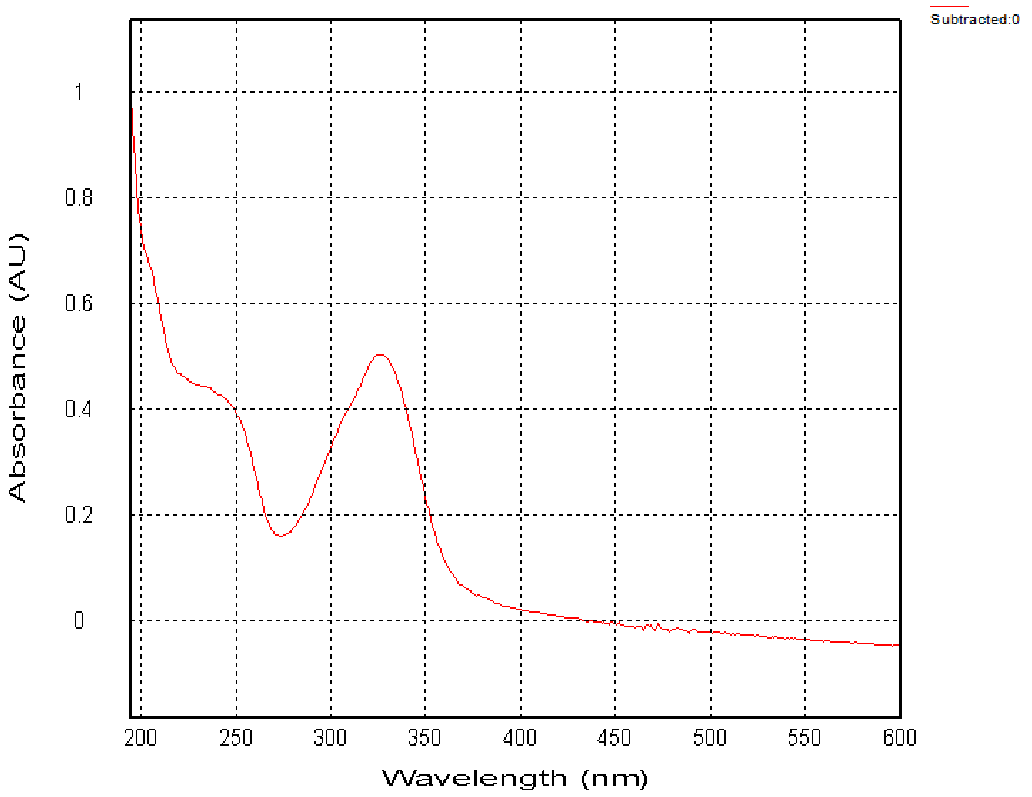

Figure S10 ^{1}H NMR spectrum of bousmekine B (**2**) in $CDCl_3$

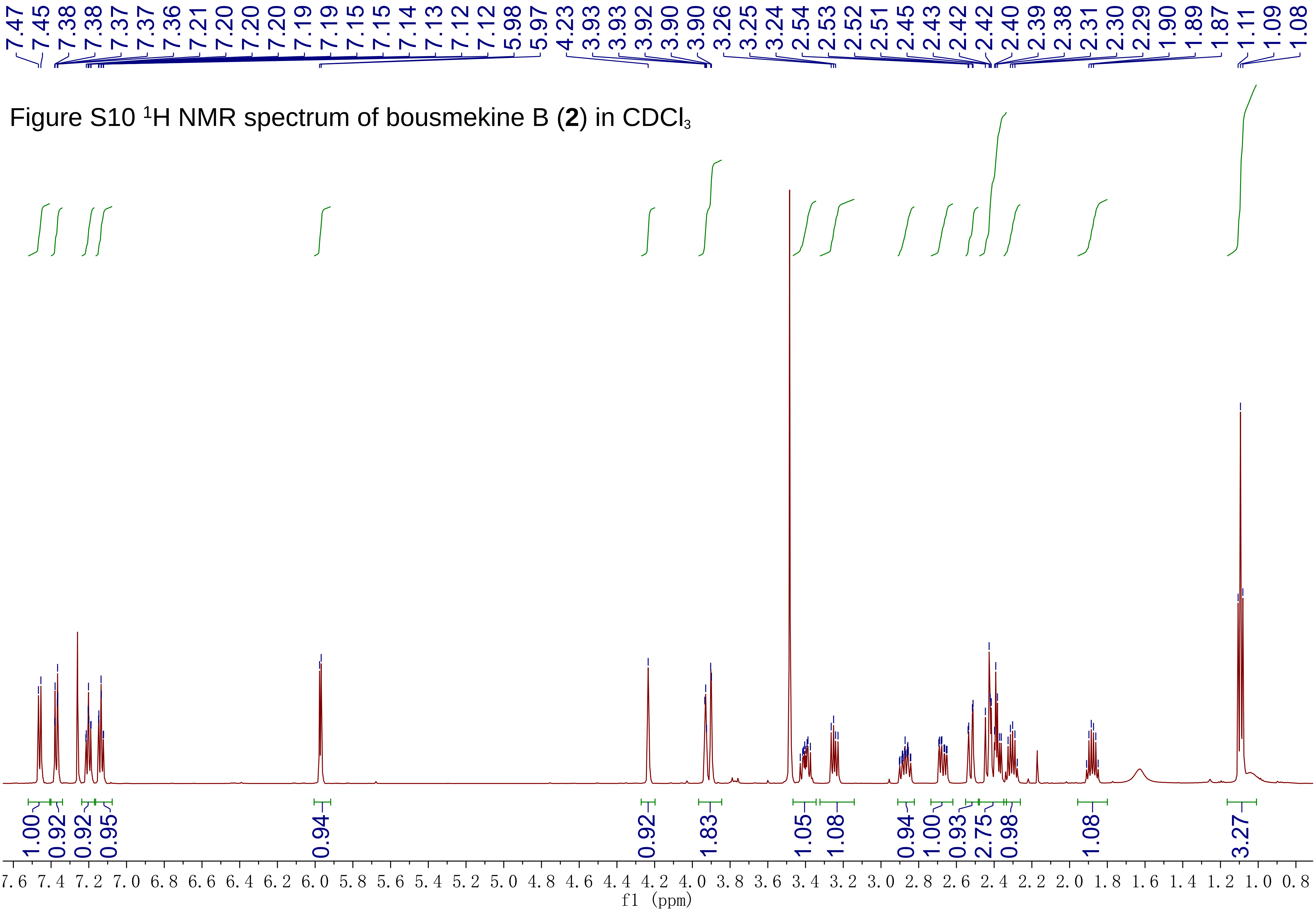

Figure S11 ^{13}C NMR spectrum of bousmekine B (**2**) in $CDCl_3$

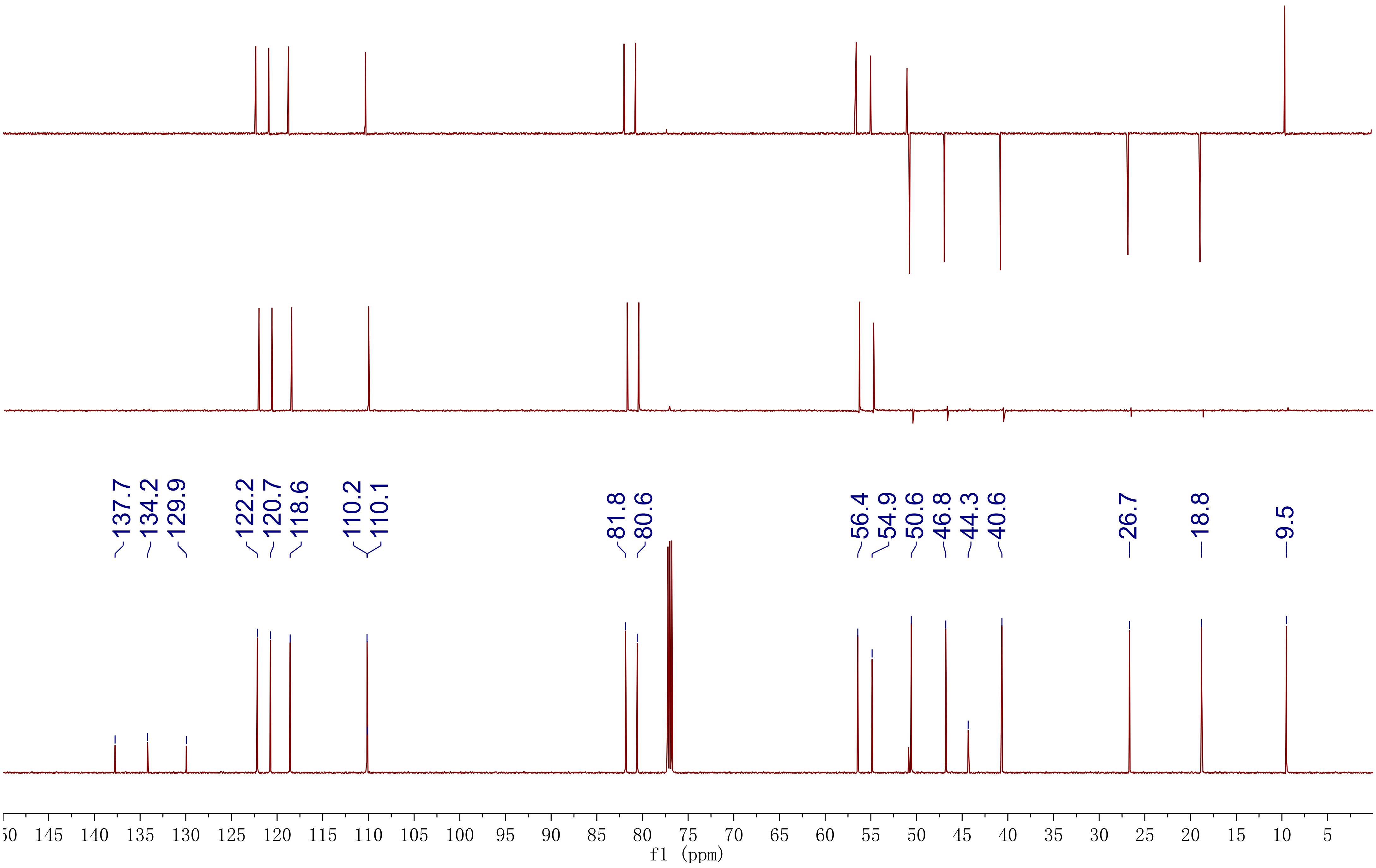

Figure S12 HSQC spectrum of bousmekine B (**2**) in $CDCl_3$

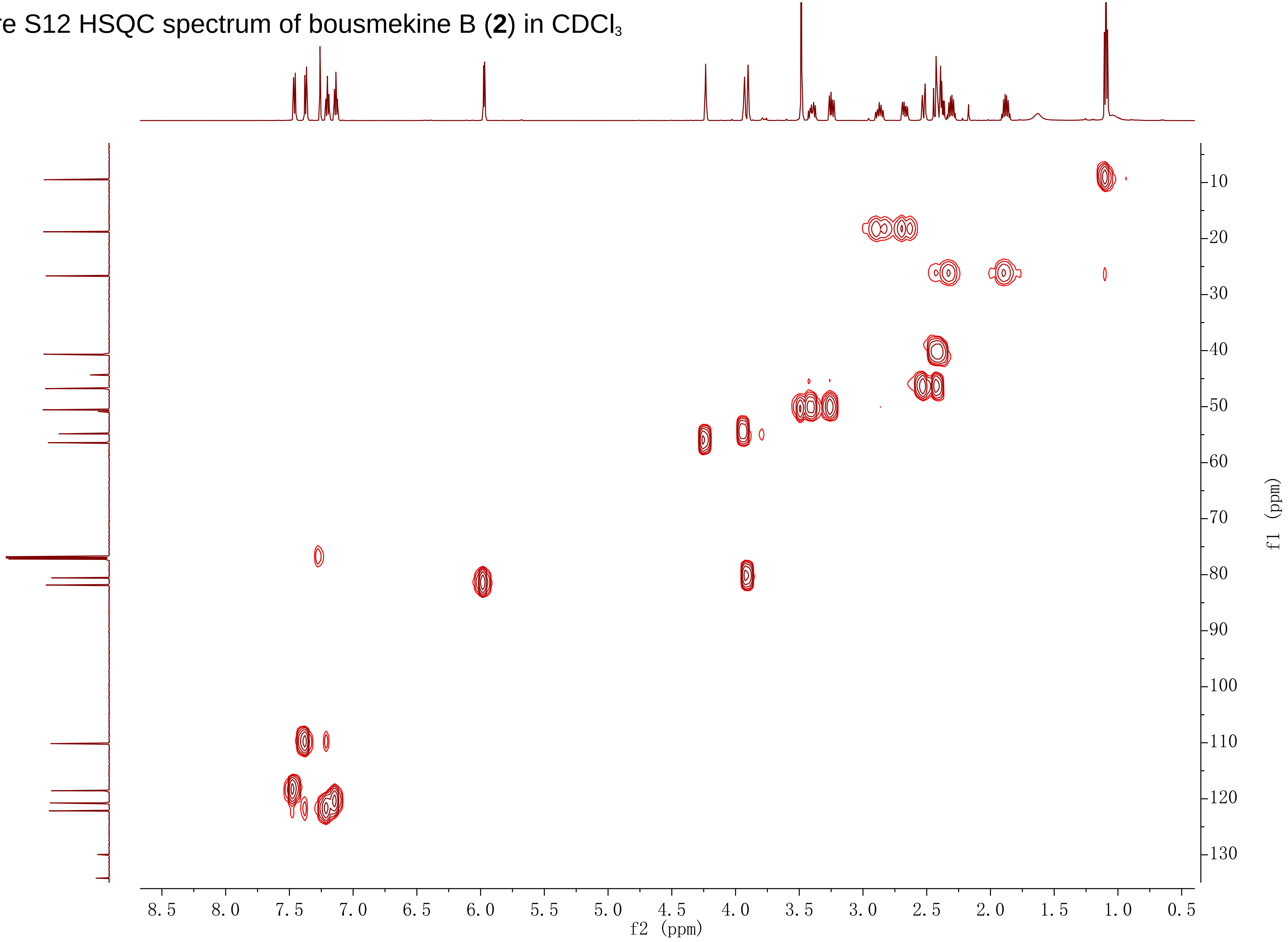

Figure S13 ^{1}H-^{1}H COSY spectrum of bousmekine B (**2**) in $CDCl_3$

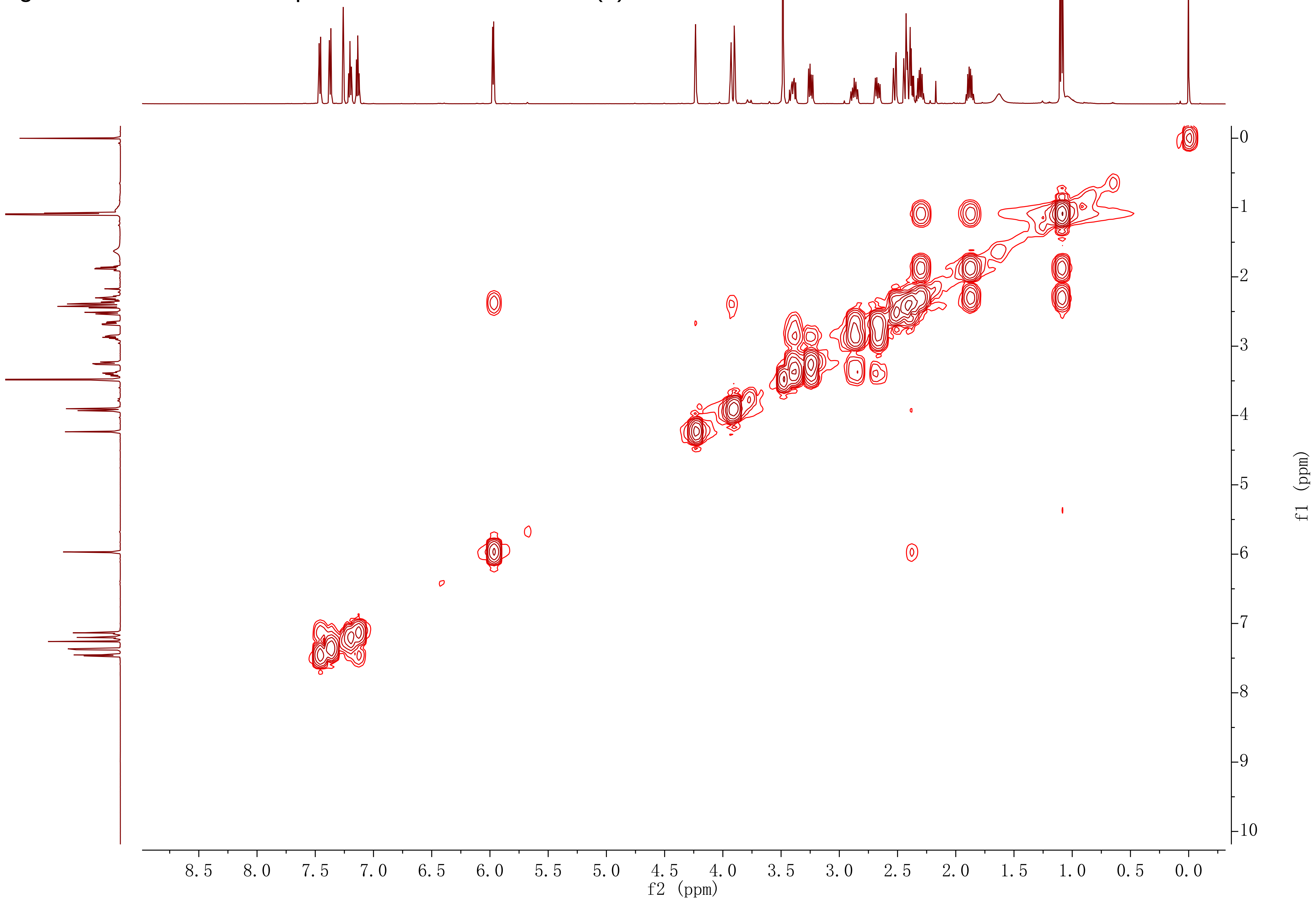

Figure S14 HMBC spectrum of bousmekine B (**2**) in $CDCl_3$

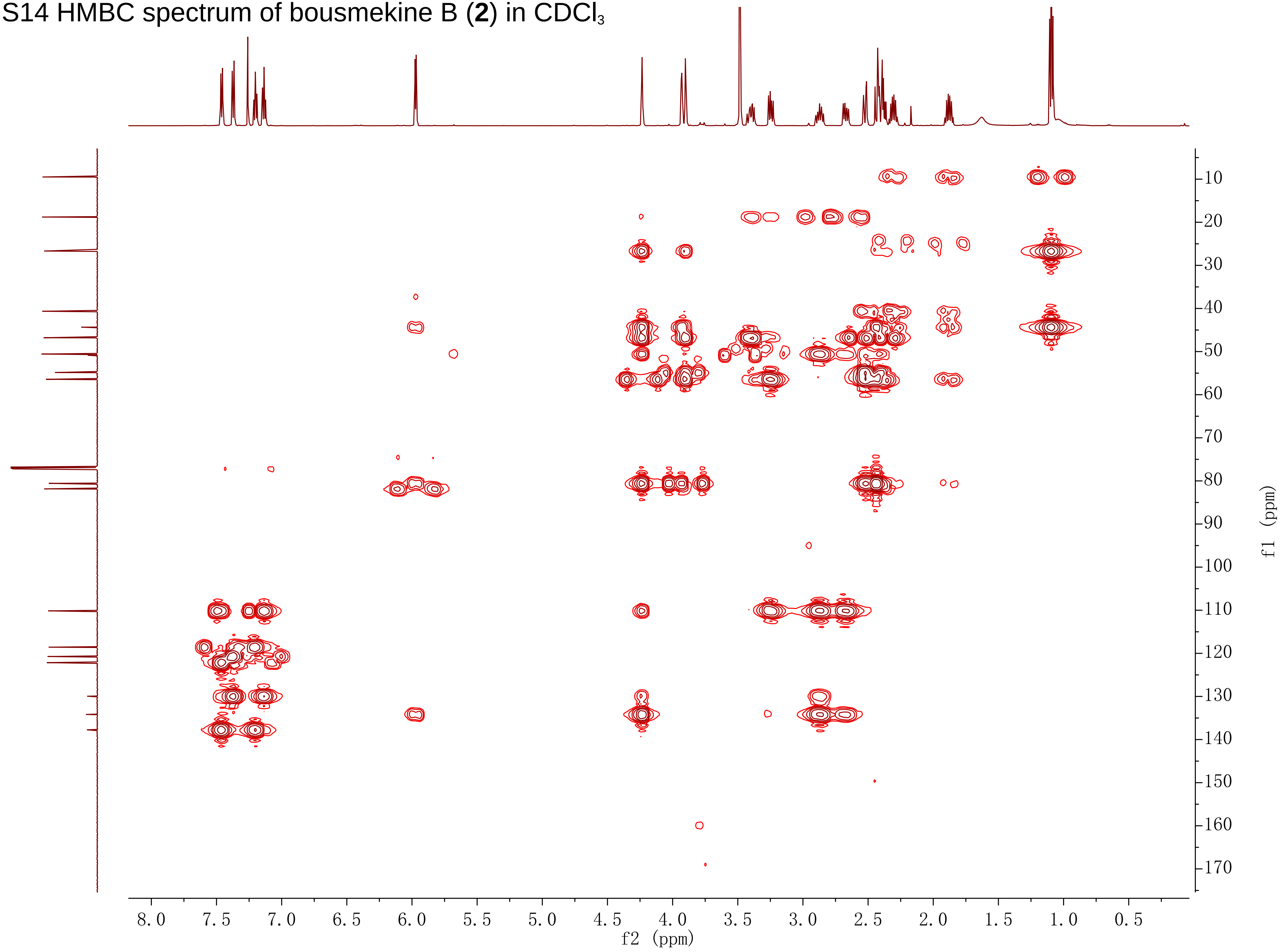

Figure S15 ROESY spectrum of bousmekine B (**2**) in $CDCl_3$

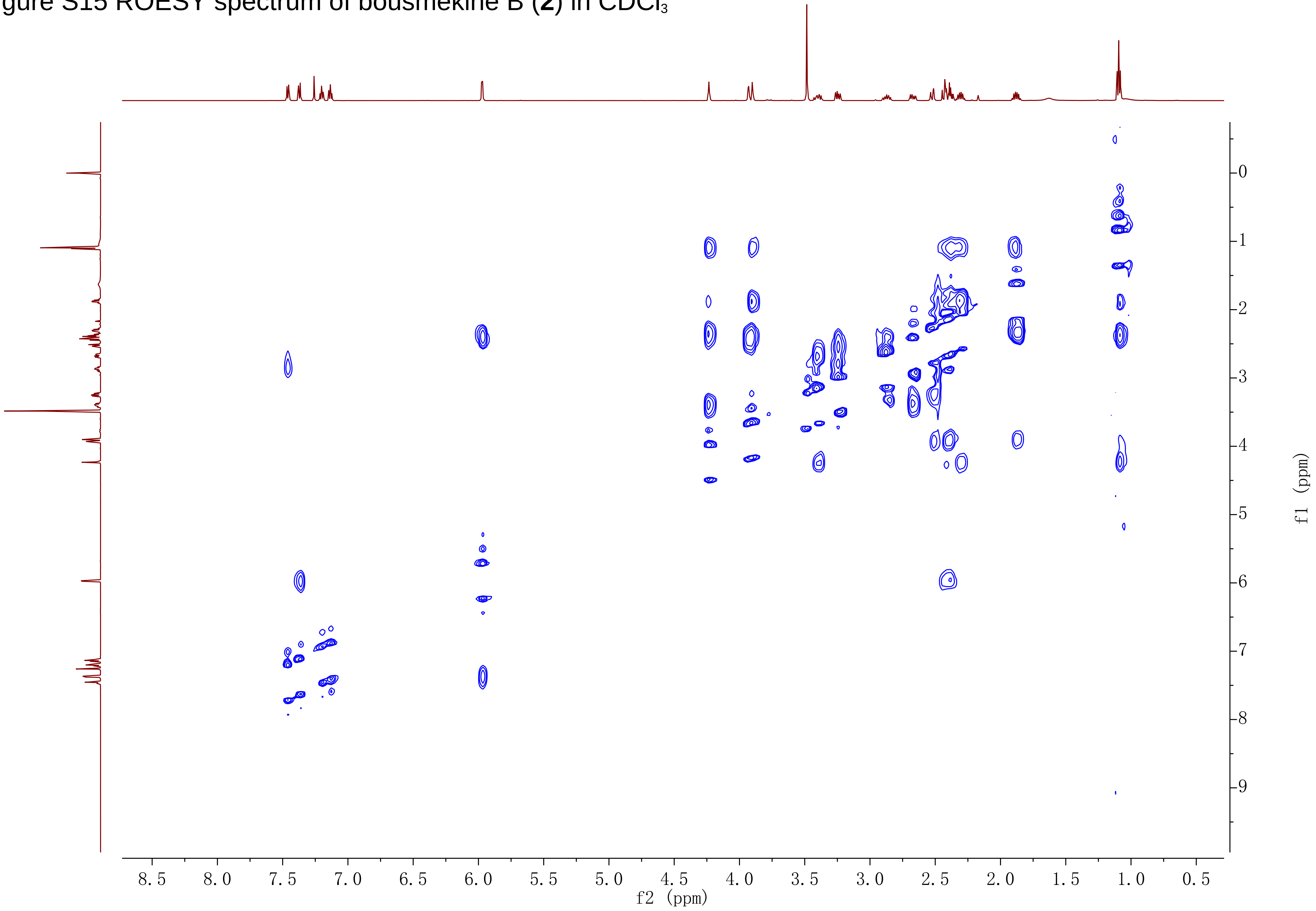

Figure S16 HRESIMS spectrums of bousmekine B (**2**)

Qualitative Analysis Report

**Data Filename**	HBM-18a.d	**Sample Name**	HBM-18a
**Sample Type**	Sample	**Position**	P1-A5
**Instrument Name**	Instrument 1	**User Name**	
**Acq Method**	s.m	**Acquired Time**	7/8/2020 2:47:19 PM
**IRM Calibration Status**	Success	**DA Method**	Default.m
**Comment**			

**Sample Group**		**Info.**
**Acquisition SW**	6200 series TOF/6500 series	
**Version**	Q-TOF B.05.01 (B5125.2)	

User Spectra

Fragmentor Voltage	Collision Energy	Ionization Mode
135	0	ESI

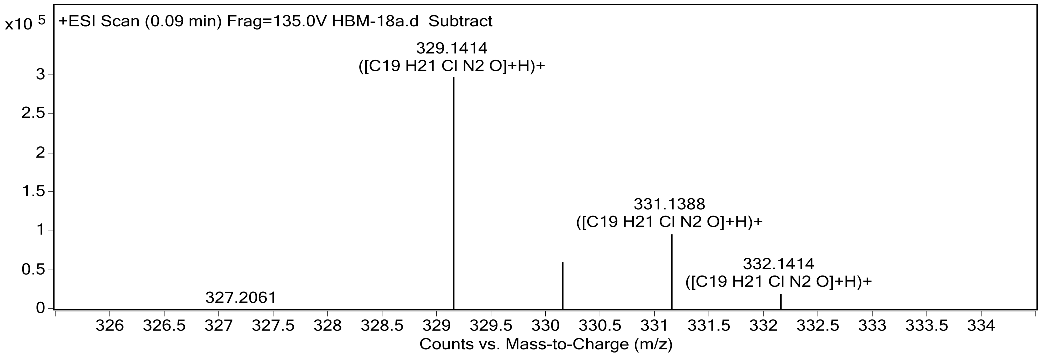

Peak List

m/z	z	Abund	Formula	Ion
173.0969		704.05		
222.127		836.19		
329.1414	1	297784.47	C19 H21 Cl N2 O	(M+H)+
330.1441	1	61339.79	C19 H21 Cl N2 O	(M+H)+
331.1388	1	97799.45	C19 H21 Cl N2 O	(M+H)+
332.1414	1	21104.07	C19 H21 Cl N2 O	(M+H)+
333.1451	1	1334.67	C19 H21 Cl N2 O	(M+H)+
345.1342		714.96		
361.1332		912.71		
421.2109		734.03		

Formula Calculator Element Limits

Element	Min	Max
C	3	60
H	0	120
O	0	30
N	0	5
Cl	0	3

Formula Calculator Results

Formula	CalculatedMass	CalculatedMz	Mz	Diff. (mDa)	Diff. (ppm)	DBE
C19 H21 Cl N2 O	328.1342	329.1415	329.1414	0.10	0.30	10.0000

--- End Of Report ---

Figure S17 IR spectrum of bousmekine B (**2**)

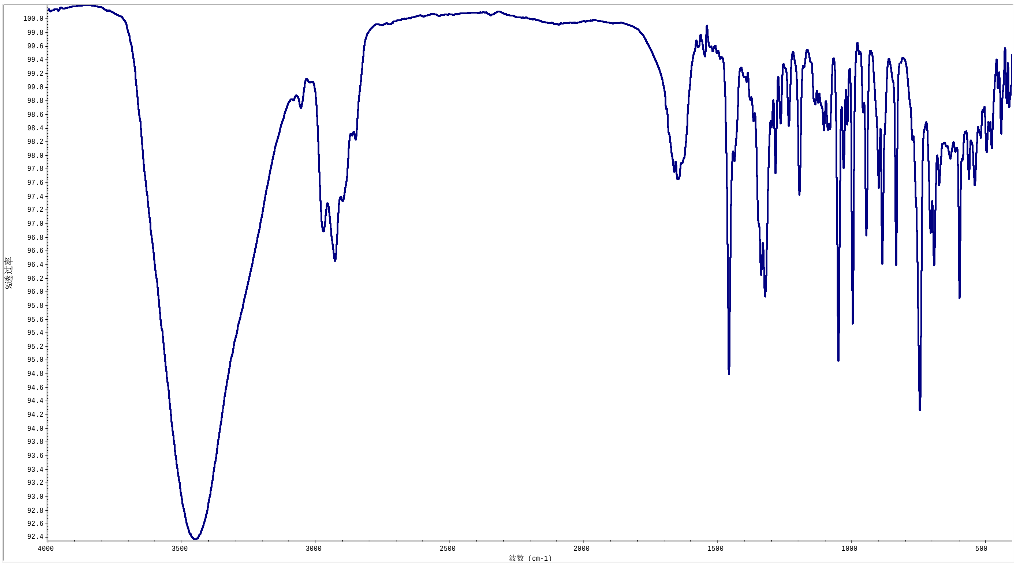

Sample Name: HBM-18a
KBr压片
采集时间: 星期五 7月 17 10:30:34 2020 (GMT+08:00)
仪器型号：NICOLET iS10
Software version: OMNIC 9.8.372

样品扫描次数: 16
背景扫描次数: 16
分辨率: 4.000
采样增益: 1.0
动镜速度: 0.4747
光阑: 80.00

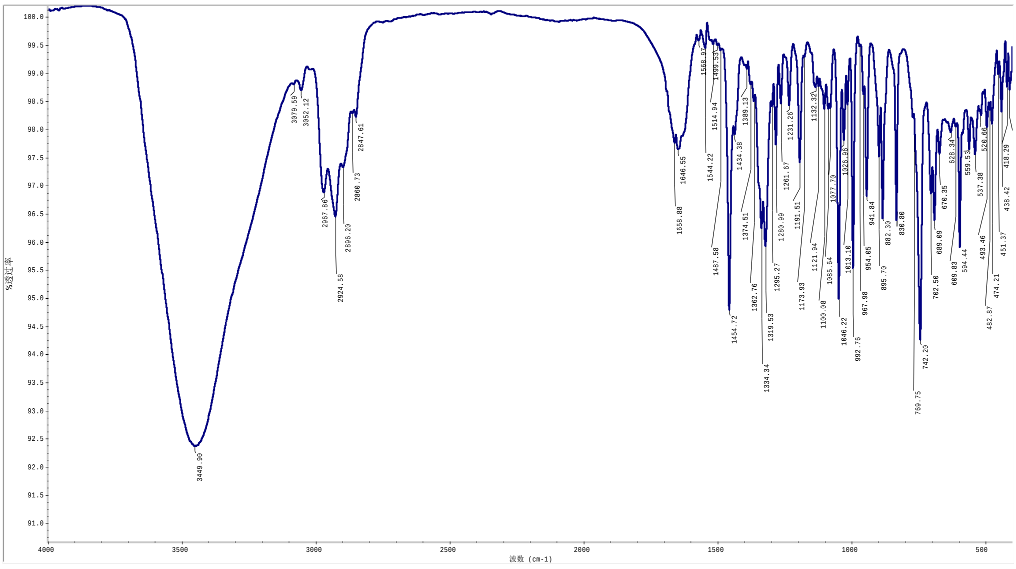

Sample Name: HBM-18a
KBr压片
采集时间: 星期五 7月 17 10:30:34 2020 (GMT+08:00)
仪器型号：NICOLET iS10
Software version: OMNIC 9.8.372

样品扫描次数: 16
背景扫描次数: 16
分辨率: 4.000
采样增益: 1.0
动镜速度: 0.4747
光阑: 80.00

Figure S18 ECD spectrum of bousmekine B (**2**)

HBM-18A

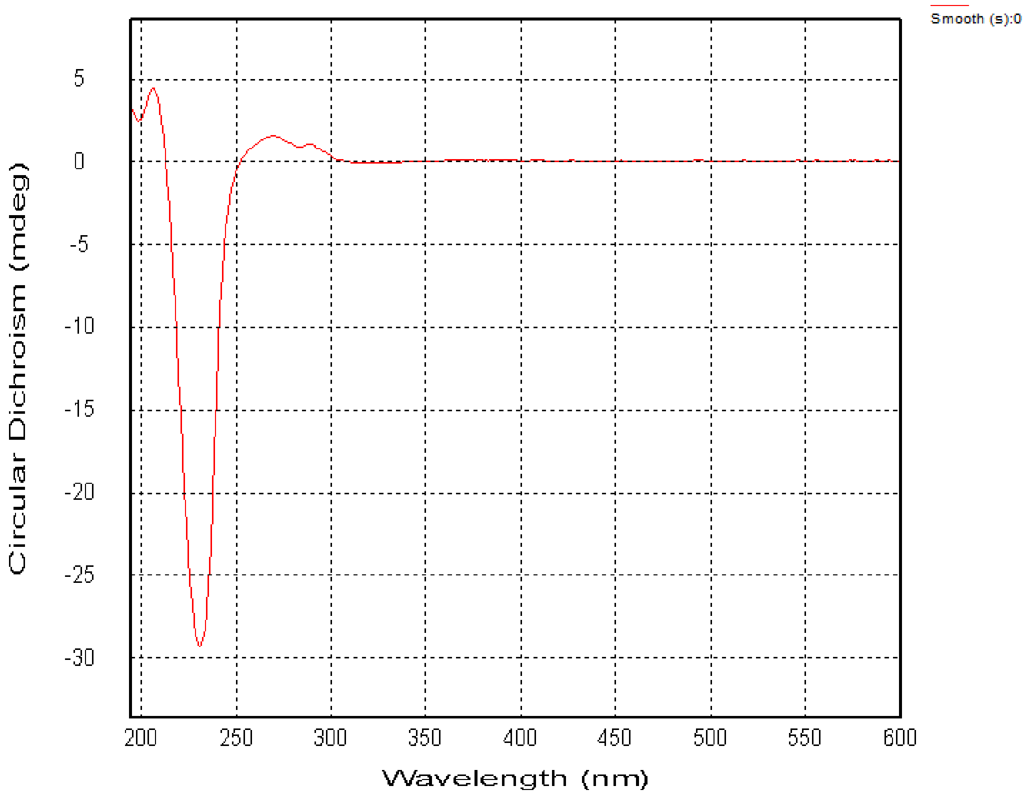

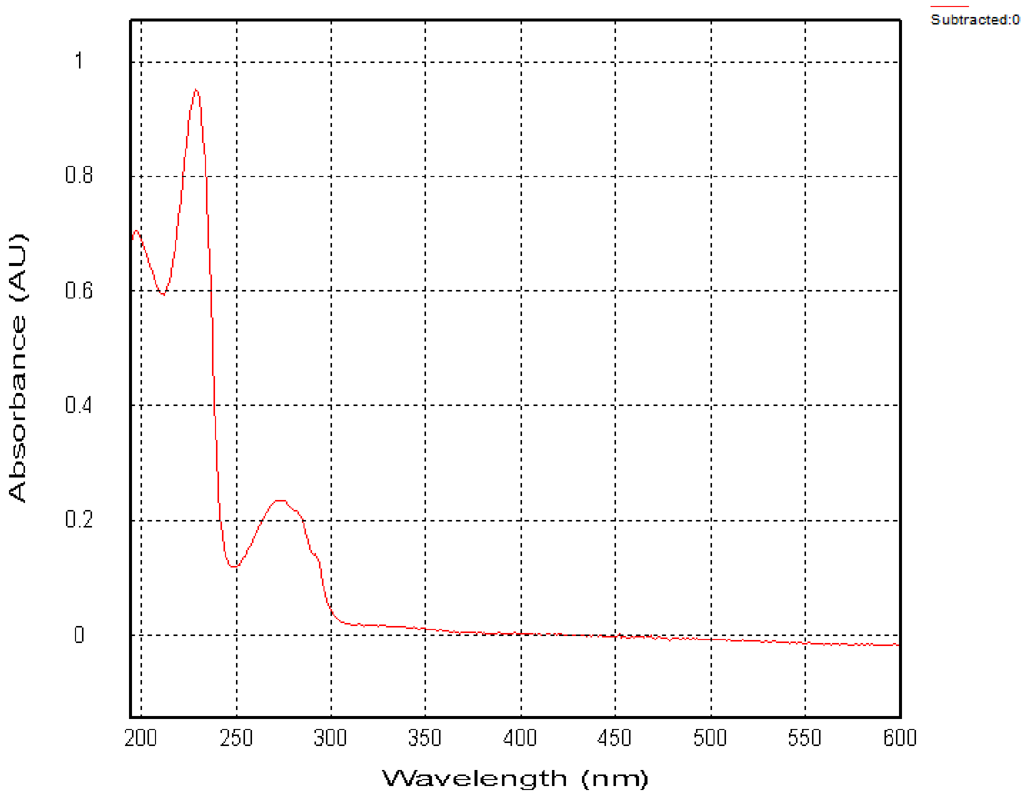

Figure S19 X-ray crystal structure of bousmekine (**2**)

Crystal data for bousmekine B (**2**): 4($C_{19}H_{21}ClN_2O$)•C_2H_6O, M = 1361.37, a = 11.9505(3) Å, b = 12.3727(3) Å, c = 22.7041(6) Å, α = 90°, β = 95.5750(10)°, γ = 90°, V = 3341.15(15) Å^3, T = 100.(2) K, space group P1211, Z = 2, μ(Cu Kα) = 2.091 mm^{-1}, 73809 reflections measured, 13008 independent reflections (R_{int} = 0.1435). The final R_1 values were 0.0675 ($I > 2\sigma(I)$). The final $wR(F^2)$ values were 0.1701 ($I > 2\sigma(I)$). The final R_1 values were 0.0798 (all data). The final $wR(F^2)$ values were 0.1860 (all data). The goodness of fit on F^2 was 1.030. Flack parameter = 0.104(12).

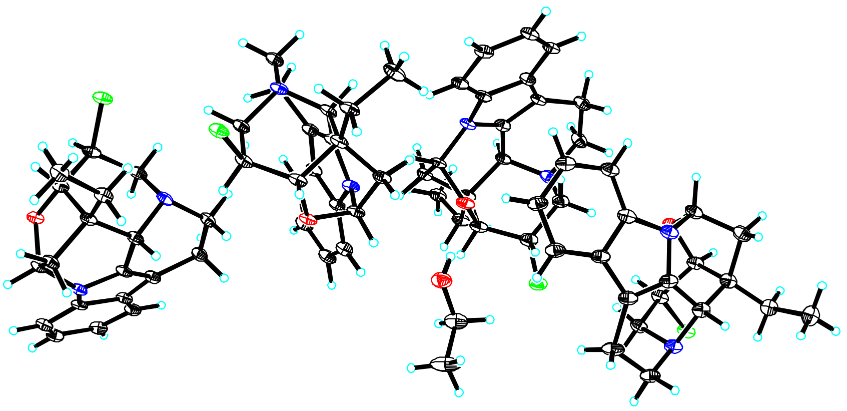

View of the molecules in an asymmetric unit.

Displacement ellipsoids are drawn at the 30% probability level.

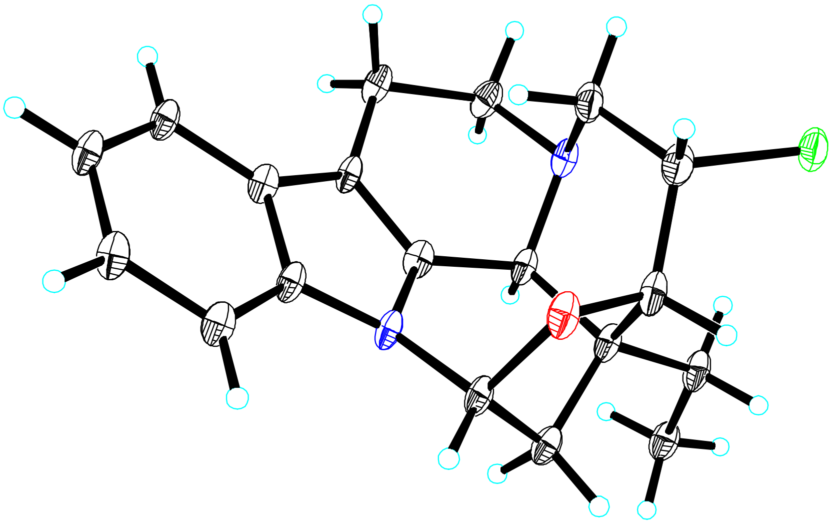

View of a molecule of fid25 with the atom-labelling scheme.

Displacement ellipsoids are drawn at the 30% probability level.

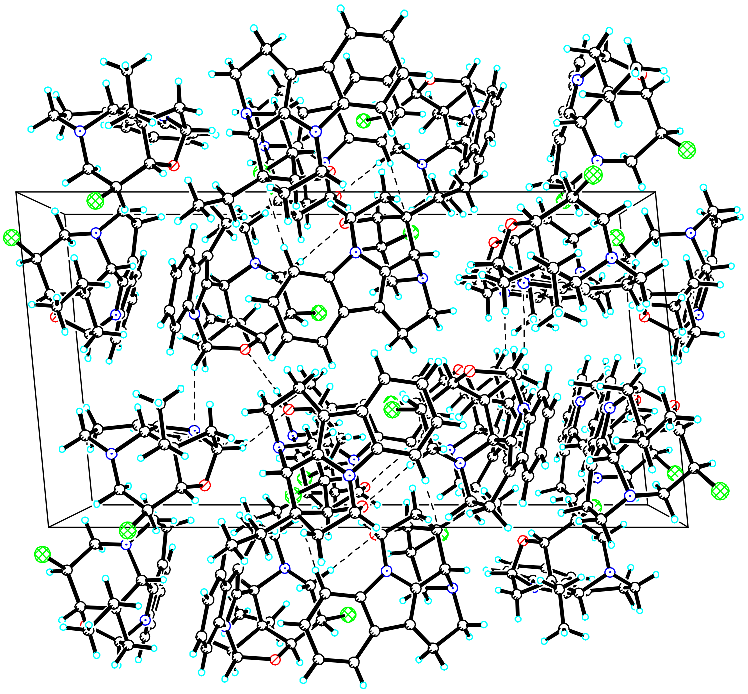

View of the pack drawing of fid25.
Hydrogen-bonds are shown as dashed lines.

Table 1. Crystal data and structure refinement for bousmekine B (**2**).

Identification code	global	
Empirical formula	C78 H90 Cl4 N8 O5	
Formula weight	1361.37	
Temperature	100(2) K	
Wavelength	1.54178 Å	
Crystal system	Monoclinic	
Space group	P 1 21 1	
Unit cell dimensions	a = 11.9505(3) Å	α= 90°.
	b = 12.3727(3) Å	β= 95.5750(10)°.
	c = 22.7041(6) Å	γ = 90°.
Volume	3341.15(15) Å^3	
Z	2	

Density (calculated)	1.353 Mg/m^3
Absorption coefficient	2.091 mm^{-1}
F(000)	1444
Crystal size	0.400 x 0.330 x 0.010 mm^3
Theta range for data collection	3.72 to 72.33°.
Index ranges	-14<=h<=14, -15<=k<=14, -27<=l<=28
Reflections collected	73809
Independent reflections	13008 [R(int) = 0.1435]
Completeness to theta = 72.33°	99.9 %
Absorption correction	Semi-empirical from equivalents
Max. and min. transmission	0.98 and 0.45
Refinement method	Full-matrix least-squares on F^2
Data / restraints / parameters	13008 / 1 / 863
Goodness-of-fit on F^2	1.030
Final R indices [I>2sigma(I)]	R1 = 0.0675, wR2 = 0.1701
R indices (all data)	R1 = 0.0798, wR2 = 0.1860
Absolute structure parameter	0.104(12)
Extinction coefficient	0.0015(3)
Largest diff. peak and hole	0.578 and -1.134 e.Å^{-3}

Figure S20 ^{1}H NMR spectrum of bousmekine C (**3**) in CD_3OD

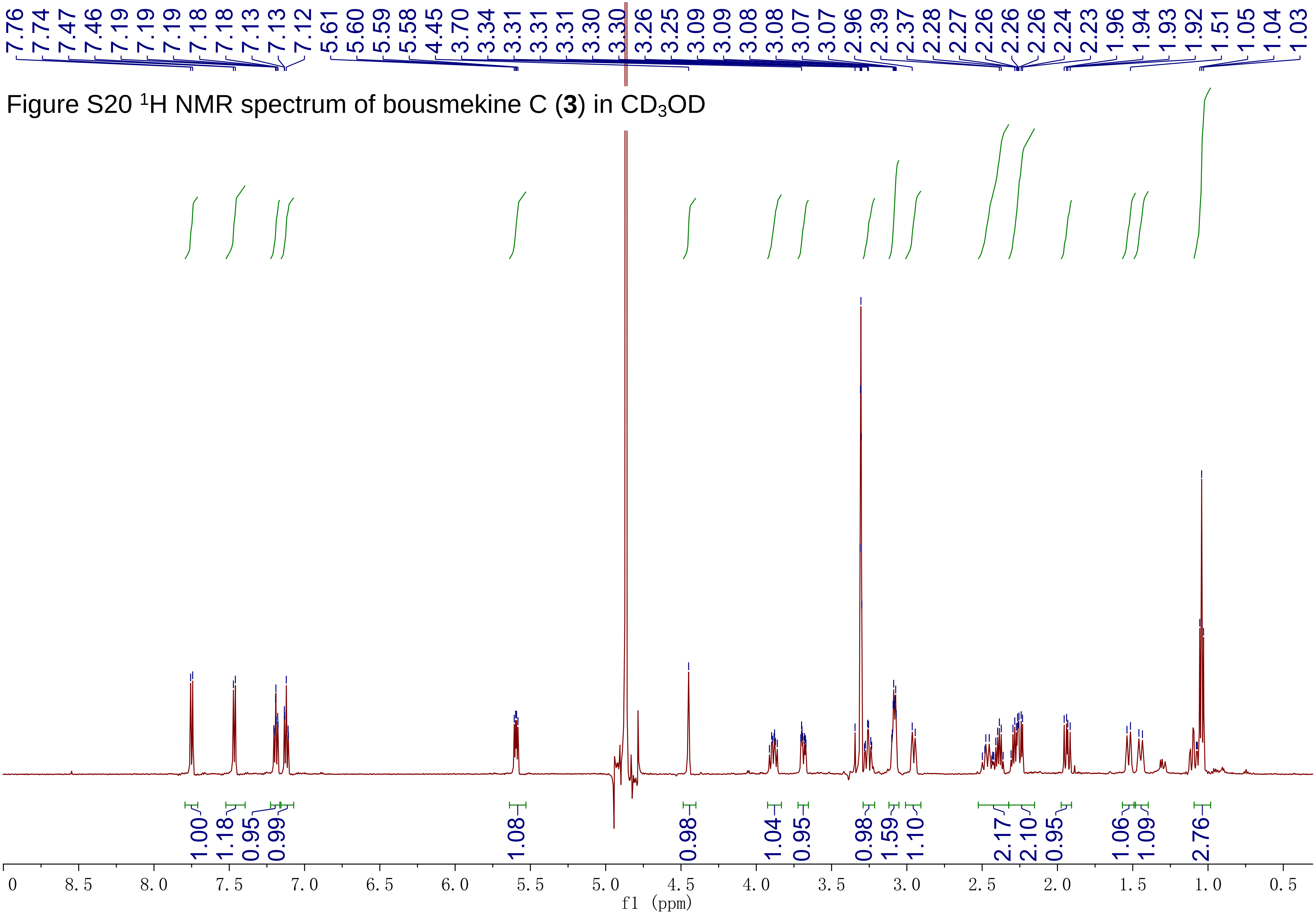

Figure S21 ^{13}C NMR spectrum of bousmekine C (**3**) in CD_3OD

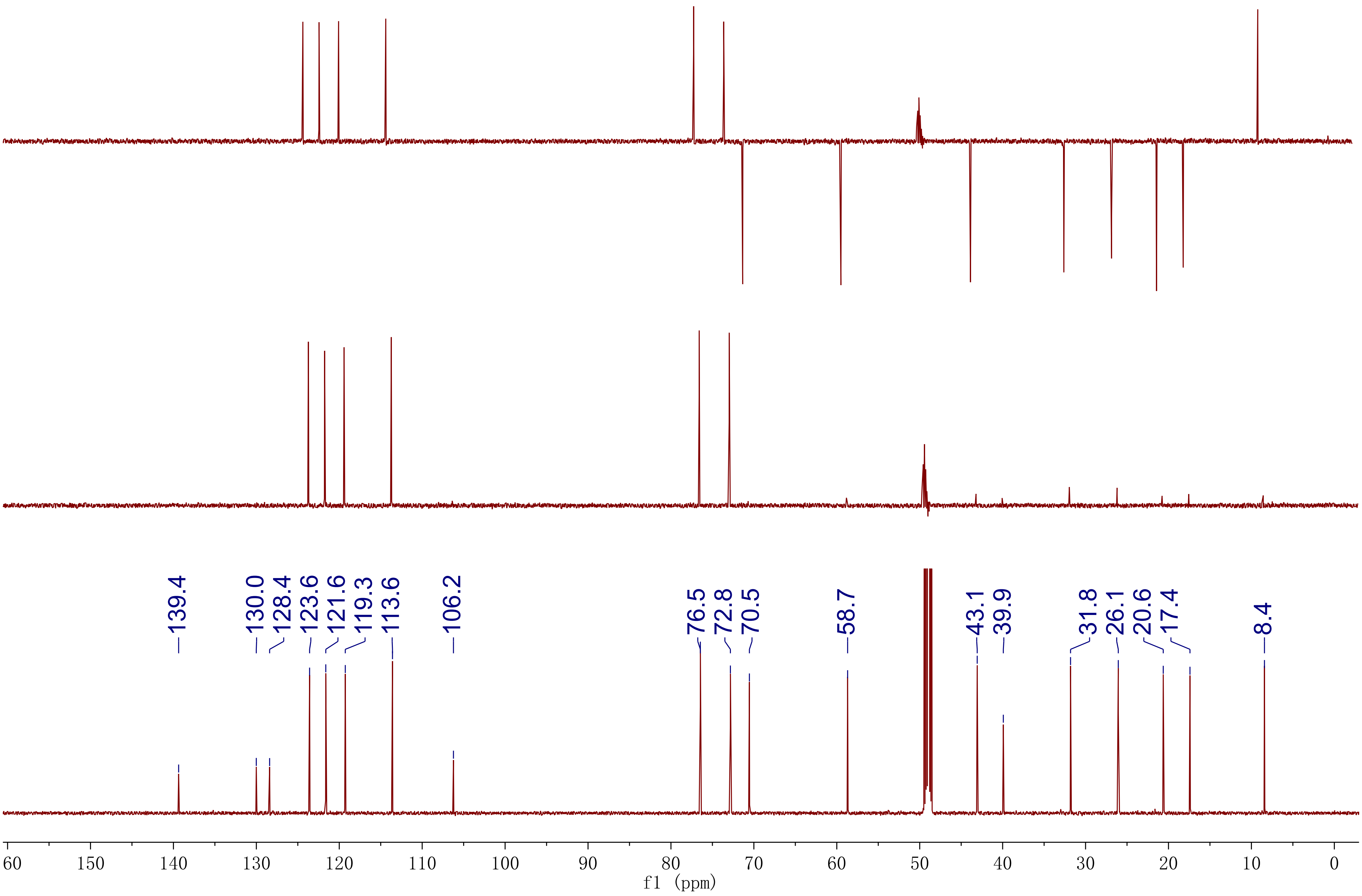

Figure S22 HSQC spectrum of bousmekine C (**3**) in CD_3OD

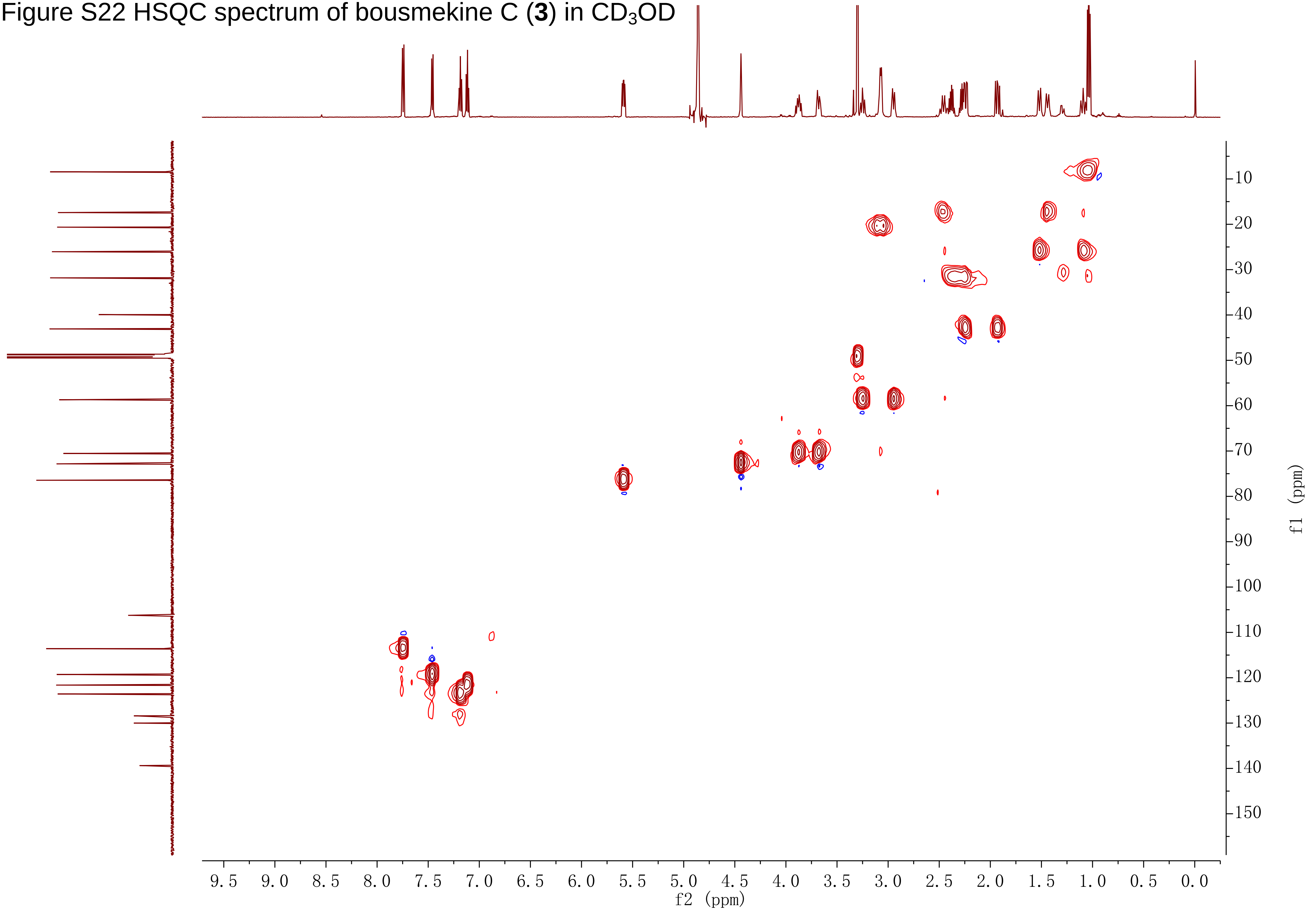

Figure S23 ^1H-^1H COSY spectrum of bousmekine C (**3**) in CD_3OD

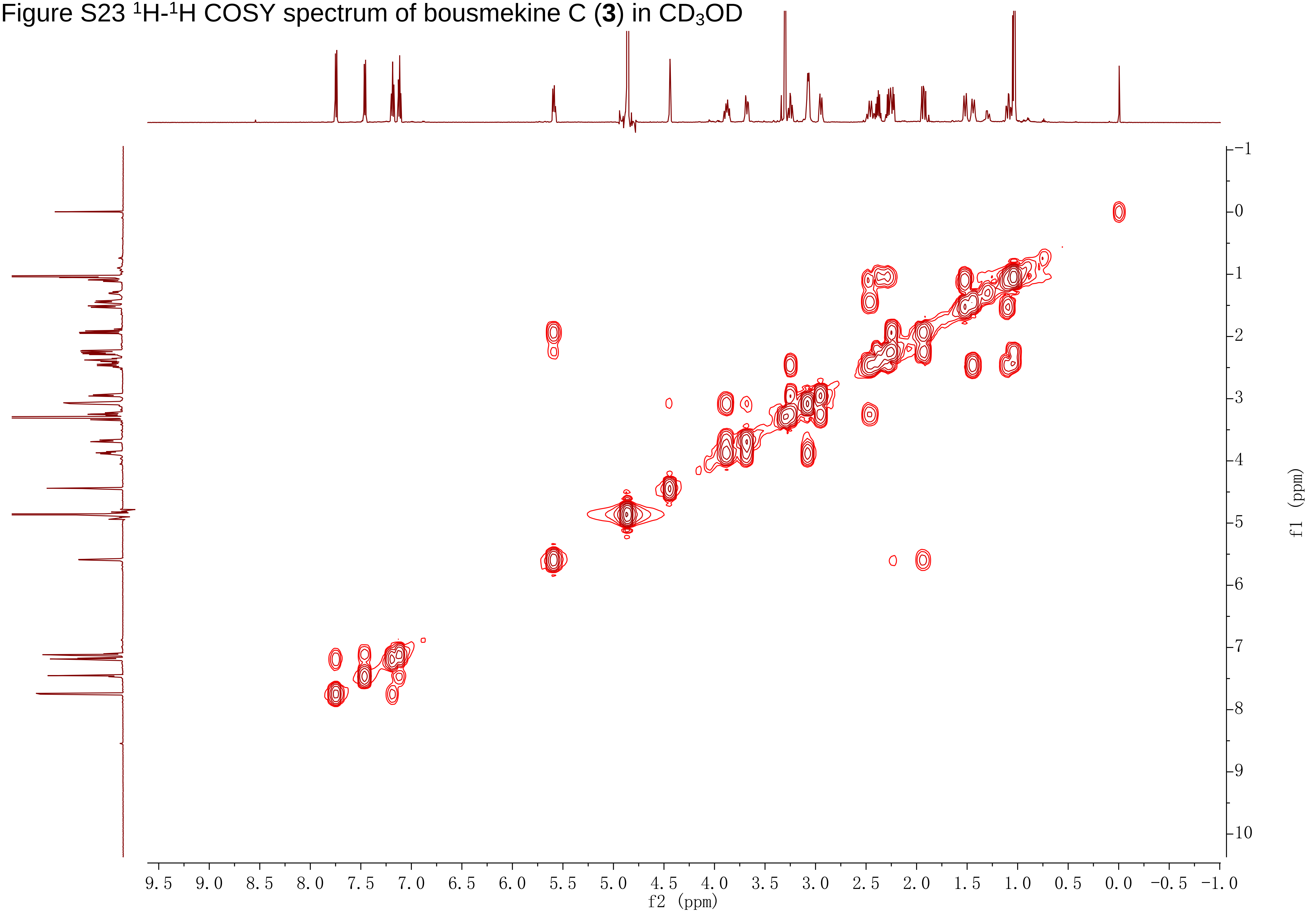

Figure S24 HMBC spectrum of bousmekine C (**3**) in CD_3OD

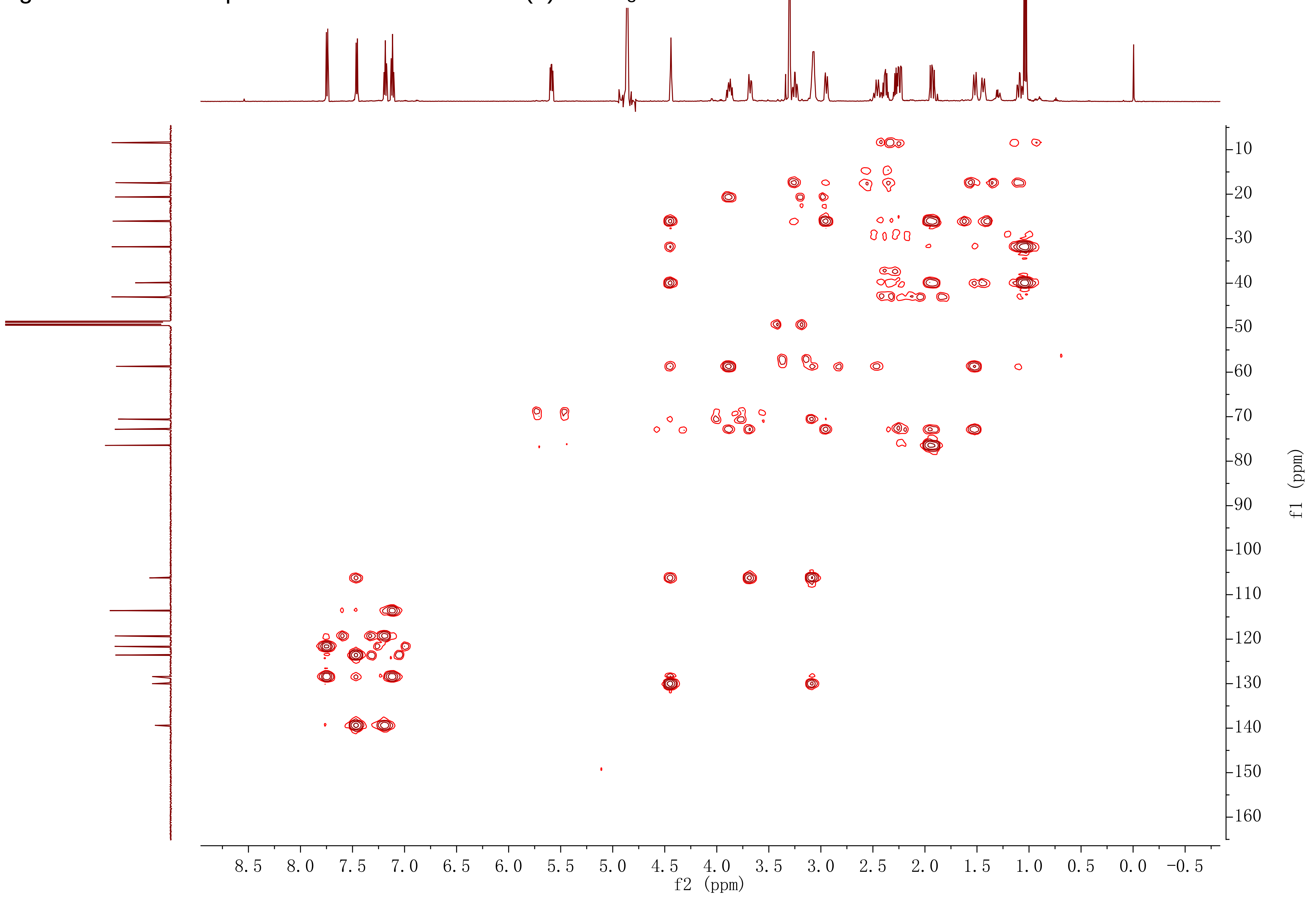

Figure S25 ROESY spectrum of bousmekine C (**3**) in CD_3OD

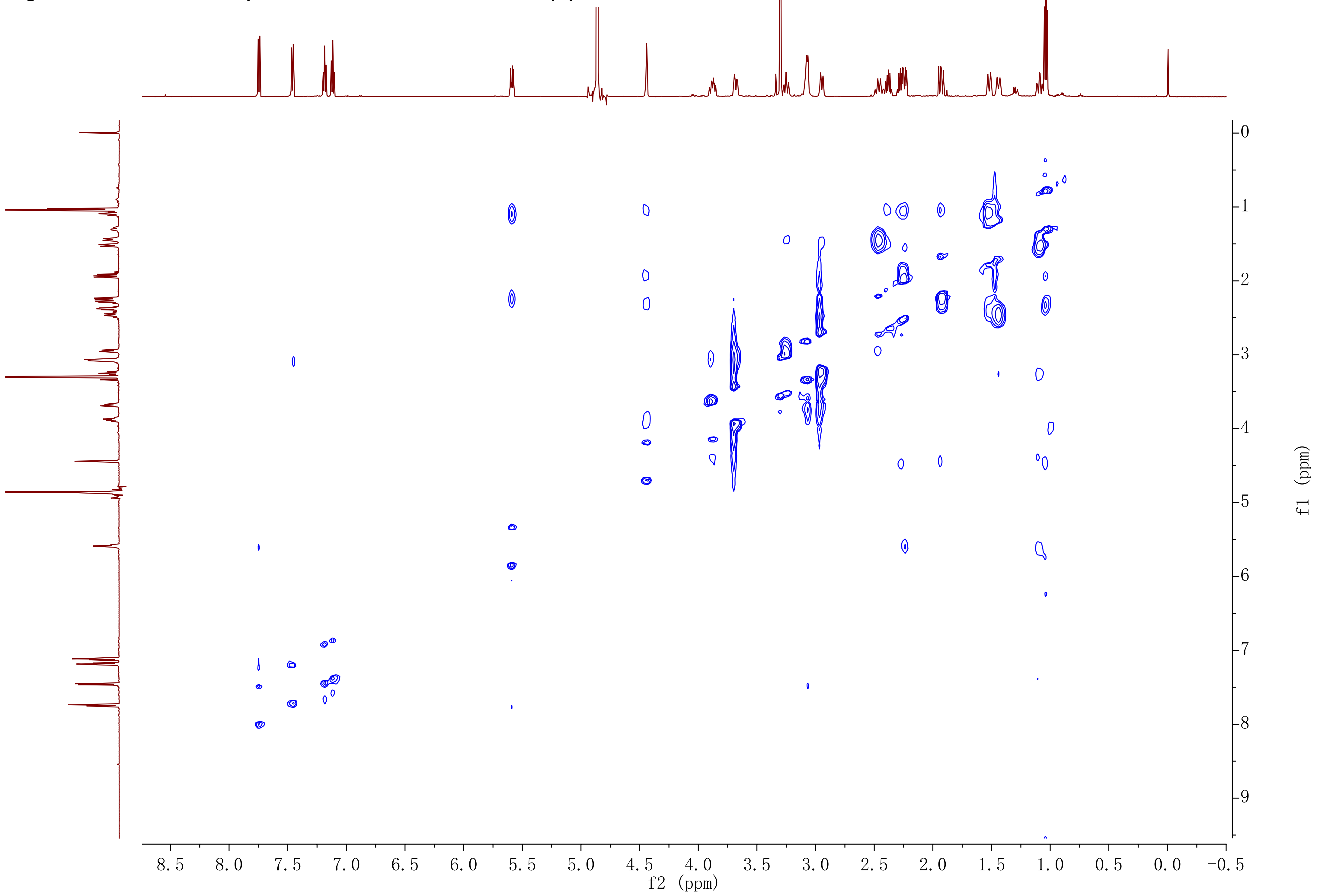

Figure S28 ECD spectrum of bousmekine C (**3**)

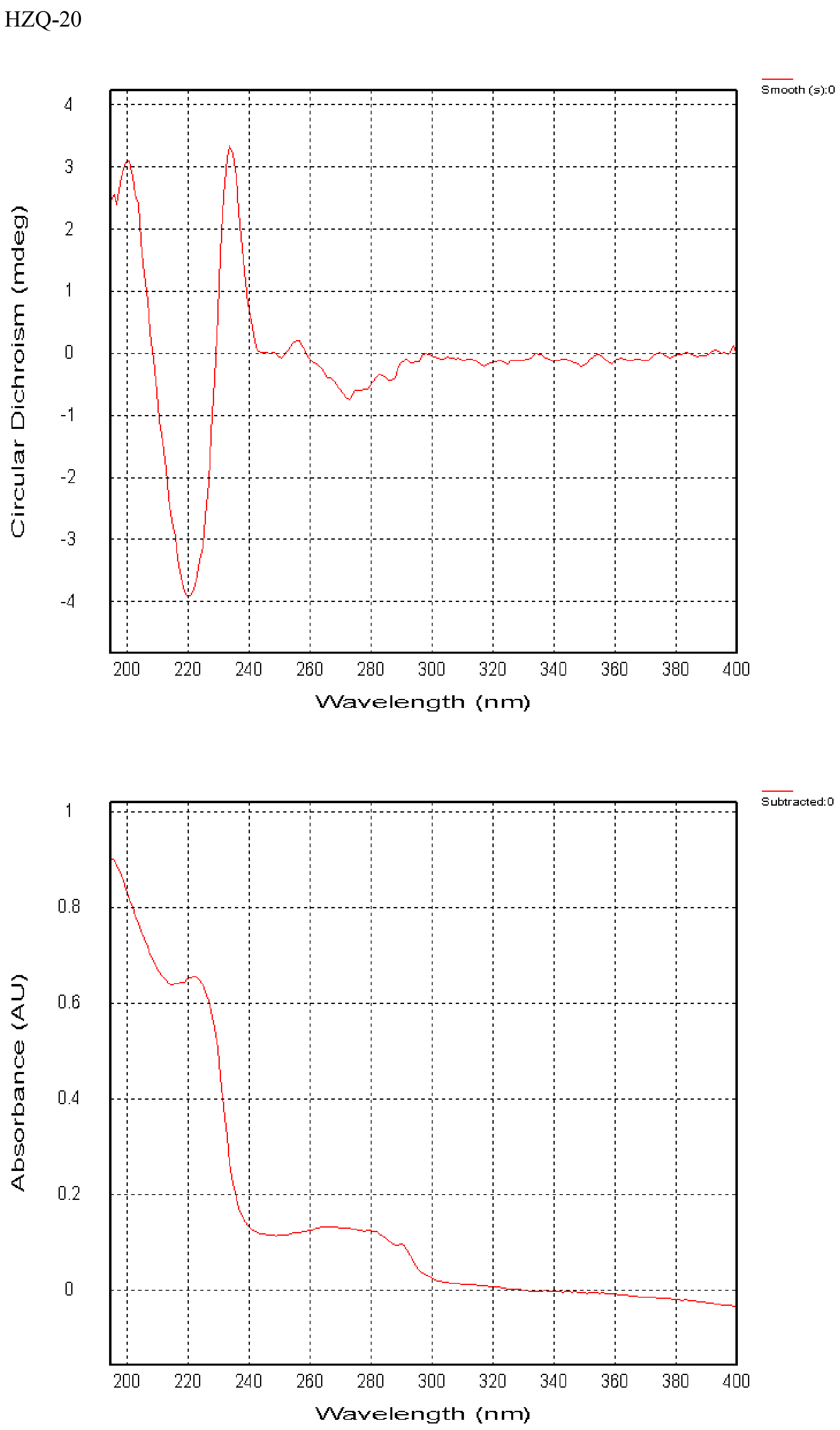

Figure S26 HRESIMS spectrums of bousmekine C (**3**)

Qualitative Analysis Report

**Data Filename**	HZQ-20.d	**Sample Name**	HZQ-20
**Sample Type**	Sample	**Position**	P1-A3
**Instrument Name**	Instrument 1	**User Name**	
**Acq Method**	s.m	**Acquired Time**	7/8/2020 2:44:57 PM
**IRM Calibration Status**	Success	**DA Method**	Default.m
**Comment**			

**Sample Group**		**Info.**
**Acquisition SW Version**	6200 series TOF/6500 series Q-TOF B.05.01 (B5125.2)	

User Spectra

Fragmentor Voltage	Collision Energy	Ionization Mode
135	0	ESI

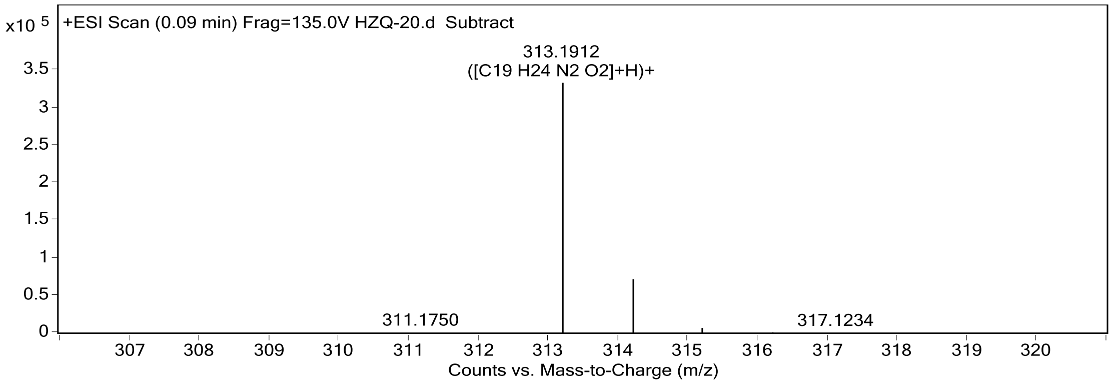

Peak List

m/z	z	Abund	Formula	Ion
296.187		2367.64		
313.1912	1	333377.84	C19 H24 N2 O2	(M+H)+
314.1941	1	71725.34	C19 H24 N2 O2	(M+H)+
315.1963	1	8302.45	C19 H24 N2 O2	(M+H)+
316.2004	1	1121.25	C19 H24 N2 O2	(M+H)+
335.1725	1	5471.92		
336.176	1	1140.25		
625.3756	1	57262.24		
626.3786	1	25075.63		
627.381	1	5175.1		

Formula Calculator Element Limits

Element	Min	Max
C	3	60
H	0	120
O	0	30
N	0	10

Formula Calculator Results

Formula	CalculatedMass	CalculatedMz	Mz	Diff. (mDa)	Diff. (ppm)	DBE
C19 H24 N2 O2	312.1838	313.1911	313.1912	-0.10	-0.32	9.0000

--- End Of Report ---

Figure S27 IR spectrum of bousmekine C (**3**)

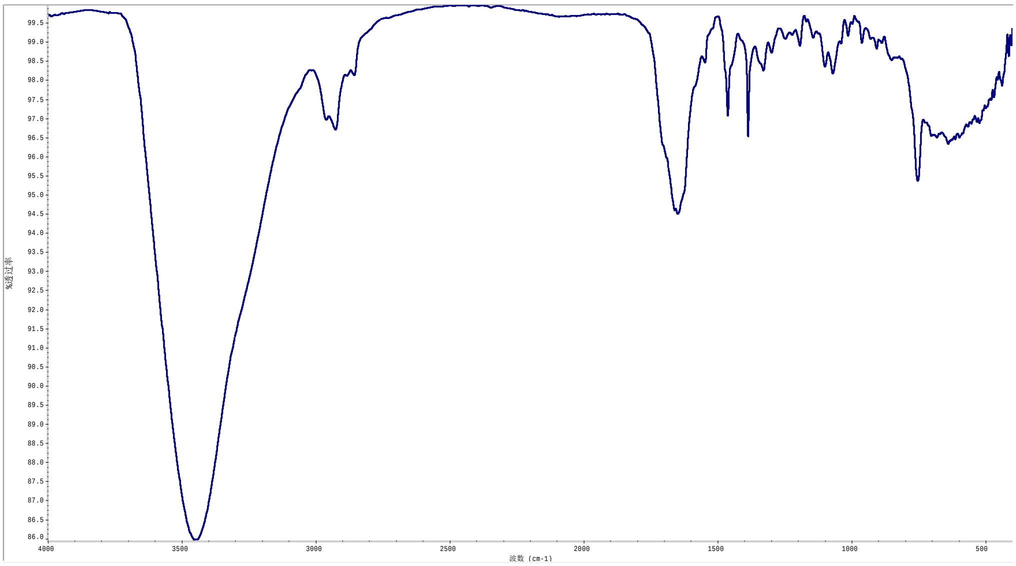

Sample Name: HZQ-20
KBr压片
采集时间: 星期五 7月 17 09:40:57 2020 (GMT+08:00)
仪器型号：NICOLET iS10
Software version: OMNIC 9.8.372

样品扫描次数: 16
背景扫描次数: 16
分辨率: 4.000
采样增益: 1.0
动镜速度: 0.4747
光阑: 80.00

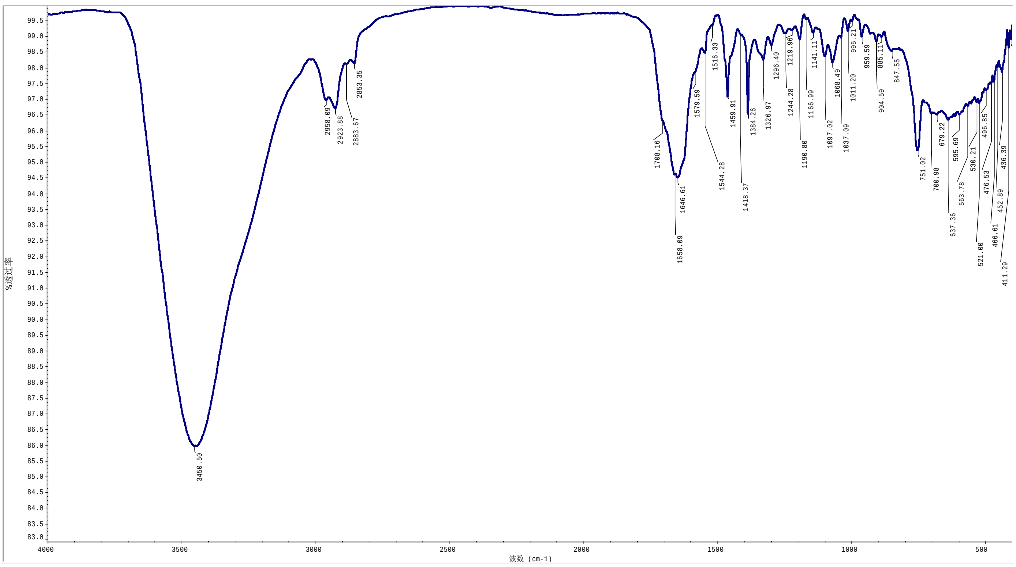

Sample Name: HZQ-20
KBr压片
采集时间: 星期五 7月 17 09:40:57 2020 (GMT+08:00)
仪器型号：NICOLET iS10
Software version: OMNIC 9.8.372

样品扫描次数: 16
背景扫描次数: 16
分辨率: 4.000
采样增益: 1.0
动镜速度: 0.4747
光阑: 80.00

Figure S29 ^{1}H NMR spectrum of bousmekine D (**4**) in CD_3OD

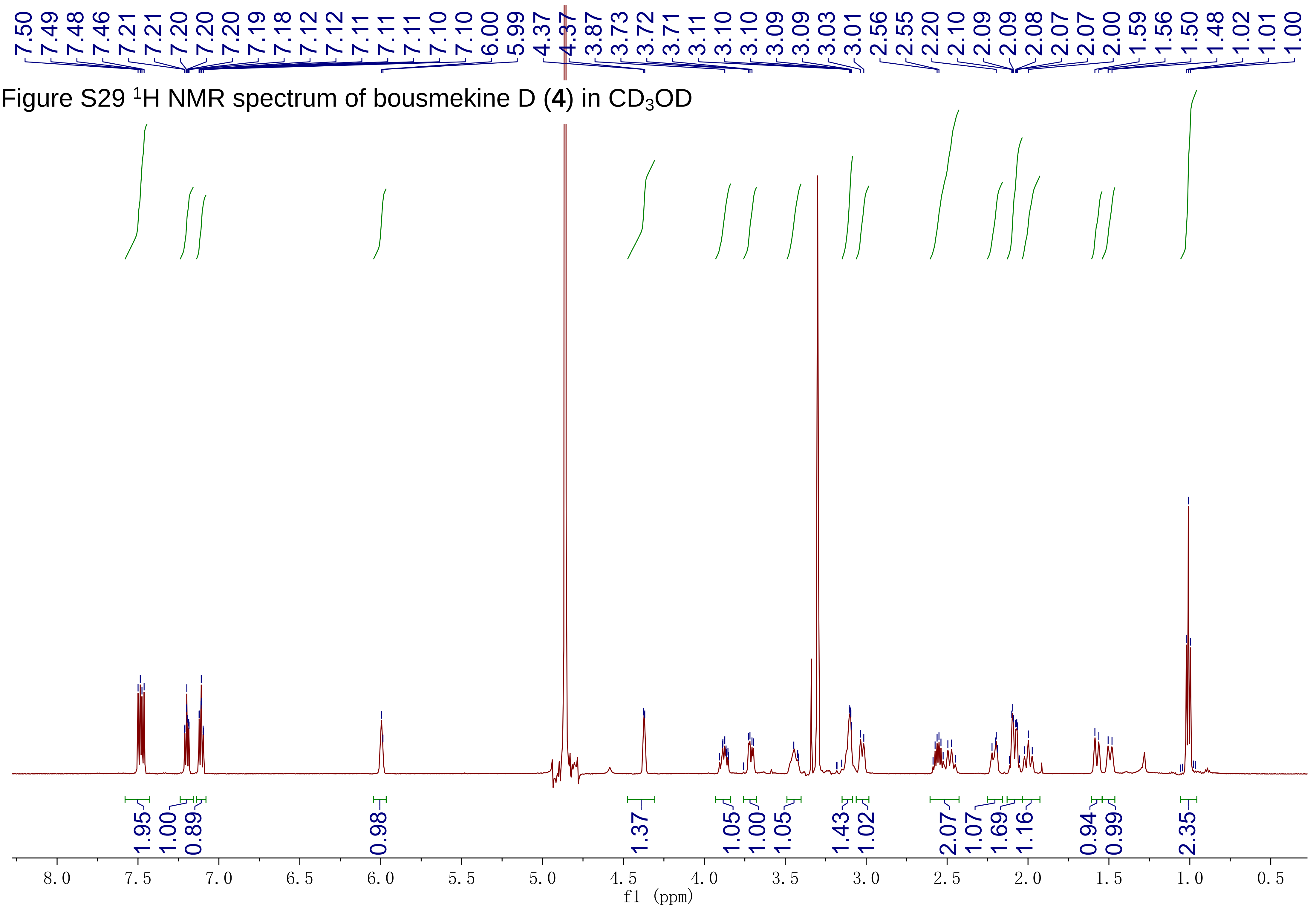

Figure S30 ^{13}C NMR spectrum of bousmekine D (**4**) in CD_3OD

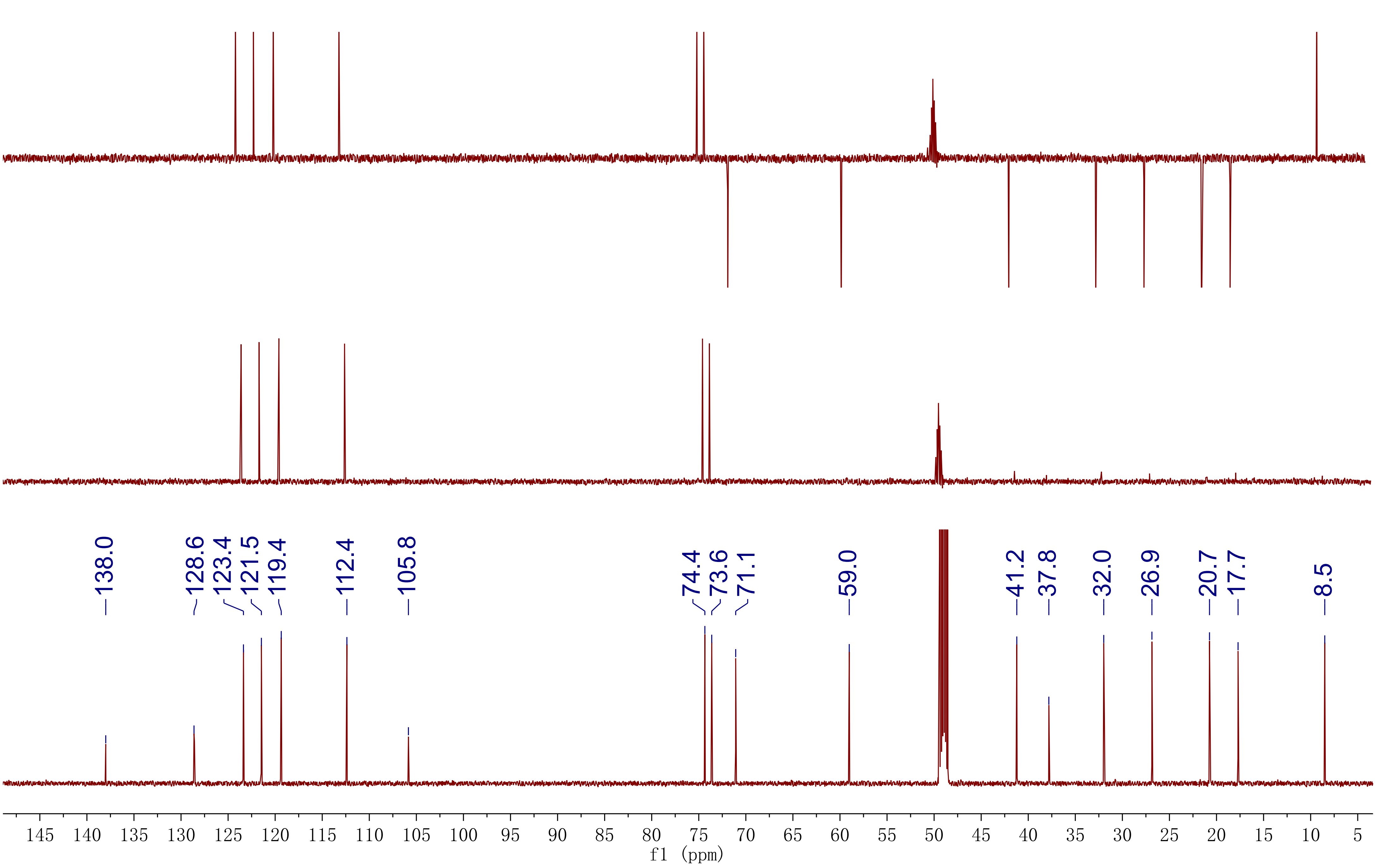

Figure S31 HSQC spectrum of bousmekine D (**4**) in CD_3OD

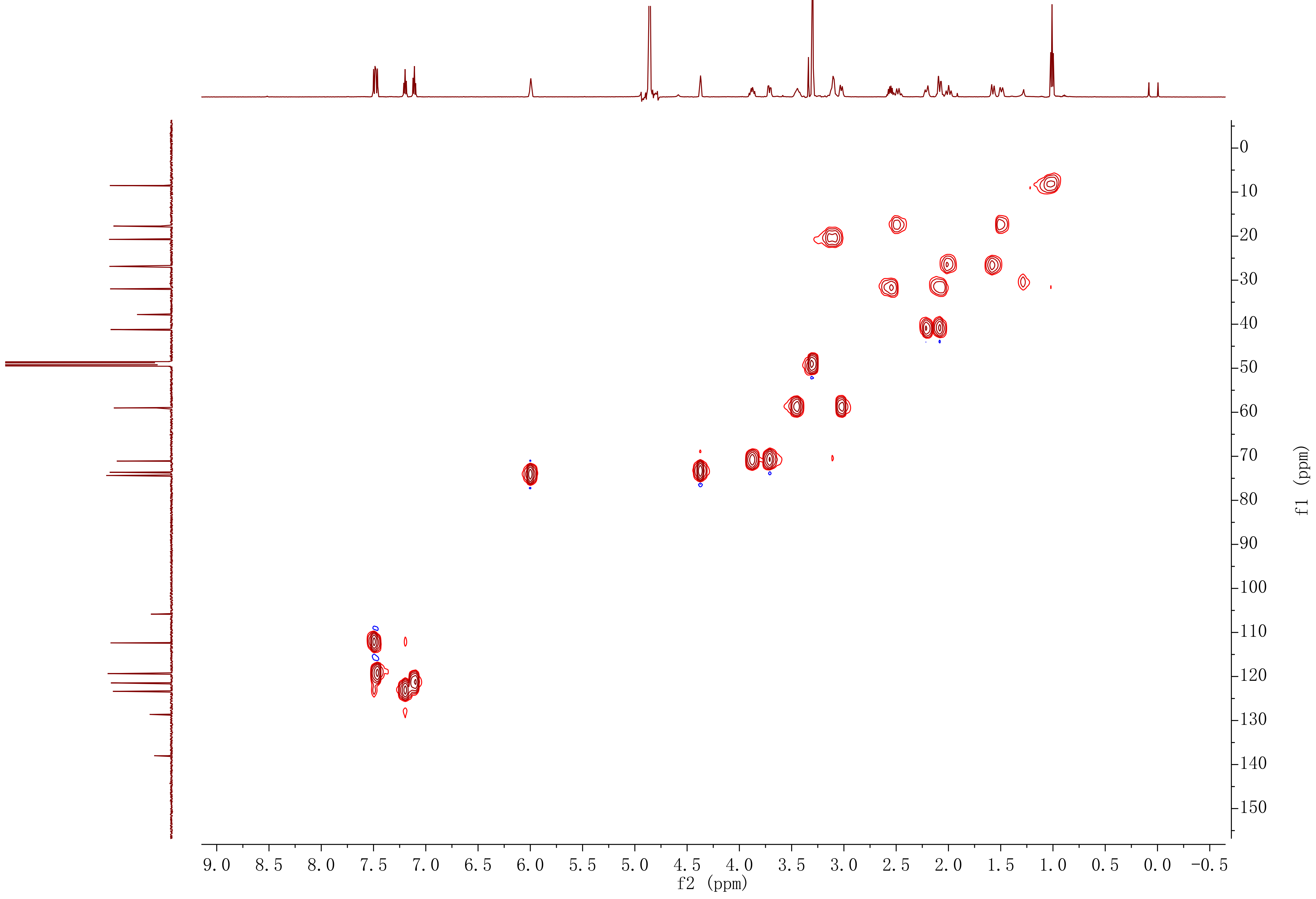

Figure S32 ^{1}H-^{1}H COSY spectrum of bousmekine D (4) in CD_3OD

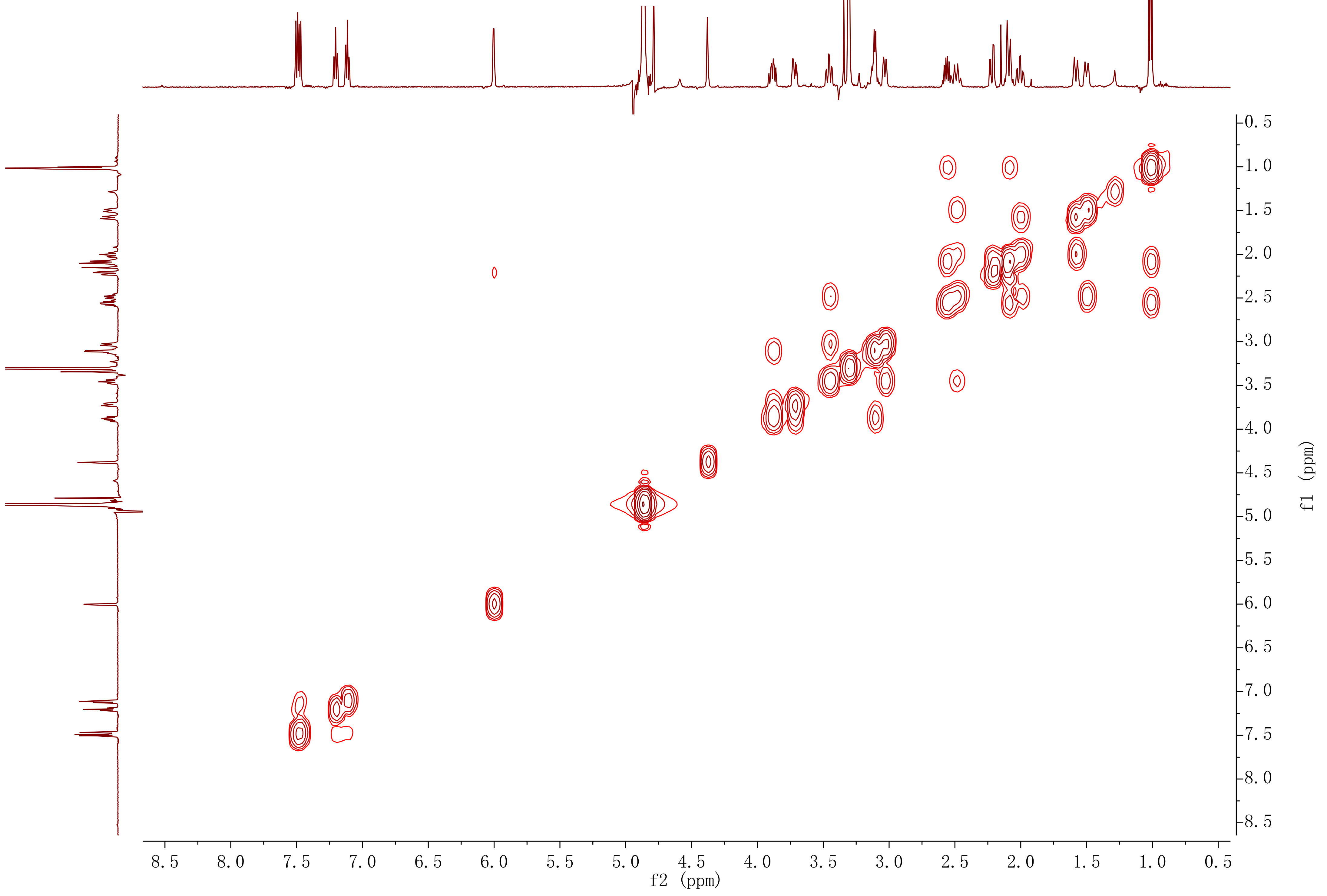

Figure S33 HMBC spectrum of bousmekine D (**4**) in CD_3OD

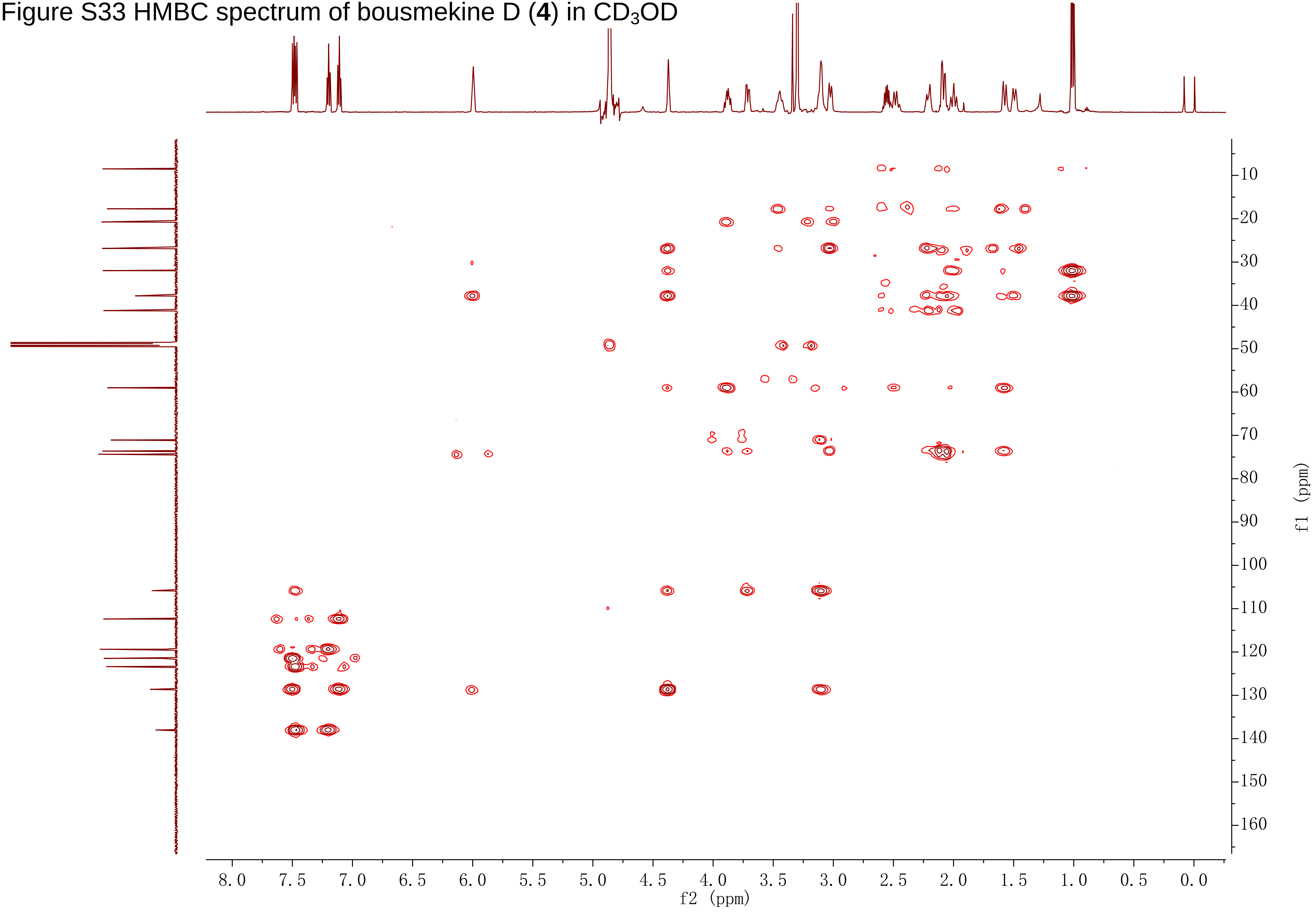

Figure S34 ROESY spectrum of bousmekine D (**4**) in CD_3OD

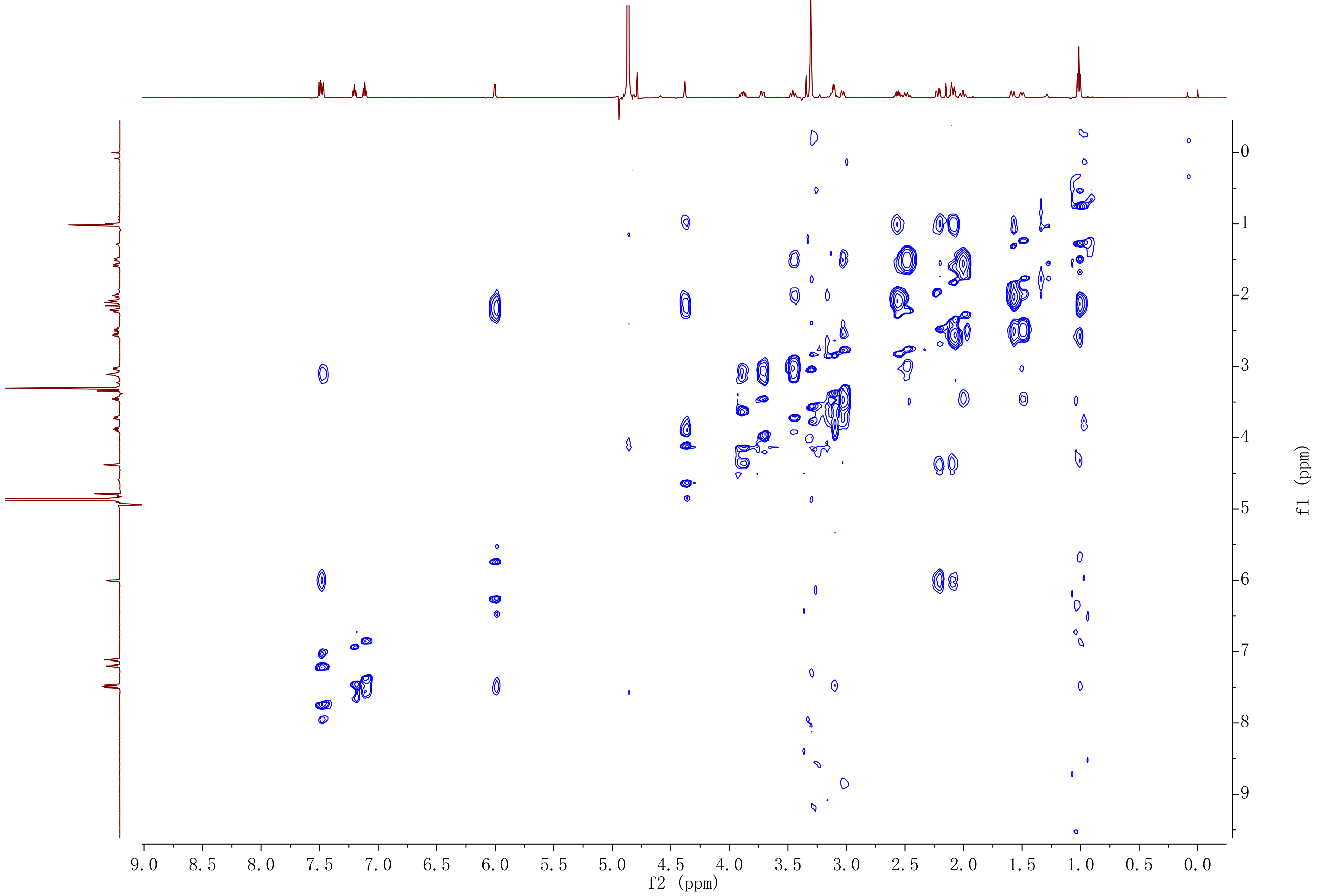

Figure S35 HRESIMS spectrums of bousmekine D (**4**)

Qualitative Analysis Report

**Data Filename**	HZQ-20-2.d	**Sample Name**	HZQ-20-2
**Sample Type**	Sample	**Position**	P1-A4
**Instrument Name**	Instrument 1	**User Name**	
**Acq Method**	s.m	**Acquired Time**	7/8/2020 2:46:08 PM
**IRM Calibration Status**	Success	**DA Method**	Default.m
**Comment**			

Sample Group **Info.**

Acquisition SW Version 6200 series TOF/6500 series Q-TOF B.05.01 (B5125.2)

User Spectra

Fragmentor Voltage	Collision Energy	Ionization Mode
135	0	ESI

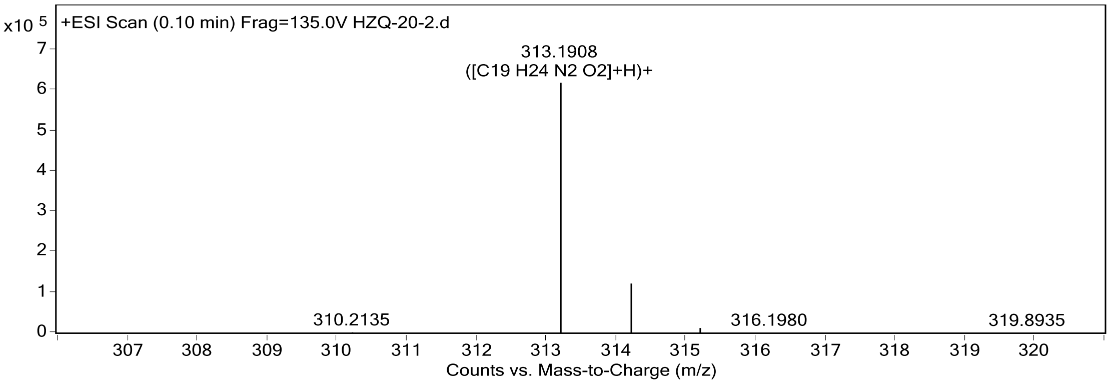

Peak List

m/z	z	Abund	Formula	Ion
128.9528		18887.91		
150.1115	1	14905.11		
172.0936	1	16877.32		
313.1908	1	619139.44	C19 H24 N2 O2	(M+H)+
314.1937	1	122459.3	C19 H24 N2 O2	(M+H)+
327.206	1	107916.5		
328.2088	1	25436.25		
625.3739	1	68373.75		
626.3768	1	28489.15		
639.3893	1	35473.44		

Formula Calculator Element Limits

Element	Min	Max
C	3	60
H	0	120
O	0	30
N	0	10

Formula Calculator Results

Formula	CalculatedMass	CalculatedMz	Mz	Diff. (mDa)	Diff. (ppm)	DBE
C19 H24 N2 O2	312.1838	313.1911	313.1908	0.30	0.96	9.0000

--- End Of Report ---

Figure S36 IR spectrum of bousmekine D (**4**)

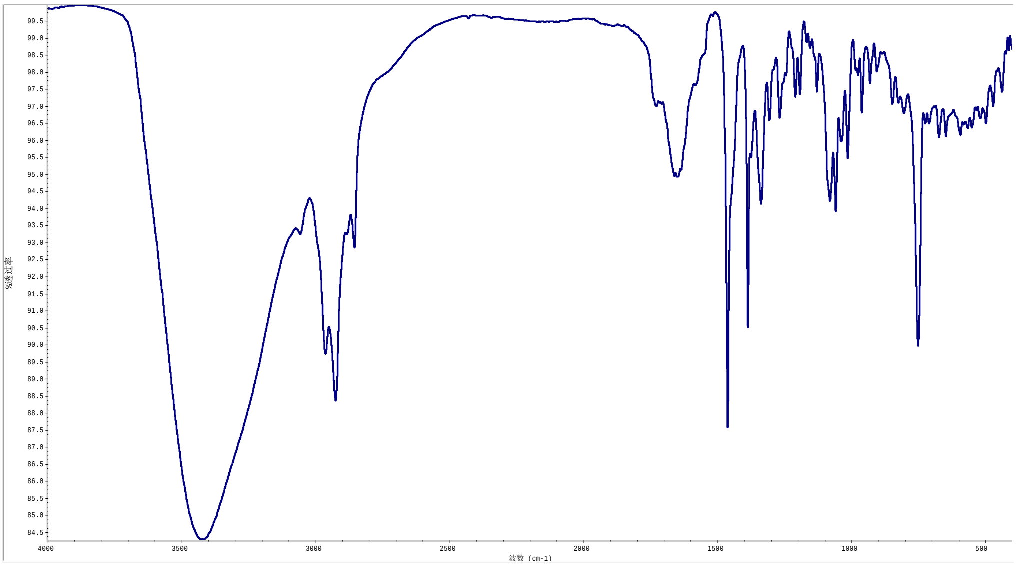

Sample Name: HZQ-202
KBr压片
采集时间: 星期五 7月 17 10:08:45 2020 (GMT+08:00)
仪器型号：NICOLET iS10
Software version: OMNIC 9.8.372

样品扫描次数: 16
背景扫描次数: 16
分辨率: 4.000
采样增益: 1.0
动镜速度: 0.4747
光阑: 80.00

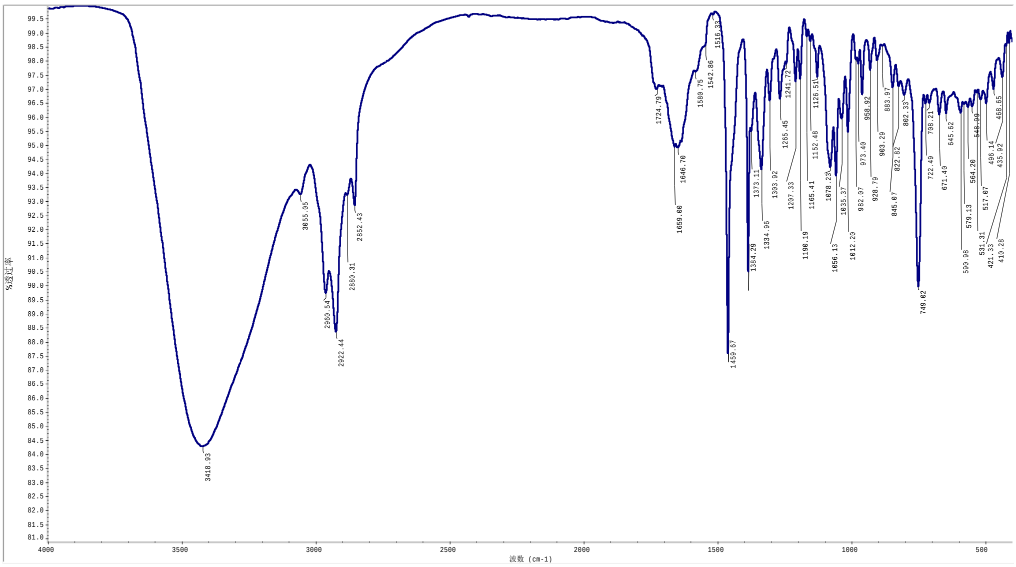

Sample Name: HZQ-202
KBr压片
采集时间: 星期五 7月 17 10:08:45 2020 (GMT+08:00)
仪器型号：NICOLET iS10
Software version: OMNIC 9.8.372

样品扫描次数: 16
背景扫描次数: 16
分辨率: 4.000
采样增益: 1.0
动镜速度: 0.4747
光阑: 80.00

Figure S37 ECD spectrum of bousmekine D (**4**)

HZQ-20-2

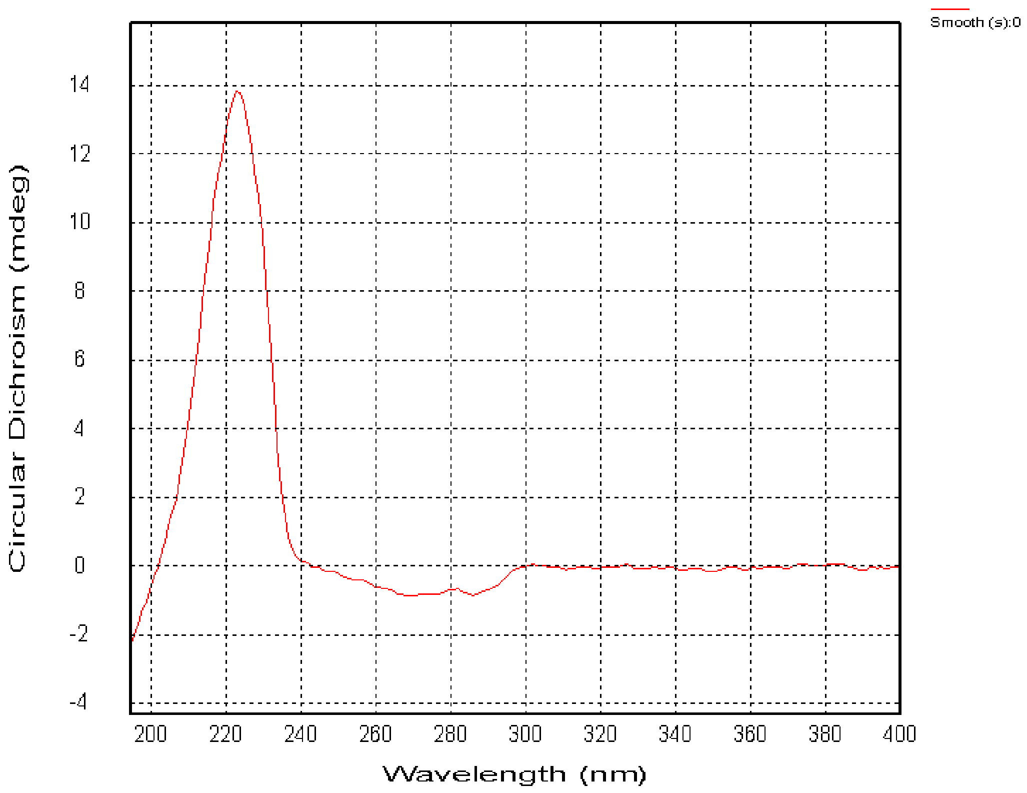

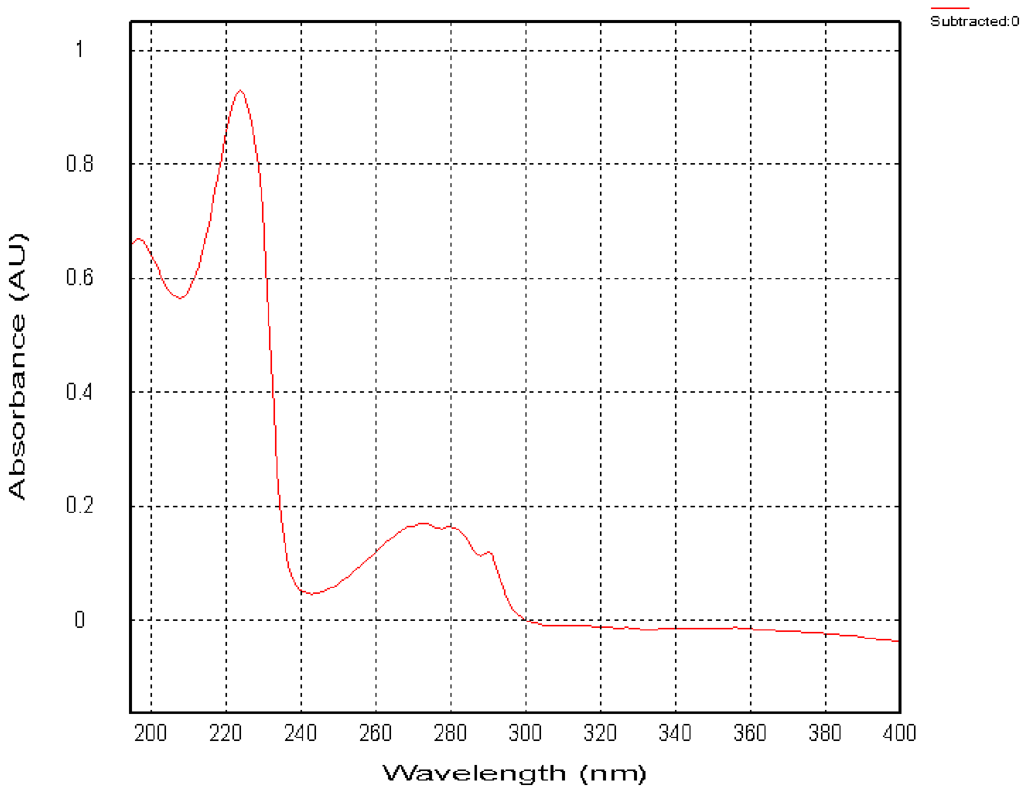

Figure S38 ^{1}H NMR spectrum of bousmekine E (**5**) in CD_3COCD_3

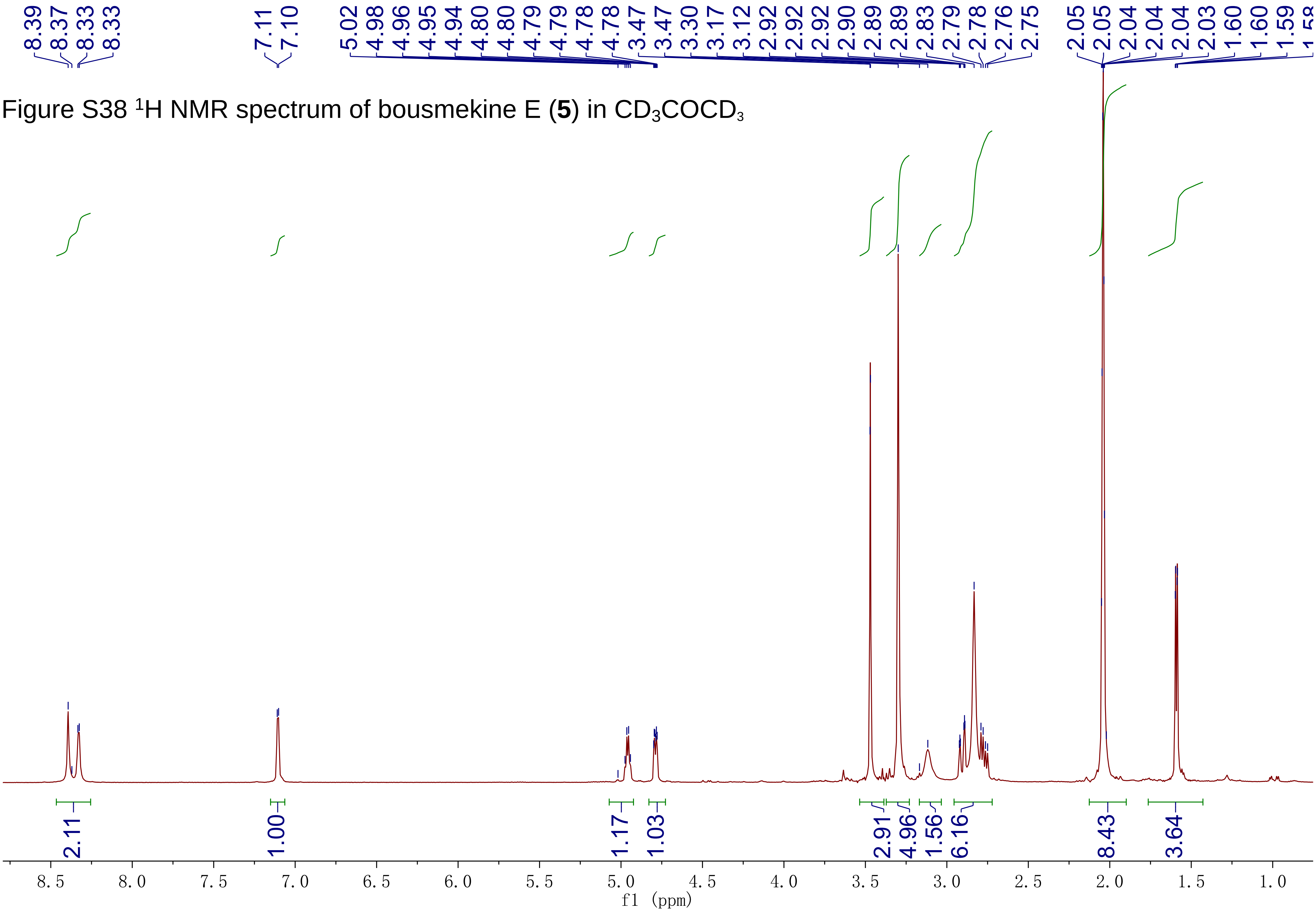

Figure S39 ^{13}C NMR spectrum of bousmekine E (**5**) in CD_3COCD_3

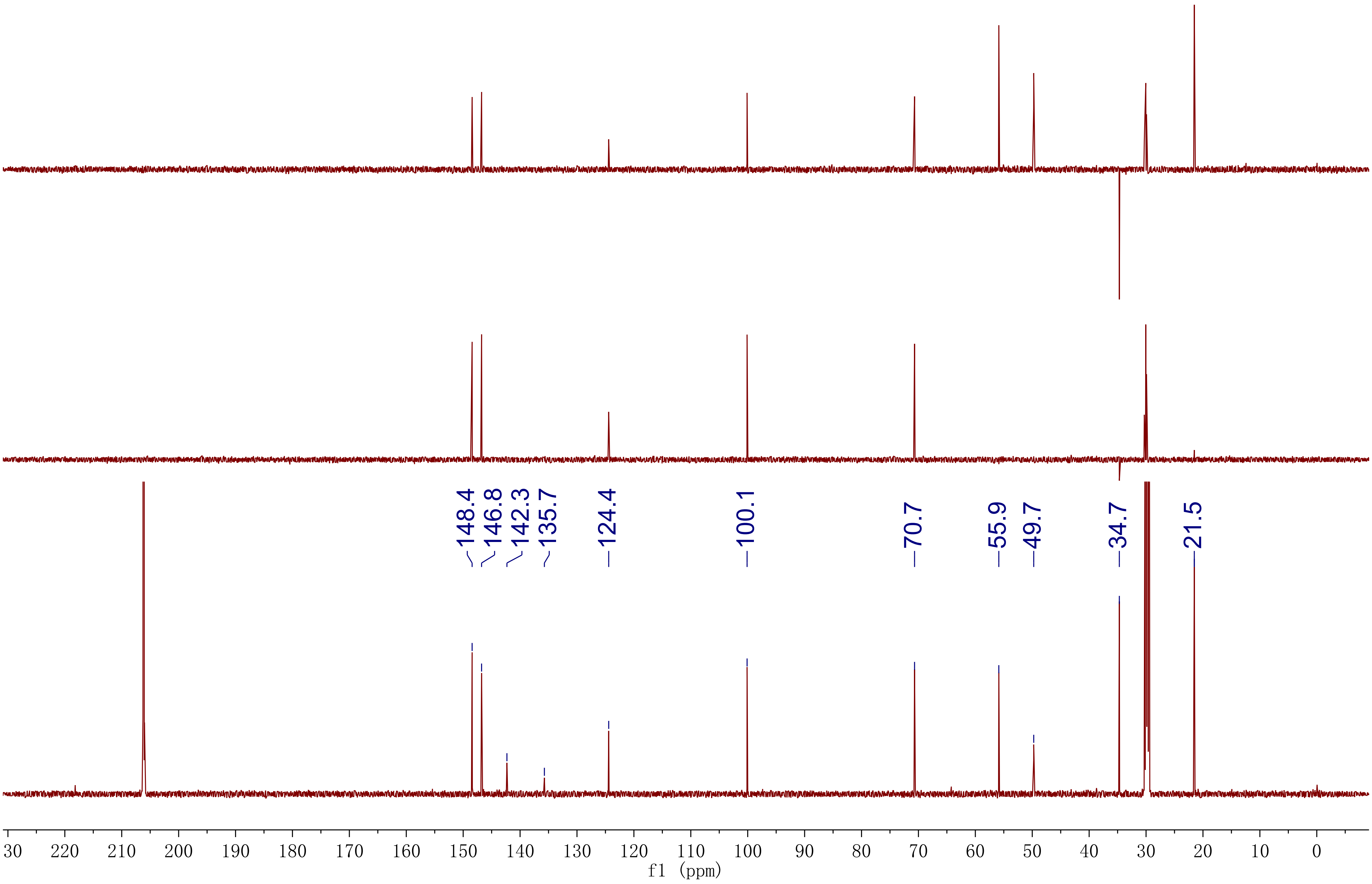

Figure S40 HSQC spectrum of bousmekine E (**5**) in CD_3COCD_3

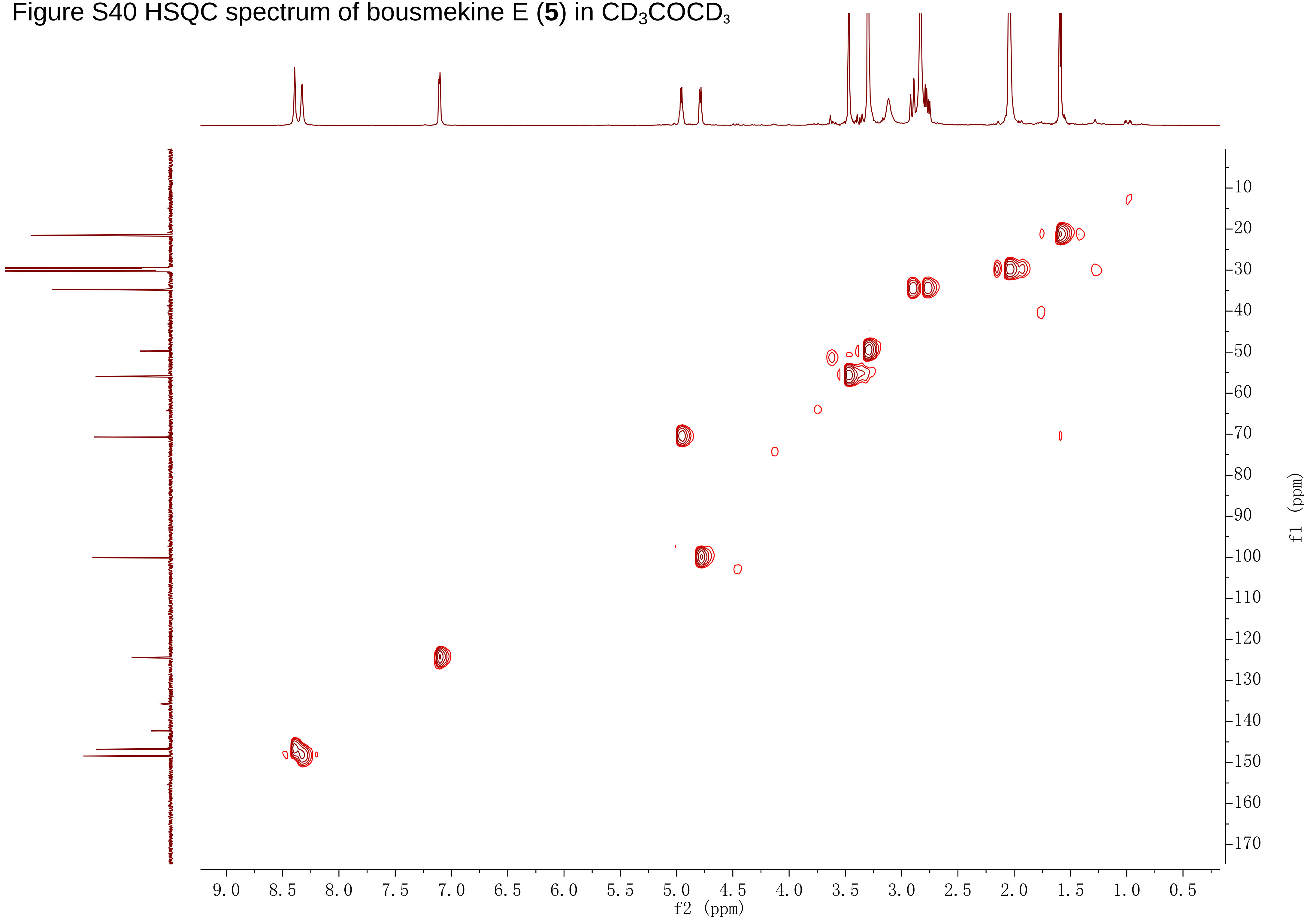

Figure S41 ^{1}H-^{1}H COSY spectrum of bousmekine E (**5**) in CD_3COCD_3

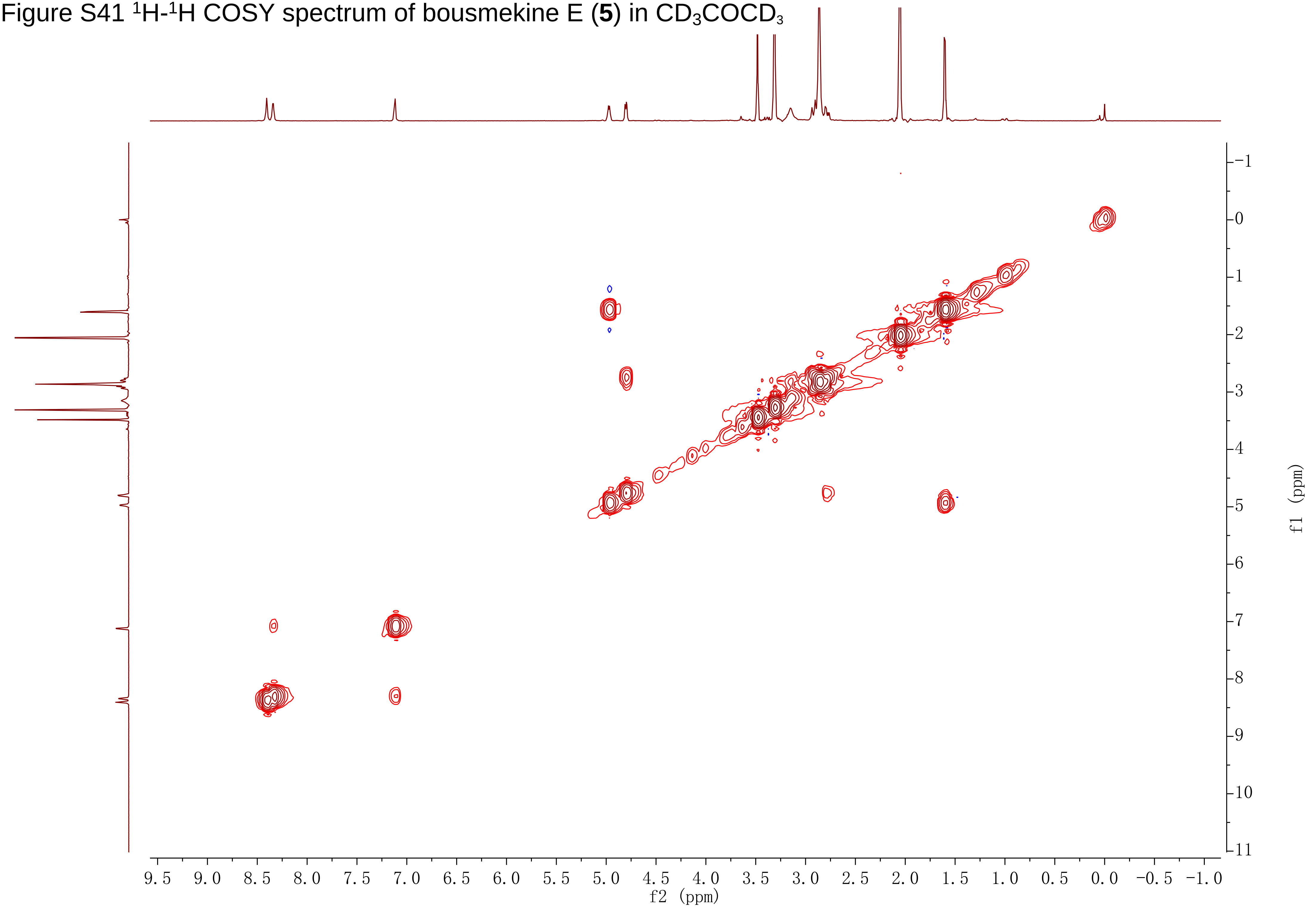

Figure S42 HMBC spectrum of bousmekine E (**5**) in CD_3COCD_3

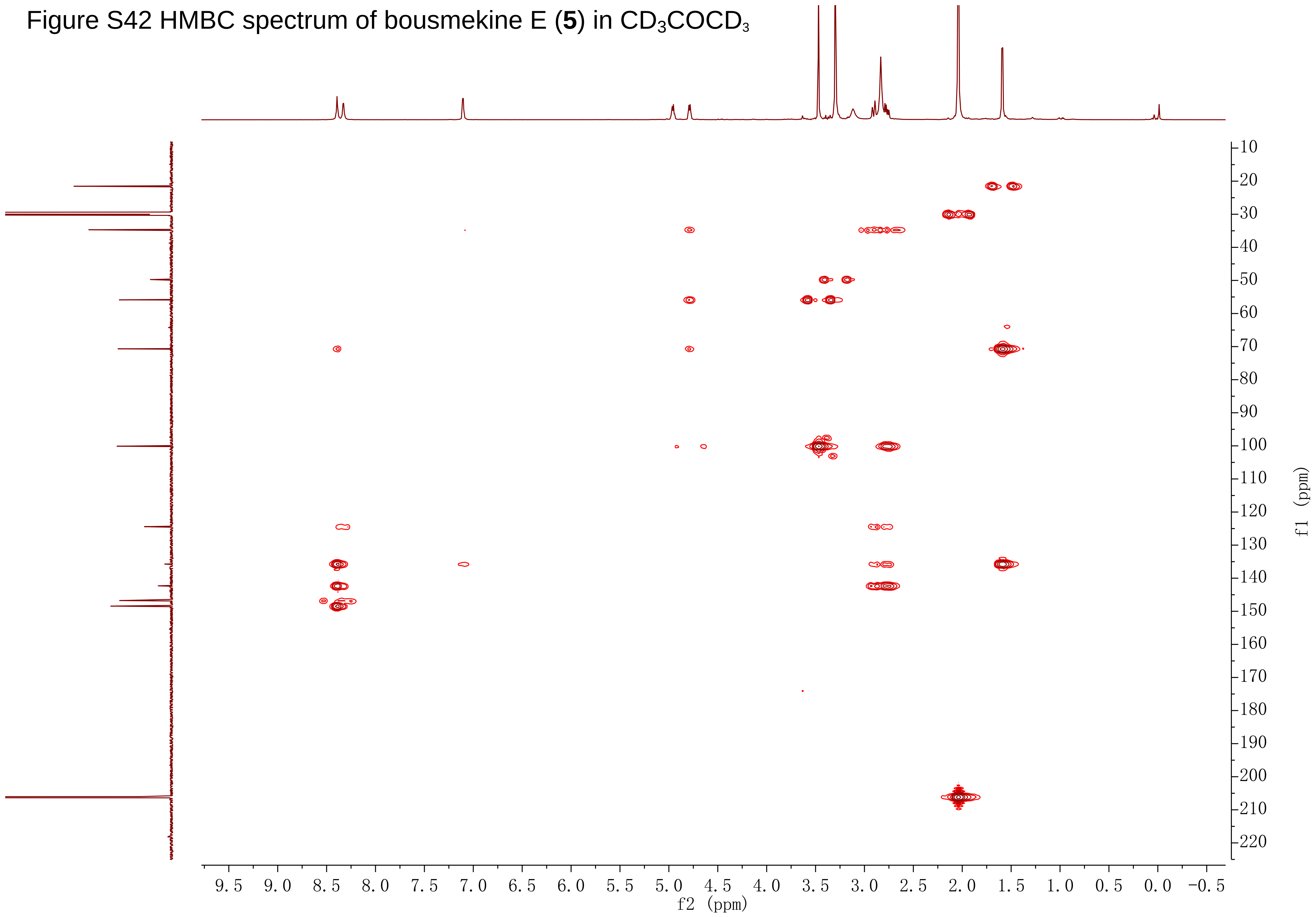

Figure S43 ROESY spectrum of bousmekine E (**5**) in CD_3COCD_3

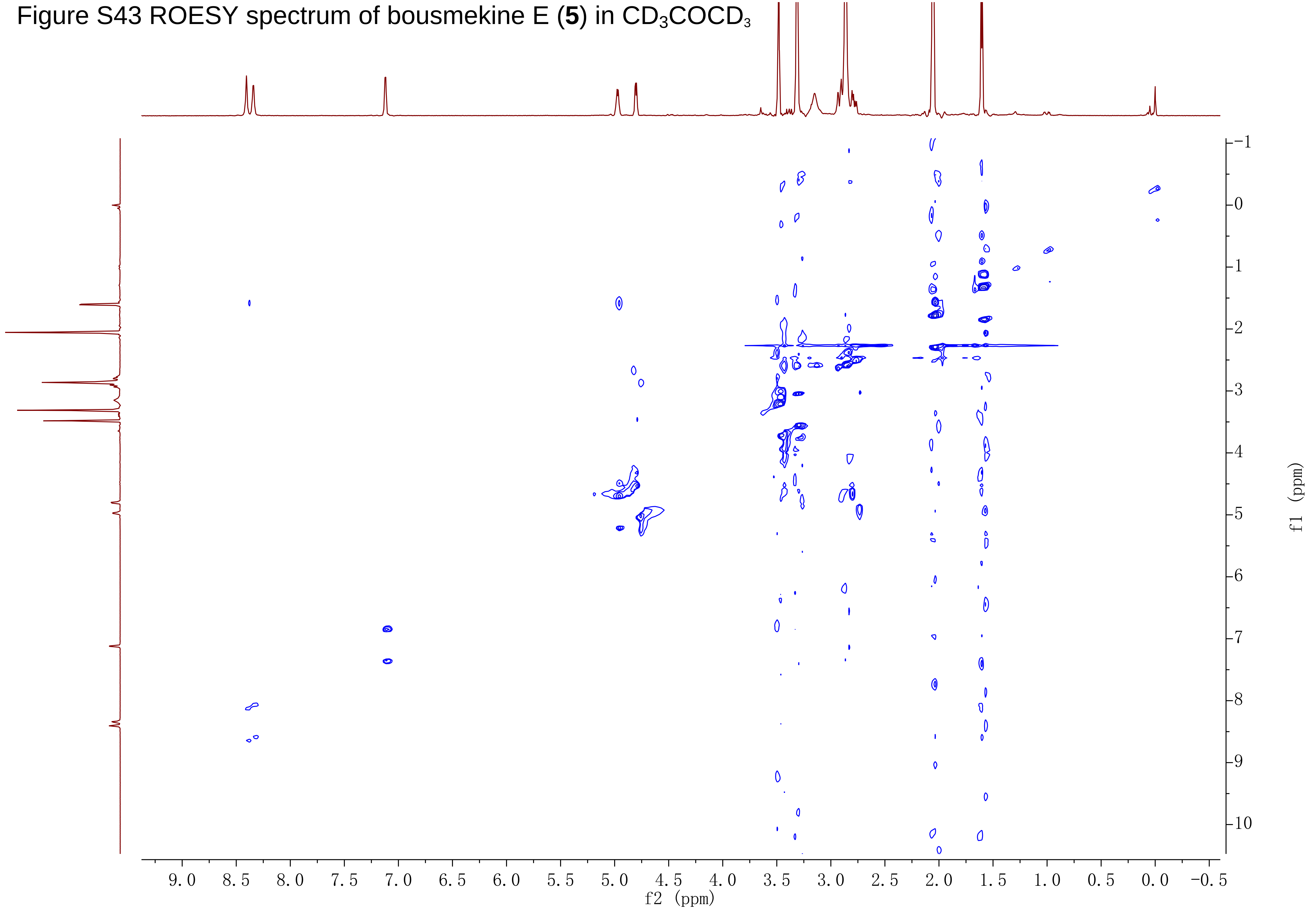

Figure S44 HRESIMS spectrums of bousmekine E (**5**)

Qualitative Analysis Report

**Data Filename**	HBM-18b.d	**Sample Name**	HBM-18b
**Sample Type**	Sample	**Position**	P1-A6
**Instrument Name**	Instrument 1	**User Name**	
**Acq Method**	s.m	**Acquired Time**	7/8/2020 2:48:31 PM
**IRM Calibration Status**	Success	**DA Method**	Default.m
**Comment**			

Sample Group **Info.**

Acquisition SW Version 6200 series TOF/6500 series Q-TOF B.05.01 (B5125.2)

User Spectra

Fragmentor Voltage	Collision Energy	Ionization Mode
135	0	ESI

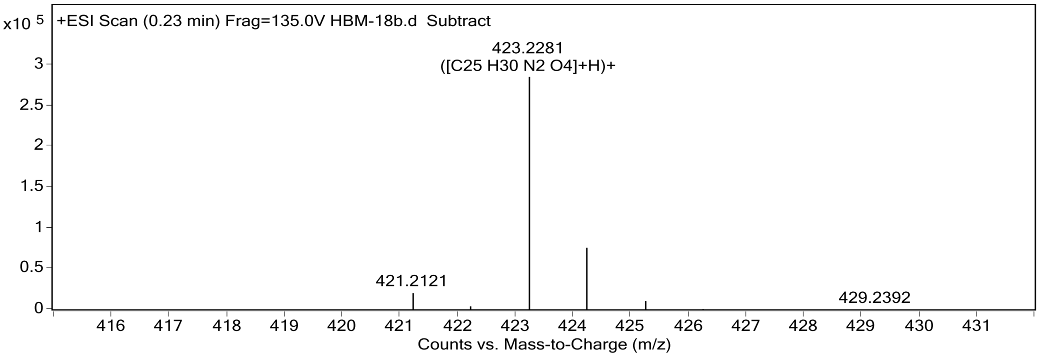

Peak List

m/z	z	Abund	Formula	Ion
367.202	1	12527.81		
421.2121	1	21170.9		
423.2281	1	285043.13	C25 H30 N2 O4	(M+H)+
424.2311	1	75900.75	C25 H30 N2 O4	(M+H)+
425.2346	1	11052.58	C25 H30 N2 O4	(M+H)+
437.2068	1	27426.22		
439.2232	1	27582.9		
745.3601	1	25192.94		
746.362	1	11636.48		
759.3755	1	15304.51		

Formula Calculator Element Limits

Element	Min	Max
C	3	60
H	0	120
O	0	30
N	0	10

Formula Calculator Results

Formula	CalculatedMass	CalculatedMz	Mz	Diff. (mDa)	Diff. (ppm)	DBE
C25 H30 N2 O4	422.2206	423.2278	423.2281	-0.30	-0.71	12.0000

--- End Of Report ---

Figure S45 IR spectrum of bousmekine E (**5**)

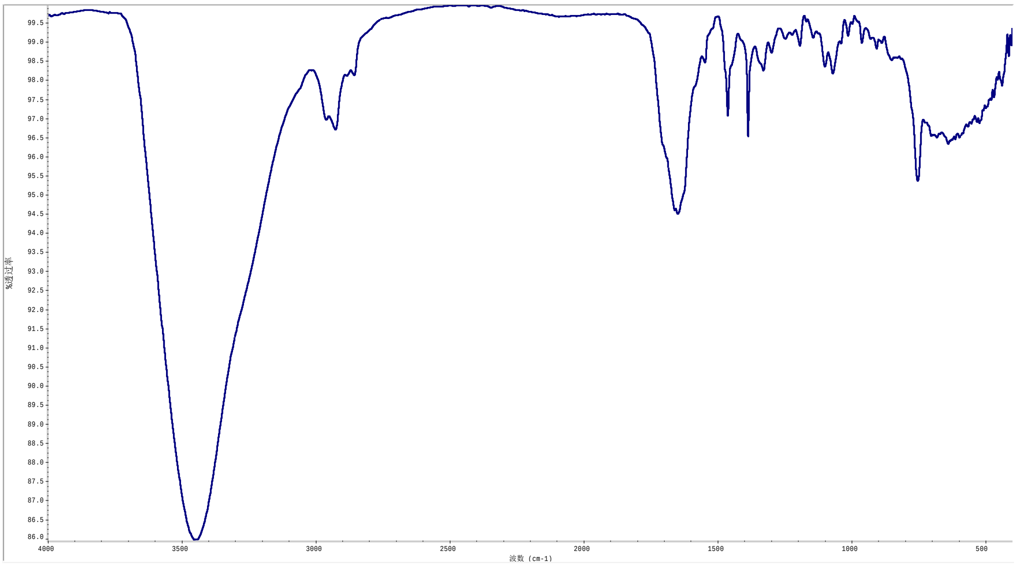

Sample Name: HZQ-20
KBr压片
采集时间: 星期五 7月 17 09:40:57 2020 (GMT+08:00)
仪器型号：NICOLET iS10
Software version: OMNIC 9.8.372

样品扫描次数: 16
背景扫描次数: 16
分辨率: 4.000
采样增益: 1.0
动镜速度: 0.4747
光阑: 80.00

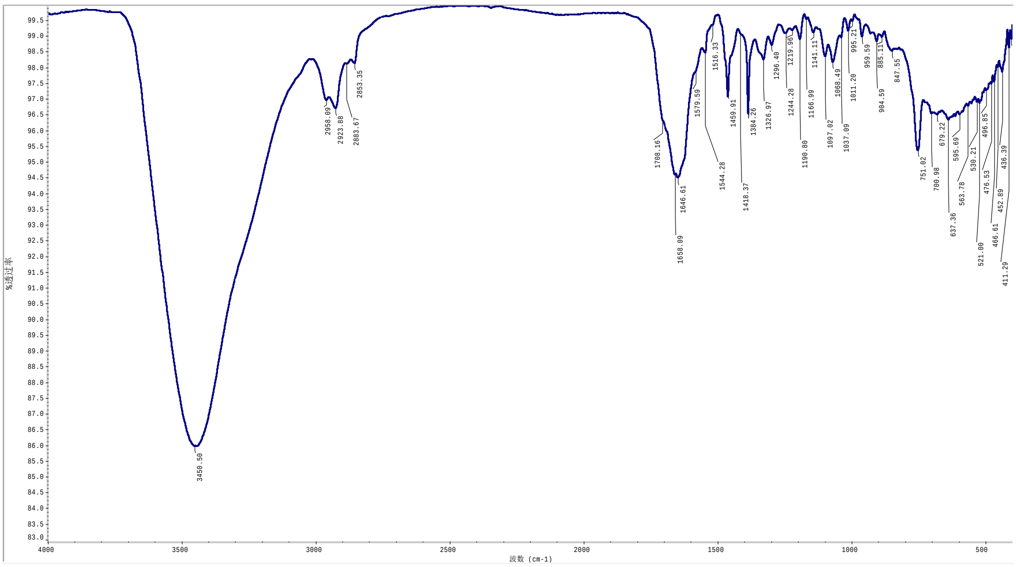

Sample Name: HZQ-20
KBr压片
采集时间: 星期五 7月 17 09:40:57 2020 (GMT+08:00)
仪器型号：NICOLET iS10
Software version: OMNIC 9.8.372

样品扫描次数: 16
背景扫描次数: 16
分辨率: 4.000
采样增益: 1.0
动镜速度: 0.4747
光阑: 80.00

2. Computational methods for ECD calculation of **1**

The CONFLEX[1,2] searches based on molecular mechanics with MMFF94S force fields were performed for **1** which gave 35 stable conformers, respectively. Selected conformers (11) with distributions higher than 1% were further optimized by the density functional theory method at the B3LYP/6-31G* level in Gaussian 09 program package,[3] leading to 4 stable geometries (ΔE < 2 kcal/mol), respectively, which were in good agreement with the ROESY data. The optimized geometries were further checked by frequency calculation and resulted in no imaginary frequencies. The ECD was calculated using TD-DFT-B3LYP/6-31G(d,p) of theory on B3LYP/6-31G(d) optimized geometries through the IEFPCM model (in MeOH). The overall calculated ECD curve was generated using SpecDis 1.60[4] with σ=0.20 ev, UV shift -5 nm, respectively.

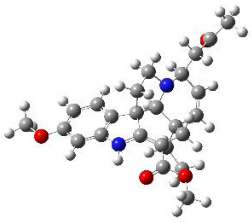
1a (36.65%)

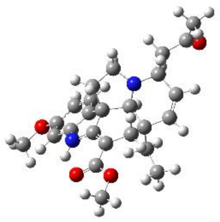
1b (27.74%)

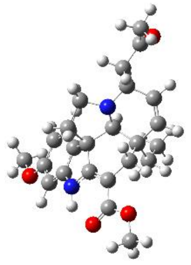
1c (19.91%)

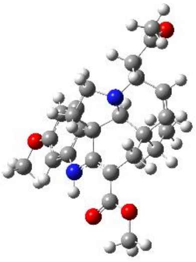
1d (15.70%)

Four stable conformers of optimized geometries of **1** at the B3LYP/6-31G** level in the MeOH phase.

Standard orientation of **1a** at B3LYP/6-31G(d) level in gas:

Center Number	Atomic Number	Atomic Type	Coordinates (Angstroms) X	Y	Z
1	6	0	-3.182827	-2.954875	0.449906
2	6	0	-4.280781	-2.186750	0.102689
3	6	0	-4.121213	-0.888436	-0.397323
4	6	0	-2.845579	-0.405222	-0.526796
5	6	0	-1.718222	-1.162879	-0.188288
6	6	0	-1.893706	-2.432223	0.291590
7	7	0	-2.442815	0.851690	-0.974854
8	6	0	-1.109582	1.028612	-0.703868
9	6	0	-0.489953	-0.352301	-0.569673

10	6	0	-0.425850	2.166944	-0.504760
11	6	0	0.989838	2.062995	0.035227
12	6	0	1.110680	0.904782	1.057101
13	6	0	0.764166	-0.413411	0.346971
14	6	0	2.534397	0.775864	1.561641
15	6	0	3.364556	-0.189764	1.216771
16	6	0	3.050920	-1.221887	0.158136
17	7	0	1.856358	-0.794931	-0.542055
18	6	0	1.291436	-1.634010	-1.573455
19	6	0	0.055032	-0.834961	-1.953719
20	6	0	0.173973	1.077421	2.282963
21	6	0	0.223335	2.433417	2.990870
22	6	0	4.226588	-1.341496	-0.820024
23	6	0	5.495127	-1.920234	-0.221443
24	6	0	6.748885	-1.782914	-1.058469
25	8	0	5.511985	-2.467669	0.839088
26	6	0	-1.110816	3.449611	-0.679534
27	8	0	-2.257743	3.579996	-1.019538
28	8	0	-0.341418	4.499916	-0.420990
29	6	0	-0.928525	5.778383	-0.567196
30	8	0	-5.559645	-2.595378	0.214883
31	6	0	-5.842369	-3.872833	0.710567
32	1	0	-3.299952	-3.945940	0.840832
33	1	0	-4.989682	-0.310685	-0.652143
34	1	0	-1.050491	-3.043004	0.562283
35	1	0	-3.043076	1.646925	-0.997698
36	1	0	1.709527	1.885045	-0.756427
37	1	0	1.270778	2.997677	0.498158
38	1	0	0.604140	-1.183612	1.104354
39	1	0	2.858441	1.498527	2.291677
40	1	0	4.329377	-0.261957	1.685297
41	1	0	2.909576	-2.194765	0.636175
42	1	0	1.027380	-2.633770	-1.213113
43	1	0	1.965819	-1.753044	-2.411583
44	1	0	-0.683648	-1.400910	-2.504595
45	1	0	0.359264	0.014429	-2.552917
46	1	0	-0.852207	0.887645	1.988382
47	1	0	0.427101	0.298136	2.997343
48	1	0	-0.408531	2.410333	3.873331
49	1	0	1.224484	2.693890	3.318852
50	1	0	-0.135146	3.234501	2.354787
51	1	0	4.436497	-0.371339	-1.261476
52	1	0	3.969069	-1.998173	-1.647494
53	1	0	6.569512	-2.086957	-2.084989

54	1	0	7.057917	-0.741399	-1.079592
55	1	0	7.541760	-2.380581	-0.630695
56	1	0	-0.152928	6.487183	-0.320873
57	1	0	-1.264678	5.929027	-1.582791
58	1	0	-1.766987	5.892601	0.104566
59	1	0	-6.917964	-3.966402	0.705290
60	1	0	-5.413962	-4.645505	0.081195
61	1	0	-5.478677	-3.993106	1.725386

Standard orientation of **1b** at B3LYP/6-31G(d) level in gas:

Center Number	Atomic Number	Atomic Type	Coordinates (Angstroms) X	Y	Z
1	6	0	-2.926598	-3.211346	0.620976
2	6	0	-4.088595	-2.524994	0.282369
3	6	0	-4.028088	-1.238389	-0.249347
4	6	0	-2.774924	-0.680493	-0.417042
5	6	0	-1.602718	-1.347121	-0.088331
6	6	0	-1.685706	-2.624104	0.426381
7	7	0	-2.474235	0.587433	-0.915630
8	6	0	-1.149452	0.860617	-0.683486
9	6	0	-0.436829	-0.471440	-0.519686
10	6	0	-0.540653	2.048018	-0.533904
11	6	0	0.888846	2.055634	-0.021211
12	6	0	1.104909	0.942693	1.034921
13	6	0	0.834574	-0.418534	0.374427
14	6	0	2.542799	0.925810	1.514839
15	6	0	3.429575	0.006263	1.185417
16	6	0	3.166866	-1.079850	0.167806
17	7	0	1.934663	-0.756258	-0.522646
18	6	0	1.409900	-1.664481	-1.515986
19	6	0	0.116526	-0.962120	-1.897649
20	6	0	0.180909	1.093636	2.273253
21	6	0	0.152276	2.472661	2.936447
22	6	0	4.330629	-1.155597	-0.828809
23	6	0	5.645562	-1.627270	-0.236424
24	6	0	6.871528	-1.440570	-1.104556
25	8	0	5.718598	-2.131448	0.842964
26	6	0	-1.315052	3.275663	-0.728813
27	8	0	-2.477126	3.318449	-1.039633
28	8	0	-0.612000	4.382745	-0.524972
29	6	0	-1.287240	5.613993	-0.693623
30	8	0	-5.242020	-3.183512	0.508542

31	6	0	-6.466166	-2.580013	0.200959
32	1	0	-3.017366	-4.197902	1.034053
33	1	0	-4.909200	-0.688201	-0.515307
34	1	0	-0.798732	-3.172073	0.691393
35	1	0	-3.125724	1.341321	-0.933980
36	1	0	1.603562	1.898246	-0.821617
37	1	0	1.116184	3.021826	0.405044
38	1	0	0.739122	-1.171941	1.159090
39	1	0	2.830476	1.692744	2.214316
40	1	0	4.405072	0.014122	1.636944
41	1	0	3.098535	-2.042914	0.680416
42	1	0	1.219722	-2.667219	-1.119399
43	1	0	2.076523	-1.765727	-2.362622
44	1	0	-0.591789	-1.593741	-2.416083
45	1	0	0.353382	-0.114732	-2.529332
46	1	0	-0.835342	0.826282	2.005712
47	1	0	0.498416	0.357067	3.006893
48	1	0	-0.460906	2.437159	3.831604
49	1	0	1.139454	2.809351	3.236095
50	1	0	-0.269807	3.227040	2.282452
51	1	0	4.467363	-0.189783	-1.307060
52	1	0	4.103125	-1.856613	-1.628192
53	1	0	6.693630	-1.795866	-2.114743
54	1	0	7.110138	-0.382621	-1.171741
55	1	0	7.710257	-1.967588	-0.671393
56	1	0	-0.554709	6.380275	-0.491665
57	1	0	-1.659611	5.708796	-1.703322
58	1	0	-2.113271	5.694416	-0.001830
59	1	0	-7.229359	-3.296466	0.465293
60	1	0	-6.616675	-1.672064	0.775345
61	1	0	-6.542367	-2.353289	-0.857215

Standard orientation of **1c** at B3LYP/6-31G(d) level in gas:

Center Number	Atomic Number	Atomic Type	Coordinates (Angstroms) X	Y	Z
1	6	0	-3.233907	-2.890764	0.242566
2	6	0	-4.337250	-2.083539	-0.058755
3	6	0	-4.162297	-0.746690	-0.475679
4	6	0	-2.869185	-0.262554	-0.570271
5	6	0	-1.740953	-1.059980	-0.275149
6	6	0	-1.929822	-2.370493	0.121596
7	7	0	-2.445488	1.026077	-0.932739

8	6	0	-1.099785	1.164553	-0.652191
9	6	0	-0.499948	-0.235675	-0.600065
10	6	0	-0.400613	2.295865	-0.372814
11	6	0	1.002574	2.146402	0.193306
12	6	0	1.109555	0.900706	1.128836
13	6	0	0.758469	-0.364519	0.314392
14	6	0	2.535263	0.741433	1.624809
15	6	0	3.386669	-0.195547	1.199599
16	6	0	3.071721	-1.146772	0.062999
17	7	0	1.865318	-0.673509	-0.607614
18	6	0	1.297828	-1.458670	-1.692397
19	6	0	0.045959	-0.645517	-2.021075
20	6	0	0.184853	1.089172	2.366710
21	6	0	0.198116	-0.039025	3.409402
22	6	0	4.252675	-1.195863	-0.925491
23	6	0	5.527325	-1.823381	-0.365244
24	6	0	6.784431	-1.625845	-1.203193
25	8	0	5.544532	-2.450704	0.674217
26	6	0	-1.080105	3.594198	-0.472047
27	8	0	-2.250504	3.749498	-0.807322
28	8	0	-0.279263	4.632702	-0.148733
29	6	0	-0.870804	5.931183	-0.213511
30	8	0	-5.632646	-2.493224	0.028170
31	6	0	-5.903964	-3.815480	0.448863
32	1	0	-3.365629	-3.914616	0.571247
33	1	0	-5.034887	-0.139941	-0.694496
34	1	0	-1.083073	-3.012094	0.353759
35	1	0	-3.034244	1.850922	-0.899675
36	1	0	1.754548	2.042628	-0.597097
37	1	0	1.258431	3.048734	0.755115
38	1	0	0.597214	-1.207603	1.007731
39	1	0	2.844756	1.426092	2.415232
40	1	0	4.369009	-0.295054	1.653171
41	1	0	2.936104	-2.166382	0.473646
42	1	0	1.035823	-2.492300	-1.389216
43	1	0	1.979369	-1.525450	-2.546343
44	1	0	-0.699633	-1.193392	-2.601210
45	1	0	0.338750	0.247840	-2.580116
46	1	0	0.490631	2.024352	2.855918
47	1	0	-0.845795	1.248701	2.033868
48	1	0	-0.387608	0.258688	4.286443
49	1	0	-0.247338	-0.961280	3.022345
50	1	0	1.212877	-0.272945	3.747650
51	1	0	4.463667	-0.186995	-1.302128

52	1	0	3.987648	-1.793806	-1.808825
53	1	0	6.601591	-1.858431	-2.258676
54	1	0	7.097674	-0.575284	-1.156578
55	1	0	7.587867	-2.256008	-0.816832
56	1	0	-0.084136	6.631647	0.070670
57	1	0	-1.223883	6.149123	-1.225443
58	1	0	-1.715822	6.011753	0.476350
59	1	0	-6.990740	-3.919506	0.444613
60	1	0	-5.470212	-4.558035	-0.234351
61	1	0	-5.529553	-4.004711	1.463872

Standard orientation of **1d** at B3LYP/6-31G(d) level in gas:

Center Number	Atomic Number	Atomic Type	Coordinates (Angstroms) X	Y	Z
1	6	0	-2.964222	-3.184661	0.371829
2	6	0	-4.134383	-2.459318	0.097802
3	6	0	-4.069088	-1.122508	-0.331629
4	6	0	-2.804150	-0.555410	-0.462565
5	6	0	-1.622104	-1.263685	-0.193702
6	6	0	-1.709744	-2.589205	0.215068
7	7	0	-2.487317	0.754547	-0.859007
8	6	0	-1.144642	0.986892	-0.642600
9	6	0	-0.449117	-0.363601	-0.560664
10	6	0	-0.519602	2.176246	-0.425136
11	6	0	0.902985	2.139401	0.104616
12	6	0	1.104039	0.948609	1.096180
13	6	0	0.828251	-0.369449	0.338345
14	6	0	2.541235	0.905862	1.578335
15	6	0	3.448646	0.012531	1.176730
16	6	0	3.186342	-0.994129	0.077164
17	7	0	1.941899	-0.629238	-0.591576
18	6	0	1.417725	-1.491428	-1.639505
19	6	0	0.108898	-0.779647	-1.977486
20	6	0	0.180542	1.130603	2.337314
21	6	0	0.272564	0.050025	3.424281
22	6	0	4.352272	-0.994134	-0.929677
23	6	0	5.674172	-1.513406	-0.373385
24	6	0	6.900904	-1.272771	-1.240157
25	8	0	5.750260	-2.098293	0.691175
26	6	0	-1.302583	3.407361	-0.523968
27	8	0	-2.498133	3.470528	-0.811434
28	8	0	-0.579469	4.522984	-0.260804

29	6	0	-1.305155	5.753997	-0.334042
30	8	0	-5.303169	-3.138294	0.284609
31	6	0	-6.523524	-2.466136	0.031098
32	1	0	-3.061731	-4.211654	0.708532
33	1	0	-4.960730	-0.543825	-0.544264
34	1	0	-0.814272	-3.167661	0.430033
35	1	0	-3.120012	1.548092	-0.814142
36	1	0	1.639864	2.031961	-0.700772
37	1	0	1.123411	3.083111	0.612183
38	1	0	0.738160	-1.195130	1.066205
39	1	0	2.811930	1.636772	2.341766
40	1	0	4.439942	-0.007177	1.621693
41	1	0	3.130809	-2.008586	0.522307
42	1	0	1.232475	-2.530763	-1.297830
43	1	0	2.093679	-1.539652	-2.500042
44	1	0	-0.603538	-1.400164	-2.526219
45	1	0	0.327952	0.113931	-2.570586
46	1	0	0.435034	2.102676	2.784322
47	1	0	-0.861422	1.216624	2.009982
48	1	0	-0.319401	0.347091	4.298107
49	1	0	-0.120590	-0.913261	3.081307
50	1	0	1.304619	-0.108989	3.756025
51	1	0	4.489415	0.014107	-1.342875
52	1	0	4.114212	-1.637140	-1.790107
53	1	0	6.714032	-1.559574	-2.282157
54	1	0	7.145745	-0.202376	-1.241790
55	1	0	7.750635	-1.833803	-0.844403
56	1	0	-0.579917	6.536681	-0.104766
57	1	0	-1.721859	5.900707	-1.335332
58	1	0	-2.124246	5.768173	0.391867
59	1	0	-7.313871	-3.190293	0.240299
60	1	0	-6.650651	-1.593478	0.687010
61	1	0	-6.599232	-2.141213	-1.016107

----

(1) Goto, H.; Osawa, E. *J. Am. Chem. Soc.* **1989**, *111*, 8950–8951.

(2) Goto, H.; Osawa, E. *J. Chem. Soc., Perkin Trans.* **1993**, *2*, 187–198.

(3) Frisch, M. J.; Trucks, G. W.; Schlegel, H. B.; Scuseria, G. E.; Robb, M.A.; Cheeseman, J. R.; Scalmani, G.; Barone, V.; Mennucci, B.; Petersson, G. A.; Nakatsuji, H.; Caricato, M.; Li, X.; Hratchian, H. P.; Izmaylov, A. F.; Bloino, J.; Zheng, G.; Sonnenberg, J. L.; Hada, M.; Ehara,M.; Toyota, K.; Fukuda, R.;Hasegawa, J.; Ishida,

M.;Nakajima, T.; Honda, Y.; Kitao, O.; Nakai, H.; Vreven, T.; Montgomery, Jr., J. A.; Peralta, J. E.; Ogliaro, F.; Bearpark,M.; Heyd, J. J.; Brothers, E.; Kudin, K. N.; Staroverov, V. N.; Keith, T.; Kobayashi, R.; Normand, J.; Raghavachari, K.; Rendell, A.; Burant, J. C.; Iyengar, S. S.; Tomasi, J.; Cossi, M.; Rega, N.; Millam, J. M.; Klene, M.; Knox, J. E.; Cross, J. B.; Bakken, V.; Adamo, C.; Jaramillo, J.; Gomperts, R.; Stratmann, R. E.; Yazyev,O.; Austin,A. J.; Cammi, R.; Pomelli,C.; Ochterski, J.W.; Martin,R. L.;Morokuma, K.; Zakrzewski,V. G.;Voth, G. A.; Salvador, P.; Dannenberg, J. J.; Dapprich, S.;Daniels, A. D.; Farkas, O.; Foresman, J. B.; Ortiz, J. V.; Cioslowski, J.; Fox, D. J. Gaussian 09, revision C.01; Gaussian, Inc.: Wallingford, CT, 2010.

(4) Bruhn, T.; Schaumlöffel, A.; Hemberger, Y.; Bringmann, G. *SpecDis*, version 1.60, University of Wuerzburg, Germany, 2012.

3. Computational method for [α] calculation of **5**

The CONFLEX[1,2] searches based on molecular mechanics with MMFF94S force fields were performed for **5** which gave 1 stable conformer, which was in good agreement with the ROESY data. The optimized geometry was further checked by frequency calculation and resulted in no imaginary frequencies. The [α] was calculated using TD-DFT-B3LYP/6-31G(d,p) of theory on B3LYP/6-31G(d) optimized geometry through the IEFPCM model (in MeOH)[3], and the calculated [α] value was -67.4.

Standard orientation of on B3LYP/6-31G(d) level in gas:

Center Number	Atomic Number	Atomic Type	Coordinates (Angstroms) X	Y	Z
1	8	0	1.376190	0.741168	-0.254551
2	6	0	1.585453	-0.551587	0.225790
3	6	0	0.534533	-1.470068	-0.360821
4	6	0	-0.839707	-0.880676	-0.146060
5	6	0	-0.987594	0.471607	0.138889
6	6	0	0.228617	1.382637	0.242176
7	6	0	-1.985413	-1.662986	-0.213088
8	6	0	-3.213669	-1.077058	0.011709
9	7	0	-3.363719	0.204310	0.303030
10	6	0	-2.276226	0.944330	0.364301
11	6	0	0.096715	2.678944	-0.544792
12	8	0	2.822311	-0.988500	-0.168334
13	6	0	3.912315	-0.291973	0.371999
14	1	0	1.520895	-0.535702	1.316740
15	1	0	0.740146	-1.569602	-1.421729
16	1	0	0.602554	-2.456786	0.083415
17	1	0	0.393612	1.622201	1.293268
18	1	0	-1.919537	-2.713863	-0.431458
19	1	0	-4.114499	-1.663323	-0.035948
20	1	0	-2.435990	1.979534	0.611213
21	1	0	-0.088775	2.464201	-1.590470
22	1	0	1.020071	3.240622	-0.465487
23	1	0	-0.710050	3.294957	-0.166124
24	1	0	4.804735	-0.805573	0.044651
25	1	0	3.878252	-0.299523	1.459037
26	1	0	3.936466	0.732321	0.024146

(1) Goto, H.; Osawa, E. *J. Am. Chem. Soc.* **1989**, *111*, 8950–8951.

(2) Goto, H.; Osawa, E. *J. Chem. Soc., Perkin Trans.* **1993**, *2*, 187–198.

(3) Frisch, M. J.; Trucks, G. W.; Schlegel, H. B.; Scuseria, G. E.; Robb, M.A.; Cheeseman, J. R.; Scalmani, G.; Barone, V.; Mennucci, B.; Petersson, G. A.; Nakatsuji, H.; Caricato, M.; Li, X.; Hratchian, H. P.; Izmaylov, A. F.; Bloino, J.; Zheng, G.; Sonnenberg, J. L.; Hada, M.; Ehara,M.; Toyota, K.; Fukuda, R.;Hasegawa, J.; Ishida, M.;Nakajima, T.; Honda, Y.; Kitao, O.; Nakai, H.; Vreven, T.; Montgomery, Jr., J. A.; Peralta, J. E.; Ogliaro, F.; Bearpark,M.; Heyd, J. J.; Brothers, E.; Kudin, K. N.; Staroverov, V. N.; Keith, T.; Kobayashi, R.; Normand, J.; Raghavachari, K.; Rendell, A.; Burant, J. C.; Iyengar, S. S.; Tomasi, J.; Cossi, M.; Rega, N.; Millam, J. M.; Klene, M.; Knox, J. E.; Cross, J. B.; Bakken, V.; Adamo, C.; Jaramillo, J.; Gomperts, R.; Stratmann, R. E.; Yazyev,O.; Austin,A. J.; Cammi, R.; Pomelli,C.; Ochterski, J.W.; Martin,R. L.;Morokuma, K.; Zakrzewski,V. G.;Voth, G. A.; Salvador, P.; Dannenberg, J. J.; Dapprich, S.;Daniels, A. D.; Farkas, O.; Foresman, J. B.; Ortiz, J. V.; Cioslowski, J.; Fox, D. J. Gaussian 09, revision C.01; Gaussian, Inc.: Wallingford, CT, 2010.
